# Microwave-assisted three-component domino reaction: Synthesis of indolodiazepinotriazoles

**DOI:** 10.3762/bjoc.9.41

**Published:** 2013-02-19

**Authors:** Rajesh K Arigela, Sudhir K Sharma, Brijesh Kumar, Bijoy Kundu

**Affiliations:** 1 Medicinal & Process Chemistry Division CSIR-Central Drug Research Institute, Lucknow-226001, India; 2 Sophisticated Analytical and Instrumental Facility, CSIR-Central Drug Research Institute, Lucknow-226001, India

**Keywords:** 2-alkynylindoles, azides, 1,3-dipolar cycloaddition, domino reaction, indolodiazepinotriazoles

## Abstract

A microwave-assisted three-component protocol involving *N*-1 alkylation of 2-alkynylindoles with epichlorohydrin, ring opening of the epoxide with sodium azide, and an intramolecular Huisgen azide–internal alkyne 1,3-dipolar cycloaddition domino sequence has been described. The efficacy of the methodology has been demonstrated by treating various 2-alkynylindoles (aromatic/aliphatic) with epichlorohydrin and sodium azide furnishing annulated tetracyclic indolodiazepinotriazoles in satisfactory yields.

## Introduction

The intermolecular Huisgen azide–alkyne 1,3-dipolar cycloaddition reaction [1–6] for the synthesis of 1,2,3-triazoles in both aqueous [7–10] and organic solvents under either metal-catalyzed [11–13] or metal-free conditions [14–16] has received increasing attention in drug discovery processes [17–18]. The ease of reaction in the intermolecular format has been successfully demonstrated by using both organic/inorganic azides as well as alkynes/diynes [19–21]. In contrast to its employment in an intermolecular format, intramolecular azide–alkyne 1,3-dipolar cycloaddition reactions have been also applied by us and others with the view to synthesize triazole-annulated polyheterocycles. Although these cyclizations have been successfully carried out in either one-pot [22–24] or multistep format [25–28], reports involving their application in a three-component domino format are scarce [29–30]. In our laboratory, we had been employing functionalized indoles for the synthesis of annulated indole-based polyheterocycles either in a multicomponent or in a one-pot format [31–35]. In this continuation, we next directed our efforts to the development of a three-component domino strategy for the synthesis of indole-based polyheterocycles by incorporating the intramolecular azide–alkyne 1,3-dipolar cycloaddition reaction as one of the domino steps. Here we propose a strategy where *N*-1 of 2-alkynylindole [36–37] can be first functionalized with epoxide by reacting 2-alkynylindole with epichlorohydrin. This can then be followed by ring opening of the oxirane by azide to furnish a bis-functionalized indole intermediate having azide and alkyne groups in close proximity. Such an intermediate may then undergo annulation following an intramolecular 1,3-dipolar cycloaddition pathway and in turn lead to the sequential formation of 7- or 5-membered diazepine and triazole rings in a single step. In this communication, we report a versatile microwave-assisted three component domino reaction to furnish annulated tetracyclic indolodiazepinotriazoles in good yields.

## Results and Discussion

**Scheme 1:**
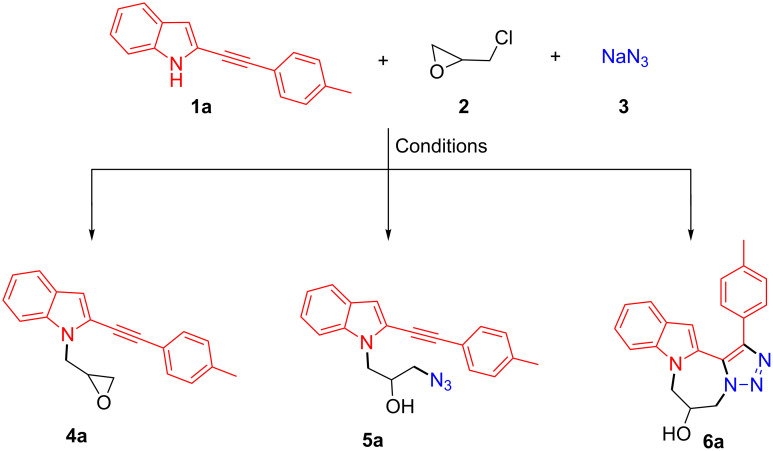
One-pot three-component domino reaction furnishing indole derivatives (**4a** and **5a**) and indolodiazepinotriazole **6a**.

We commenced our studies with the development of a one-pot three-component strategy involving the condensation of the 2-(4-methylphenylethynyl)-1*H*-indole (**1a**) with epichlorohydrin (**2**) and sodium azide (**3**, Scheme 1, Table 1). Initially, a mixture of **1a**, **2** and **3** was allowed to react both in the absence and presence of Cs_2_CO_3_ in toluene at rt. The reactants under both the conditions remained unchanged even after prolonged stirring for 15 h (Table 1, entries 1–3) and at higher temperature (110 °C).

**Table 1:** Optimization of reaction conditions for the synthesis of **6a** in a three-component domino format.

Entry	Base	Solvent	Temp (°C)	Time	Yield (%)^a^ of **4a/5a/6a**

1	–	toluene	rt	15 h	NR
2	Cs_2_CO_3_	toluene	rt	15 h	NR
3	Cs_2_CO_3_	toluene	110	15 h	NR
4	Cs_2_CO_3_	CH_3_CN	90	15 h	65/–/–
5	Cs_2_CO_3_	DMF	rt	15 h	NR
6	Cs_2_CO_3_	DMF	120	1 h	77/–/–
7	Cs_2_CO_3_	DMF	120	4 h	40/30/–
8	Cs_2_CO_3_	DMF	120	15 h	–/15/50
9	Cs_2_CO_3_	DMF	120	18 h	–/–/60
10	Cs_2_CO_3_	DMSO	120	1 h	82/–/–
11	Cs_2_CO_3_	DMSO	120	4 h	42/40/–
12	Cs_2_CO_3_	DMSO	120	10 h	–/20/52
13	Cs_2_CO_3_	DMSO	120	15 h	–/–/64
14	Cs_2_CO_3_	DMSO	120 MW	10 min	80/–/–
15	Cs_2_CO_3_	DMSO	120 MW	30 min	20/45/10^b^
16	Cs_2_CO_3_	DMSO	120 MW	1 h	–/18/42
**17**	**Cs_2_CO_3_**	**DMSO**	**120 MW**	**1.5 h**	**–/–/71**
18	Cs_2_CO_3_	DMF	120 MW	1.5 h	–/–/64
19	Cs_2_CO_3_	CH_3_CN	90 MW	1.5 h	80/–/–
20	Cs_2_CO_3_	CH_3_OH	90 MW	1.5 h	NR
21	K_2_CO_3_	DMSO	120 MW	1.5 h	–/10/54^b^
22	Na_2_CO_3_	DMSO	120 MW	1.5 h	–/12/52^b^
23	K_3_PO_4_	DMSO	120 MW	1.5 h	–/–/62
24	*t*-BuOK	DMSO	120 MW	1.5 h	–/–/65
25	DBU	DMSO	120 MW	1.5 h	–/15/48^b^
26	TEA	DMSO	120 MW	1.5 h	–/20/45^b^

^a^Isolated yields. ^b^Yields based on HPLC (C18 reversed-phase column, 150 × 4.8 mm, 5 µm). NR = no reaction.

**Scheme 2:**
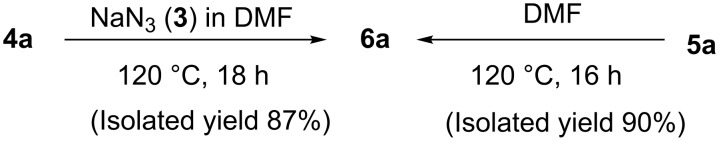
Transformation of intermediates **4a** and **5a** to **6a**.

However, a change in the nature of solvent from toluene to CH_3_CN, DMF or DMSO produced a dramatic effect on the outcome of the reaction, resulting in the formation of products comprising intermediates (**4a** and/or **5a**) and/or indole-based polyheterocycle indolodiazepinotriazole **6a**. Use of the polar solvent CH_3_CN at 90 °C for 15 h furnished a single product in 65% isolated yield, which was characterized as 2-[2-(4-methylphenyl)ethynyl]-1-(oxiran-2-ylmethyl)-1*H*-indole (**4a**, Table 1, entry 4). In contrast, use of the polar aprotic solvent DMF with high dielectric constant produced both intermediates **4a/5a** as well as the annulated product **6a**. Interestingly, a significant increase in the yield of the title compound **6a** was observed by prolonging the reaction. Carrying out the reaction in DMF at rt also failed to promote annulation even after 15 h of prolonged stirring (Table 1, entry 5). Increasing the temperature to 120 °C furnished the intermediate **4a** as a single product in 77% isolated yield within 1 h (Table 1, entry 6). Further stirring up to 4 h at 120 °C led to the partial conversion of **4a** (by ring opening of the epoxide with NaN_3_) into yet another intermediate 1-azido-3-{2-[2-(4-methylphenyl)ethynyl]-1*H*-indol-1-yl}propan-2-ol (**5a**, Table 1, entry 7) in 30% isolated yield. Nonetheless, extending the reaction times up to 15 h, led to the complete disappearance of **4a** and furnished a mixture of the intermediate **5a** in 15% isolated yield and the title compound **6a** characterized as 1-(4-methylphenyl)-6,7-dihydro-5*H*-[1,2,3]triazolo[5',1':3,4][1,4]diazepino[1,2-*a*]indol-6-ol in 50% isolated yield (Table 1, entry 8). The findings clearly suggest that the formation of indole-based annulated product **6a** in the three-component domino format occurs via **4a** and **5a** intermediacy and requires higher temperature and prolonged stirring. This was again evident from the fact that a prolonged stirring up to 18 h led to the complete disappearance of the intermediates **4a** and **5a** and afforded **6a** as a single product in 60% isolated yield (Table 1, entry 9). The role of intermediates **4a** and **5a** in the formation of **6a** was further substantiated by treating **4a** with NaN_3_ in DMF at 120 °C and by heating **5a** in DMF at 120 °C. As envisaged, both reactions furnished **6a** as a single product in 87% and 90% isolated yield, respectively (Scheme 2). Replacing DMF with yet another polar aprotic solvent, i.e., DMSO, produced similar results except for a marginal increase in the isolated yield of **6a** to 64% in 15 h (Table 1, entries 10–13).

Next, in order to reduce the reaction times and to enhance the isolated yield of the annulated product **6a**, we applied microwave conditions instead of conventional heating and monitored the progress of the reaction at different time intervals. A significant increase in the yield of **6a** resulting from the increase in the reaction times under microwave conditions was observed. Initially, a 10 min irradiation of the reaction mixture furnished the intermediate **4a** as the only product in 80% isolated yield (Table 1, entry 14), whereas a 30 min irradiation resulted in a mixture of **4a/5a/6a** in 20/45/10% yields as evident from HPLC (Table 1, entry 15). Extending exposure to microwave conditions for 1 h produced a mixture of **5a** and **6a** (Table 1, entry 16); however, a further exposure up to 1.5 h furnished the desired compound **6a** as the only product in 71% isolated yield (Table 1, entry 17). Thus, under microwave irradiation conditions, not only the isolated yield of **6a** increased from 60% under conventional heating to 71%, but the duration of reaction was also reduced from 15 h to 1.5 h. Switching the solvent from DMSO to DMF under microwave conditions furnished **6a** in slightly reduced yield (Table 1, entry 18) while the use of CH_3_CN and CH_3_OH failed to produce the desired product (Table 1, entry 19 and 20). Replacing Cs_2_CO_3_ with other bases such as K_2_CO_3_, Na_2_CO_3_, K_3_PO_4_, *t*-BuOK, DBU and TEA either produced a mixture of **5a/6a** or furnished **6a** in reduced yields (Table 1, entries 21–26). The observations clearly suggest that the formation of **6a** in the three-component format involved intermolecular *N*-1 alkylation of the 2-alkynylindole **1a** with epichlorohydrin to form **4a**, ring opening of **4a** with sodium azide to form **5a**, and finally an intramolecular Huisgen azide–internal alkyne 1,3-dipolar cycloaddition reaction.

**Scheme 3:**
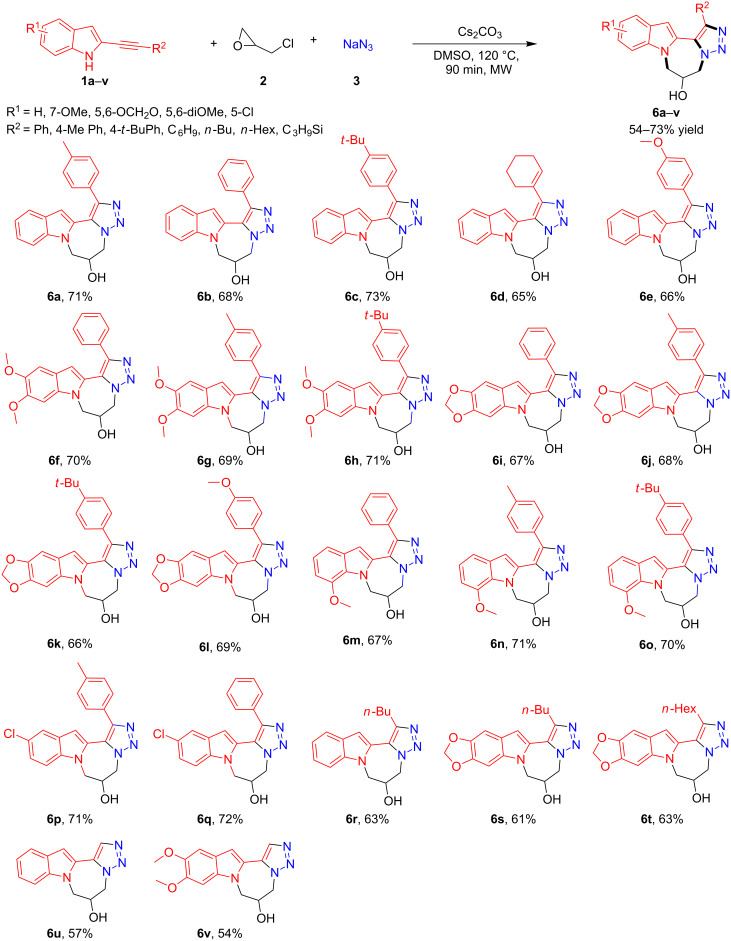
Three-component domino reaction for the synthesis of tetracyclic indolodiazepinotriazole compounds based on **6a**. Reaction conditions: **1a** (1.0 mmol), **2** (1.1 mmol), **3** (1.5 mmol) and Cs_2_CO_3_ (1.5 mmol) in DMSO (2.5 mL) at 120 °C, MW under N_2_ atmosphere.

Once the reaction conditions for the three-component format had been optimized, several 2-alkynylindoles bearing different functional groups were treated with epichlorohydrin and sodium azide in order to establish the scope and limitation of the strategy. In total 22 compounds **6a**–**v** (Scheme 3) were synthesized, with their isolated yields varying from 54–73%. The findings suggest that although the electronic properties of the substitution (R^1^) on the phenyl ring of the indole had no effect on the outcome of the isolated yield of the final products, the nature of R^2^ had a profound effect on the yields. When the aromatic group was used as R^2^, the final products **6a**–**c** and **6e**–**q** were obtained in isolated yields ranging from 66–73%, whereas substituting R^2^ with aliphatic/trimethylsilyl moieties furnished the cyclized products (**6d** and **6r**–**v**) in diminished (54–65%) isolated yields.

## Conclusion

In conclusion, we have developed a simple and efficient three-component domino reaction for the synthesis of highly substituted indolodiazepinotriazoles in good yields under microwave conditions. The domino sequence comprising *N*-1 alkylation, ring opening of the epoxide, and intramolecular Huisgen azide–internal alkyne 1,3-dipolar cycloaddition reaction, led to the generation of the diazepine and triazole rings annulated to the indole through the formation of four new sigma bonds in a single step.

## Supporting Information

File 1 Experimental section, copies of ^1^H, ^13^C NMR and HRMS spectra of starting and final compounds **1e**, **1h**, **1j**–**1l**, **1n**–**1t**, **1v**, **4a**, **5a** and **6a**–**6v**.
