# Supplementary material for: Microwave-assisted three-component domino reaction: Synthesis of indolodiazepinotriazoles
Source: Beilstein J Org Chem. 2013 Feb 19;9:401–5. doi: 10.3762/bjoc.9.41 (PMC3596043; doi:10.3762/bjoc.9.41)
Supplement: File 1 — Experimental section, copies of 1H, 13C NMR and HRMS spectra of starting and final compounds 1e, 1h, 1j–1l, 1n–1t, 1v, 4a, 5a and 6a–6v. [file Beilstein_J_Org_Chem-09-401-s001.pdf]

Supporting Information

for

Microwave-assisted three-component domino reaction: Synthesis of indolodiazepinotriazoles

Rajesh K. Arigela[1], Sudhir K. Sharma[1], Brijesh Kumar[2], and Bijoy Kundu [* 1,2,§]

Address: [1]Medicinal & Process Chemistry Division CSIR-Central Drug Research Institute, Lucknow-226001, India and [2]Sophisticated Analytical and Instrumental Facility, CSIR-Central Drug Research Institute, Lucknow-226001, India

[§]Tel.: +91 522 2612411-18-Ext 4383; fax: +91 522 2623405

Email: Bijoy Kundu - bijoy_kundu@yahoo.com

[*]Corresponding author

Experimental section, copies of ^{1}H, ^{13}C NMR and HRMS spectra of starting and final compounds **1e**, **1h**, **1j**–**1l**, **1n**–**1t**, **1v**, **4a**, **5a** and **6a**–**6v**

Table of Contents .... Page

Experimental section S2

General considerations S2

Experimental procedures and analytical data S2

^{1}H NMR and ^{13}C NMR spectra S15

HRMS spectra S53

References S71

Experimental section

General considerations

All reagents and solvents were purchased from commercial sources and used without purification. NMR spectra were recorded with a 200, 300, 400 MHz spectrometers for ^{1}H NMR and 50, 75, 100 MHz for ^{13}C NMR. Chemical shifts δ are given in ppm relative to the residual signals of tetramethylsilane in $CDCl_3$ or deuterated solvent $CDCl_3$/DMSO-d_6 for ^{1}H and ^{13}C NMR. Multiplicities are reported as follows: singlet (s), doublet (d), doublet of doublets (dd), doublet of triplets (dt), triplet (t), quartet (q), multiplet (m). HRMS were obtained using the electrospray ionization (ESI) technique and a time-of-flight (TOF) analyzer. Microwave irradiation was carried out with Initiator 2.5 Microwave Synthesizers from Biotage. All the reactions were performed in special 10 mL glass vessels under an atmosphere of nitrogen. The temperature was fixed to 120 °C and maintained for 1.5 h. Column chromatography was performed using silica gel (100–200 mesh) as the stationary phase. All reactions were monitored by thin layer chromatography (TLC). The purity and characterization of these compounds were further established using high-resolution EI mass spectrometry. Melting points were measured on a capillary melting point apparatus and are uncorrected.

Experimental procedures and analytical data

Starting materials **1a–v** were prepared according to the literature procedures [1-3].

For characterization of 2-[2-(4-methylphenyl)ethynyl]-1*H*-indole (**1a**), 2-(2-phenylethynyl)-1*H*-indole (**1b**), 2-{2-[4-(*tert*-butyl)phenyl]ethynyl}-1*H*-indole (**1c**), 2-[2-(cyclohex-1-en-1-yl)ethynyl]-1*H*-indole (**1d**), see reference [1].

2-[2-(4-Methoxyphenyl)ethynyl]-1*H*-indole (1e):

Yield (0.554g, 62%); yellow solid; R_f 0.45 (20:80 ethyl acetate/hexanes); mp 161–163 °C;[Lit. [3,4] 160–163 °C]; FT-IR (KBr) 3416, 2927, 1598, 1483, 1241, 735 cm^{-1}; ^{1}H NMR (300 MHz, $CDCl_3$): δ 8.24 (s, 1H), 7.62 (d, *J* = 9.0 Hz, 1H), 7.51 (d, *J* = 6.0 Hz, 2H), 7.35 (d, *J* = 6.0 Hz, 1H), 7.28-7.23 (m, 1H), 7.17-7.13 (m, 1H), 6.92 (d, *J* = 9.0 Hz, 2H), 6.83 (s, 1H), 3.86 (s, 3H) ppm; ^{13}C NMR (50 MHz, DMSO-d_6): δ 159.6, 136.3, 132.8, 127.2, 122.6, 120.2, 119.7, 118.5, 114.5, 113.8, 111.1, 107.1, 91.7, 81.3, 55.2 ppm; HRMS (ESI) calcd for $C_{17}H_{14}NO$ [M + H] 248.1075 found 248.1087.

For characterization of 5,6-dimethoxy-2-(2-phenylethynyl)-1*H*-indole (**1f**) and 5,6-dimethoxy-2-[2-(4-methylphenyl)ethynyl]-1*H*-indole (**1g**), see reference [1].

2-{2-[4-(*tert*-Butyl)phenyl]ethynyl}-5,6-dimethoxy-1*H*-indole (1h):

Yield (0.578g, 58%); yellow solid; R_f 0.35 (20:80 ethyl acetate/hexanes); mp 140–142 °C; FT-IR (KBr) 3339, 2953, 2362, 1477, 1199 cm^{-1}; ^{1}H NMR (300 MHz, $CDCl_3$): δ 8.20 (s, 1H), 7.45-7.43 (m, 2H), 7.37-7.35 (m, 2H), 7.05-7.00 (m, 1H), 6.79-6.70 (m, 2H), 3.90-3.93 (m, 6H), 1.33 (s, 9H) ppm; ^{13}C NMR (50 MHz, $CDCl_3$): δ151.7, 148.4, 145.8, 134.2, 131.1, 128.6, 125.5, 121.0, 120.0, 117.5, 108.5, 102.0, 94.0, 92.3, 81.7, 56.3, 56.2, 34.9, 31.2 ppm; HRMS (ESI) calcd for $C_{22}H_{24}NO_2$ [M + H] 334.1807 found 334.1811.

For characterization of 6-(2-phenylethynyl)-5*H*-[1,3]dioxolo[4,5-*f*]indole (**1i**): see reference [1].

6-(2-(4-Methylphenylethynyl)-5*H*-[1,3]dioxolo[4,5-*f*]indole (1j):

Yield (0.536g, 62%); light yellow solid; R_f 0.52 (20:80 ethyl acetate/hexanes); mp 166–168 °C; FT-IR (KBr) 3423, 2920, 1649, 1472, 1338, 818 cm^{-1}; ^{1}H NMR (300 MHz, $CDCl_3$): δ 8.10 (s, 1H), 7.41-7.39 (m, 2H), 7.17-7.14 (m, 2H), 6.94 (s, 1H), 6.77 (s, 1H), 6.68 (s, 1H), 5.94 (s, 2H), 2.37 (s, 3H) ppm; ^{13}C NMR (75 MHz, DMSO-d_6): δ 145.5, 143.0, 138.4, 131.6, 130.8, 129.4, 121.1, 116.6, 107.8, 100.4, 98.3, 91.6, 91.4, 82.4, 21.0 ppm; HRMS (ESI) calcd for $C_{18}H_{14}NO_2$ [M + H] 276.1025 found 276.1032.

6-{2-[4-(*tert*-Butyl)phenyl]ethynyl}-5*H*-[1,3]dioxolo[4,5-*f*]indole (1k):

Yield (0.717g, 72%); light yellow solid; R_f 0.53 (20:80 ethyl acetate/hexanes); mp 152–154 °C;

FT-IR (KBr) 3098, 1638, 1514, 1467, 1215 cm^{-1}; ^{1}H NMR (300 MHz, $CDCl_3$): δ 8.10 (s, 1H), 7.46-7.35 (m, 4H), 6.94 (s, 1H), 6.77 (s, 1H), 6.68 (d, *J* = 0.9 Hz, 1H), 5.93 (s, 2H) , 1.32 (s, 9H) ppm; ^{13}C NMR (75 MHz, $CDCl_3$): δ 151.7, 146.2, 143.6, 131.3, 131.0, 125.4, 121.8, 119.7, 117.6, 108.8, 100.7, 98.8, 92.3, 91.4, 81.2, 34.8, 31.17 ppm; HRMS (ESI) calcd for $C_{21}H_{20}NO_2$ [M + H] 318.1494 found 318.1496.

6-[2-(4-Methoxyphenyl)ethynyl]-5*H*-[1,3]dioxolo[4,5-*f*]indole (1l):

Yield (0.475g, 52%); light yellow solid; R_f 0.43 (20:80 ethyl acetate/hexanes); mp 158–160 °C; FT-IR (KBr) 3409, 2898, 1602, 1469, 1241, 1028 cm^{-1}; ^{1}H NMR (300 MHz, DMSO-d_6): δ 11.42 (s, 1H), 7.46 (d, *J* = 9.0 Hz, 2H), 7.00-6.96 (m, 3H), 6.81 (s, 1H), 6.60 (s, 1H), 5.94 (s, 2H), 3.79 (s, 3H) ppm; ^{13}C NMR (50 MHz, DMSO-d_6): δ 159.4, 145.4, 142.8, 132.6, 131.4, 121.0, 116.7, 114.5, 114.1, 107.5, 100.4, 98.2, 91.5, 91.2, 81.5, 55.2 ppm; HRMS (ESI) calcd for $C_{18}H_{14}NO_3$ [M + H] 292.0974 found 292.0978.

For characterization of 7-methoxy-2-(2-phenylethynyl)-1*H*-indole (**1m**) see reference [1].

7-Methoxy-2-[2-(4-methylphenyl)ethynyl]-1*H*-indole (1n):

Yield (0.652g, 76%); Yellow solid; R_f 0.60 (20:80 ethyl acetate/hexanes); mp 140–142 °C; FT-IR (KBr) 3018, 1635, 1528, 1450, 1216 cm^{-1}; ^{1}H NMR (300 MHz, $CDCl_3$): δ 8.48 (s, 1H), 7.43-7.41 (m, 2H), 7.23-7.14 (m, 3H), 7.03 (t, *J* = 9.0 Hz, 1H), 6.77 (d, *J* = 1.8 Hz, 1H), 6.65 (d, *J* = 9.0 Hz, 1H), 3.94 (s, 3H), 2.36 (s, 3H) ppm; ^{13}C NMR (50 MHz, $CDCl_3$): δ 145.8, 138.8, 131.4, 129.3, 129.1, 127.0, 120.9, 119.7, 118.7, 113.5, 108.8, 103.5, 92.4, 81.3, 55.5, 21.6 ppm; HRMS (ESI) calcd for $C_{18}H_{16}NO$ [M + H] 262.1232 found 262.1240.

2-{2-[4-(*tert*-Butyl)phenyl]ethynyl}-7-methoxy-1*H*-indole (1o):

Yield (0.758g, 76%); green oil; R_f 0.60 (20:80 ethyl acetate/hexanes); FT-IR (neat) 3369, 2940, 2101, 1461, 1259 cm^{-1}; ^{1}H NMR (300 MHz, $CDCl_3$): δ 8.53 (s, 1H), 7.48-7.36 (m, 4H), 7.22 (t, *J* = 9.0 Hz, 1H), 7.03 (t, *J* = 9.0 Hz, 1H), 6.77 (d, *J* = 1.8 Hz, 1H), 6.66 (d, *J* = 9.0 Hz, 1H), 3.95 (s, 3H), 1.32 (s, 9H) ppm; ^{13}C NMR (50 MHz, $CDCl_3$): δ 152.0, 145.8, 131.3, 129.1, 127.0, 125.6, 120.9, 119.7, 118.8, 113.5, 108.8, 103.0, 92.4, 81.3, 55.5, 34.9, 31.3 ppm; HRMS (ESI) calcd for $C_{21}H_{22}NO$ [M + H] 304.1701 found 304.1708.

5-Chloro-2-[2-(4-methylphenyl)ethynyl]-1*H*-indole (1p):

Yield (0.519g, 61%); yellow solid; R_f 0.53 (20:80 ethyl acetate/hexanes); mp 138–140 °C; FT-IR (KBr) 3414, 1395, 1064, 791, 490 cm^{-1}; ^{1}H NMR (300 MHz, $CDCl_3$): δ 8.24 (s, 1H), 7.55 (s, 1H), 7.42 (d, J = 6.0 Hz, 2H), 7.25-7.16 (m, 4H), 6.73 (s, 1H), 2.37 (s, 3H) ppm; ^{13}CNMR (100 MHz, $CDCl_3$): δ 134.5, 131.6, 131.5, 129.4, 128.9, 128.6, 126.3, 123.9, 122.4, 120.2, 111.8, 108.0, 93.2, 81.3, 21.6 ppm; HRMS (ESI) calcd for $C_{17}H_{13}ClN$ [M + H] 266.0737 found 266.0720.

5-Chloro-2-(2-phenylethynyl)-1*H*-indole (1q):

Yield (0.472g, 58%); yellow solid; R_f 0.51 (20:80 ethyl acetate/hexanes); mp 142–144 °C; FT-IR (KBr) 3411, 2920, 1439, 1393, 793 cm^{-1}; ^{1}H NMR (300 MHz, $CDCl_3$): δ 8.29 (s, 1H), 7.56-7.52 (m, 3H), 7.37-7.36 (m, 3H), 7.25-7.19 (m, 1H), 7.17-7.16 (m, 1H), 6.75 (s, 1H) ppm; ^{13}C NMR (50 MHz, $CDCl_3$): δ 135.2, 130.5, 127.5, 125.4, 124.9, 124.6, 122.2, 119.8, 116.6, 116.1, 115.3, 107.8, 104.3, 104.0, 89.4, 76.6 ppm; HRMS (ESI) calcd for $C_{16}H_{11}ClN$ [M + H] 252.0580 found 252.0557.

2-Hex-1-ynyl-1*H*-indole (1r):

Yield (0.442g, 62%); green oil; R_f 0.65 (20:80 ethyl acetate/hexanes); FT-IR (neat) 3405, 2940, 1645, 1461, 753 cm^{-1}; ^{1}H NMR (300 MHz, $CDCl_3$): δ 8.03 (s, 1H), 7.54 (d, J = 6.0 Hz, 1H), 7.26-7.17 (m, 1H), 7.15-7.05 (m, 2H), 6.64 (s, 1H), 2.44 (t, J = 6.0 Hz, 2H), 1.65-1.44 (m, 4H), 0.95 (t, J = 6.0 Hz, 3H) ppm; ^{13}C NMR (50 MHz, $CDCl_3$): δ 135.8, 127.9, 123.0, 120.6, 120.4, 119.7, 110.7, 107.5, 94.0, 73.0, 30.7, 22.1, 19.3, 13.7 ppm; HRMS (ESI) calcd for $C_{14}H_{16}N$ [M + H] 198.1283 found 198.1272.

6-Hex-1-ynyl-5*H*-[1,3]dioxolo[4,5-*f*]indole (1s):

Yield (0.469g, 62%); Light yellow solid; R_f 0.70 (20:80 ethyl acetate/hexanes); mp 104-106 °C; FT-IR (KBr) 3390, 2927, 1465, 1181, 516 cm^{-1}; ^{1}H NMR (300 MHz, $CDCl_3$): δ 8.01 (s, 1H), 6.93 (s, 1H), 6.76 (s, 1H), 6.55 (s, 1H), 5.94 (s, 2H), 2.46 (t, J = 6.0 Hz, 2H), 1.64-1.46 (m, 4H), 0.97 (m, 3H) ppm; ^{13}C NMR (100 MHz, $CDCl_3$): δ 145.8, 143.5, 130.8, 121.7, 118.2, 107.7, 100.7, 98.8, 93.4, 91.5, 73.1, 30.7, 22.1, 19.3, 13.6 ppm; HRMS (ESI) calcd for $C_{15}H_{16}NO_2$ [M + H] 242.1181 found 242.1187.

6-Oct-1-ynyl-5*H*-[1,3]dioxolo[4,5-*f*] indole (1t):

Yield (0.508g, 60%); light yellow solid; R_f 0.72 (20:80 ethyl acetate/hexanes); mp 110–112 °C; FT-IR (KBr) 3391, 2925, 1464, 1181, 941, 838, 516 cm^{-1}; ^1H NMR (300 MHz, $CDCl_3$): δ 8.05 (s, 1H), 6.94 (s, 1H), 6.76 (s, 1H), 6.55 (s, 1H), 5. 94 (s, 2H), 2.46 (t, J = 6.0 Hz, 2H), 1.68-1.46 (m, 6H), 1.31-1.26 (m, 2H), 0.97 (m, 3H) ppm; ^{13}C NMR (50 MHz, $CDCl_3$): δ 145.9, 143.5, 130.8, 121.7, 118.3, 107.7, 100.7, 98.9, 93.5, 91.5, 73.1, 31.5, 28.7, 22.7, 19.7, 14.2 ppm; HRMS (ESI) calcd for $C_{17}H_{20}NO_2$ [M + H] 270.1494 found 270.1495.

For characterization of 2-(2-(trimethylsilyl)ethynyl)-1*H*-indole (**1u**), see reference [1].

5,6-Dimethoxy-2-trimethylsilanylethynyl-1*H*-indole (1v):

Yield (0.515 g, 63%); yellow solid; R_f 0.53 (20:80 ethyl acetate/hexanes); mp 158–160°C; FT-IR (KBr) 3328, 2956, 2152,, 1327, 843 cm^{-1}; ^1H NMR (300 MHz, $CDCl_3$): δ 8.05 (s, 1H), 6.98 (s, 1H), 6.76 (s, 1H), 6.66 (s, 1H), 3.90 (s, 6H), 0.26 (s, 9H) ppm; ^{13}C NMR (50 MHz, $CDCl_3$): δ 148.6, 145.8, 130.7, 120.3, 117.0, 109.3, 101.9, 97.7, 97.6, 93.8, 56.2, 56.1, 0.02 ppm; HRMS (ESI) calcd for $C_{15}H_{20}NO_2Si$ [M + H] 274.1263 found 274.1270.

Typical procedure for intermediate 4a:

To a stirred solution of 2-[2-(4-methylphenyl)ethynyl]-1*H*-indole (**1a**) (0.150 g, 1.0 mmol), epichlorohydrin (0.065 g, 1.1 mmol) in CH_3CN (5 mL) and Cs_2CO_3 (0.318 g, 1.5 mmol) were added under an N_2 atmosphere and the reaction mixture was stirred at 90 °C for 15 h. After cooling to room temperature the reaction mixture was extracted with ethyl acetate (3 x 25 mL). The organic layer was washed with brine solution, dried over anhydrous Na_2SO_4 and the solvent was evaporated in vacuo. The residue was purified by column chromatography, eluting with ethyl acetate/hexanes to afford **4a**.

2-[2-(4-Methylphenyl)ethynyl]-1-(oxiran-2-ylmethyl)-1*H*-indole (4a):

Yield (0.149g, 80%); yellow oil; R_f 0.74 (20:80 ethyl acetate/hexanes); FT-IR (neat) 3405, 2927, 1635, 1387 cm^{-1}; ^1H NMR (300 MHz, $CDCl_3$): δ 7.58 (d, J = 9.0 Hz, 1H), 7.44 (d, J = 9.0 Hz, 2H), 7.38 (d, J = 9.0 Hz, 1H), 7.26 (t, J = 6.0 Hz, 1H), 7.19-7.09 (m, 3H), 6.84 (s, 1H), 4.48-4.46 (m, 2H), 3.32-3.30 (m, 1H), 2.80-2.88 (m, 1H), 2.64-2.62 (m, 1H), 2.37 (s, 3H) ppm; ^{13}C NMR (100 MHz, $CDCl_3$): δ 139.1, 137.3, 131.4, 129.3, 127.6, 123.4, 121.9, 121.0, 119.5, 109.8, 95.8,

80.2, 50.8, 46.1, 46.0, 21.6 ppm; HRMS (ESI) calcd for $C_{20}H_{18}NO$ [M + H] 288.1388 found 288.1376.

Typical synthetic procedure for intermediate 5a:

A solution of 2-[2-(4-methylphenyl)ethynyl]-1-(oxiran-2-ylmethyl)-1*H*-indole (**4a**) (0.150 g, 1.0 mmol) and sodium azide (0.034 g, 1.5 mmol) in DMF (5 mL) under N_2 atmosphere was heated at 120 °C for 4 h under stirring. The reaction mixture was cooled to room temperature and extracted with ethyl acetate (3 x 25 mL). The organic layer was washed with brine solution and dried over anhydrous Na_2SO_4, and the solvent was evaporated in vacuo. The residue was purified by column chromatography, eluting with ethyl acetate/hexanes to afford **5a**.

1-Azido-3-{2-[2-(4-methylphenyl)ethynyl]-1*H*-indol-1-yl}propan-2-ol (5a):

Yield (0.138g, 80%); white solid; R_f 0.64 (20:80 ethyl acetate/hexanes); mp 135–137 °C; FT-IR (KBr) 3426, 2254, 1644, 1024, 770 cm^{-1}; ^1H NMR (300 MHz, $CDCl_3$): δ 7.58 (d, *J* = 9.0 Hz, 1H), 7.44 (d, *J* = 9.0 Hz, 2H), 7.36 (d, *J* = 6.0 Hz, 1H), 7.28-7.19 (m, 1H), 7.16-7.10 (m, 3H), 6.84 (s, 1H), 4.39-4.37 (m, 2H), 4.27-4.26 (m, 1H), 3.47-3.42 (m, 1H), 3.37-3.31 (m, 1H), 2.37 (s, 3H), 2.23 (d, *J* = 3.0 Hz, 1H) ppm; ^{13}C NMR (100 MHz, $CDCl_3$): δ 139.2, 137.2, 131.4, 129.4, 127.5, 123.5, 121.8, 121.2, 120.6, 119.3, 109.7, 108.3, 96.2, 80.2, 70.3, 54.3, 47.5, 21.6 ppm; HRMS (ESI) calcd for $C_{20}H_{19}N_4O$ [M + H] 331.1559 found 331.1547.

General Procedure for the domino cyclizations:

A mixture of 2-ethynyl-1*H*-indole derivatives **1a–v** (0.150 g, 1.0 mmol), epichlorohydrin (1.1 mmol), sodium azide (1.5 mmol) and Cs_2CO_3 (1.5 mmol) in DMSO (5 mL) under N_2 atmosphere was placed in a 10 mL microwave vial containing a stirring bar. The sealed reaction mixture was heated at 120 °C for 1.5 h in a microwave (Biotage). The reaction mixture was cooled to ambient temperature and extracted with ethyl acetate (3 x 25 mL). The organic layer was washed with brine solution and dried over anhydrous Na_2SO_4, and the solvent was evaporated in vacuo. The residues were purified by column chromatography, eluting with ethyl acetate/hexanes to afford **6a–v**.

1-(4-Methylphenyl)-6,7-dihydro-5*H*-[1,2,3]triazolo[5',1':3,4][1,4]diazepino[1,2-*a*]indol-6-ol (6a):

Yield (0.151g, 71%); white solid; R_f 0.30 (1:1 ethyl acetate/hexanes); mp 186–188 °C; FT-IR (KBr) 3286, 2927, 1461, 1346, 1252, 823 cm^{-1}; ^1H NMR (300 MHz, DMSO-d_6): δ 7.79 (d, J = 6.0 Hz, 2H), 7.64 (t, J = 9.0 Hz, 2H), 7.33-7.25 (m, 3H), 7.11 (t, J = 7.2 Hz, 1H), 6.81 (s, 1H), 5.85 (d, J = 6.0 Hz, 1H), 4.67-4.65 (m, 1H), 4.59-4.46 (m, 2H), 4.29-4.22 (m, 1H), 4.01-3.95 (m, 1H), 2.34 (s, 3H) ppm; ^{13}C NMR (50 MHz, DMSO-d_6): δ 143.8, 137.7, 137.6, 129.2, 127.7, 127.0, 126.6, 126.3, 126.2, 122.7, 120.9, 120.0, 110.0, 103.4, 68.7, 53.2, 47.3, 20.8 ppm; HRMS (ESI) calcd for $C_{20}H_{19}N_4O$ [M + H] 331.1558 found 331.1557.

1-Phenyl-6,7-dihydro-5*H*-[1,2,3]triazolo[5',1':3,4][1,4]diazepino[1,2-*a*]indol-6-ol (6b):

Yield (0.148g, 68%); white solid; R_f 0.28 (1:1 ethyl acetate/hexanes); mp 192–194 °C; FT-IR (KBr) 3397, 2934, 1627, 1077 cm^{-1}; ^1H NMR (300 MHz, DMSO-d_6): δ 7.90 (d, J = 7.2 Hz, 2H), 7.63 (t, J = 9.0 Hz, 2H), 7.46-7.38 (m, 3H), 7.26(t, J = 6 Hz, 1H), 7.29 (t, J = 7.2 Hz, 1H), 6.83 (s, 1H), 5.85 (d, J = 3.0 Hz, 1H), 4.61 (m, 1H), 4.56-4.45 (m, 2H), 4.28-4.22 (m, 1H), 4.00-3.94 (m, 1H) ppm; ^{13}C NMR (50 MHz, DMSO-d_6): δ 143.6, 137.6, 130.5, 128.6, 126.9, 126.6, 126.0, 122.8, 120.9, 120.0, 110.1, 103.6, 68.7, 53.2, 47.3 ppm; HRMS (ESI) calcd for $C_{19}H_{17}N_4O$ [M + H] 317.1402 found 317.1402.

1-(4-(*tert*-Butyl)phenyl)-6,7-dihydro-5*H*-[1,2,3]triazolo[5',1':3,4][1,4]diazepino[1,2-*a*]indol-6-ol (6c):

Yield (0.150g, 73%); white solid; R_f 0.32 (1:1 ethyl acetate/hexanes); mp 200–202 °C; FT-IR (KBr) 3324, 2955, 1609, 1249, 738 cm^{-1}; ^1H NMR (300 MHz,$CDCl_3$): δ 7.88 (d, J = 9.0, Hz, 2H), 7.66 (d, J = 6.0, Hz, 1H), 7.47-7.39 (m, 3H), 7.32 (t, J = 9.0, Hz, 1H), 7.18 (t, J = 6.0, Hz, 1H), 6.90 (s, 1H), 4.77 (s, 1H), 4.67-4.60 (m, 1H), 4.47-4.37 (m, 2H), 4.17-4.10 (m, 1H), 3.28 (bs, 1H), 1.33 (s, 9H) ppm; ^{13}C NMR (50 MHz,$CDCl_3$): δ 151.9, 144.8, 137.9, 127.6, 127.3, 127.0, 126.7, 126.2, 125.7, 123.5, 121.5, 120.6, 109.5, 104.8, 70.1, 53.6, 47.8, 34.8, 31.3 ppm; HRMS (ESI) calcd for $C_{23}H_{25}N_4O$ [M + H] 373.2028, found 373.2028.

1-Cyclohex-1-en-1-yl-6,7-dihydro-5*H*-[1,2,3]triazolo[5',1':3,4][1,4]diazepino[1,2-*a*]indol-6-ol (6d):

Yield (0.141g, 65%); white solid; R_f 0.36 (1:1 ethyl acetate/hexanes); mp 172–174 °C; FT-IR (KBr) 3410, 2927, 1645, 1447, 1031 cm^{-1}; ^1H NMR (300 MHz, DMSO-d_6): δ 7.64 (t, *J* = 6.0 Hz, 2H), 7.25 (t, *J* = 9.0 Hz, 1H), 7.10 (t, *J* = 9.0 Hz, 1H), 6.88 (s, 1H), 6.48 (s, 1H), 5.80 (d, *J* = 3.0 Hz, 1H), 4.60-4.58 (m, 1H), 4.50-4.35 (m, 2H), 4.20-4.12 (m, 1H), 3.91-3.90 (m, 1H), 2.44 (s, 2H), 2.16 (s, 2H), 1.66-1.61 (m, 4H) ppm; ^{13}C NMR (50 MHz, DMSO-d_6): δ 145.7, 135.3, 128.0, 127.0, 126.6, 126.4, 125.4, 122.6, 120.9, 119.9, 110.0, 104.0, 68.7, 53.0, 47.2, 26.6, 24.9, 22.17, 21.5 ppm; HRMS (ESI) calcd for $C_{19}H_{21}N_4O$ [M + H] 321.1715 found 321.1714.

1-(4-Methoxyphenyl)-6,7-dihydro-5*H*-[1,2,3]triazolo[5',1':3,4][1,4]diazepino[1,2-*a*]indol-6-ol (6e):

Yield (0.138g, 66%); white solid; R_f 0.34 (1:1 ethyl acetate/hexanes); mp 190–192 °C; FT-IR (KBr) 3376, 2920, 1461, 1251, 739 cm^{-1}; ^1H NMR (300 MHz, DMSO-d_6): δ 7.83 (d, *J* = 9.0 Hz, 2H), 7.64 (t, *J* = 9.0 Hz, 2H), 7.26 (t, *J* = 9.0 Hz, 1H), 7.11 (t, *J* = 6.0 Hz, 1H), 7.01 (d, *J* = 9.0 Hz, 2H), 6.83 (s, 1H), 5.84 (d, *J* = 3.0 Hz, 1H), 4.64 (m, 1H), 4.58-4.45 (m, 2H), 4.27-4.21 (m, 1H), 4.01-3.95 (m, 1H), 3.79 (s, 3H) ppm; ^{13}C NMR (100 MHz, DMSO-d_6): δ 159.4, 143.7, 137.6, 128.1, 127.1, 126.3, 126.0, 123.0, 122.8, 121.0, 120.0, 114.1, 110.1, 103.5, 68.8, 55.2, 53.2, 47.4 ppm; HRMS (ESI) calcd for $C_{20}H_{19}N_4O_2$ [M + H] 347.1508 found 347.1497.

10,11-Dimethoxy-1-phenyl-6,7-dihydro-5*H*[1,2,3]triazolo[5',1':3,4][1,4]diazepino[1,2-*a*]indol-6-ol (6f):

Yield (0.142, 70%); white solid; R_f 0.22 (1:1 ethyl acetate/hexanes); mp 138–140 °C; FT-IR (KBr) 3239, 2934, 1595, 1483, 1223, 837 cm^{-1}; ^1H NMR (300 MHz, DMSO-d_6): δ7.92 (d, *J* = 6.0 Hz, 2H), 7.47-7.38 (m, 3H), 7.27 (s, 1H), 7.12 (s, 1H), 6.70 (s, 1H), 5.79 (d, *J* =3.0 Hz, 1H), 4.65-4.64 (m, 1H), 4.54-4.47 (m, 2H), 4.33-4.27 (m, 1H), 3.92-3.86 (m, 4H), 3.77 (s, 3H) ppm; ^{13}C NMR (50 MHz, DMSO-d_6): δ 148.0, 145.3, 142.9, 132.6, 130.8, 128.6, 128.1, 127.0, 126.5, 123.9, 119.6, 103.5, 102.6, 93.7, 68.8, 55.8, 53.4, 47.3 ppm; HRMS (ESI) calcd for $C_{21}H_{21}N_4O_3$ [M + H] 377.1613 found 377.1613.

10,11-Dimethoxy-1-(4-methylphenyl)-6,7-dihydro-5*H*-[1,2,3]triazolo[5',1':3,4][1,4]diazepino [1,2-*a*]indol-6-ol (6g):

Yield (0.138g, 69%); white solid; R_f 0.24 (1:1 ethyl acetate/hexanes); mp 146–148 °C; FT-IR (KBr) 2926, 2862, 1624, 1468, 1219, 825 cm^{-1}; ^{1}H NMR (300 MHz, DMSO-d_6): δ 7.77 (d, *J* = 6.0 Hz, 2H), 7.25-7.22 (m, 3H), 7.09 (s, 1H), 6.65 (s, 1H), 5.79 (d, *J* = 3.3 Hz, 1H), 4.61 (s, 1H), 4.53-4.42 (m, 2H), 4.30-4.25 (m, 1H), 3.83 (s, 4H), 3.74 (s, 3H), 2.32 (s, 3H) ppm; ^{13}C NMR (50 MHz, DMSO-d_6); δ 147.9, 145.3, 143.0, 137.5, 132.5, 129.1, 128.0, 126.7, 126.5, 124.0, 119.6, 103.3, 102.6, 93.7, 68.8, 55.8, 53.8, 47.3, 20.8 ppm; HRMS (ESI) calcd for $C_{22}H_{23}N_4O_3$ [M + H] 391.1770 found 391.1771.

10,11-Dimethoxy-1-(4-(*tert*-butyl)phenyl)-6,7-dihydro-5*H*-[1,2,3]triazolo[5',1':3,4][1,4]di-azepino[1,2-*a*]indol-6-ol (6h):

Yield (0.138g, 71%); white solid; R_f 0.26 (1:1 ethyl acetate/hexanes); mp 155–157 °C; FT-IR (KBr) 3239, 2956, 2350, 1624, 1483, 1221 cm^{-1}; ^{1}H NMR (300 MHz, DMSO-d_6): δ 7.84 (d, *J* = 9.0 Hz, 2H), 7.45 (d, *J* = 6.0 Hz, 2H), 7.26 (s, 1H), 7.11 (s, 1H), 6.72 (s, 1H), 5.78 (d, *J* = 3.0 Hz, 1H), 4.62-4.61 (m, 1H), 4.51-4.43 (m, 2H), 4.30-4.23 (m, 1H), 3.84 (s, 4H), 3.75 (s, 3H), 1.29 (s, 9H) ppm; ^{13}C NMR (75 MHz, DMSO-d_6); δ 155.9, 153.2, 150.5, 148.1, 137.8, 133.2, 132.0, 131.4, 130.6, 129.2, 124.8, 108.8, 107.8, 99.0, 74.1, 61.0, 58.6, 52.5,39.5, 36.2 ppm; HRMS (ESI) calcd for $C_{25}H_{29}N_4O_3$ [M + H] 433.2239 found 433.2239.

1-Phenyl-6,7-dihydro-5*H*-[1,3]dioxolo[4,5-*f*][1,2,3]triazolo[5',1':3,4][1,4]diazepino[1,2-*a*]indol-6-ol (6i):

Yield (0.138g, 67%); white solid; R_f 0.22 (1:1 ethyl acetate/hexanes); mp 202–204 °C; FT-IR (KBr) 3410, 2920, 1631, 1389, 1038 cm^{-1}; ^{1}H NMR (300 MHz, DMSO-d_6): δ 7.88 (d, *J* = 9 0 Hz, 2H), 7.46-7.36 (m, 3H), 7.27 (s, 1H), 7.06 (s, 1H), 6.69 (s, 1H), 5.98 (s, 2H), 5.78 (d, *J* = 3.0 Hz, 1H), 4.61-4.56 (m, 1H), 4.52-4.37 (m, 2H), 4.42-4.20 (m, 1H), 3.88-3.80 (m, 1H) ppm; ^{13}C NMR (75 MHz, DMSO-d_6): δ 145.6, 143.0, 133.3, 130.8, 128.7, 128.6, 126.7, 124.3, 120.1, 104.1, 100.6, 99.0, 91.1, 68.8, 53.3, 47.6 ppm; HRMS (ESI) calcd for $C_{20}H_{17}N_4O_3$ [M + H] 361.1300 found 361.1300.

1-(4-Methylphenyl)-6,7-dihydro-5*H*-[1,3]dioxolo[4,5-*f*][1,2,3]triazolo[5',1':3,4][1,4]diazepino[1,2-*a*]indol-6-ol (6j):

Yield (0.138g, 68%); white solid; R_f 0.23 (1:1 ethyl acetate/hexanes); mp 198–200 °C; FT-IR (KBr) 3219, 2920, 1479, 1228, 833 cm^{-1}; ^1H NMR (300 MHz, DMSO-d_6): δ 7.75 (d, *J* = 9 0 Hz, 2H), 7.26-7.25 (m, 2H), 7.22 (s, 1H), 7.05 (s, 1H), 6.65 (s, 1H), 5.98 (s, 2H), 5.75 (d, *J* = 3.0 Hz, 1H), 4.60-4.59 (m, 1H), 4.51-4.36 (m, 2H), 4.24-4.17 (m, 1H), 3.90-3.83 (m, 1H), 2.32 (s, 3H) ppm; ^{13}C NMR (50 MHz, DMSO-d_6): δ 145.9, 143.2, 137.9, 133.5, 129.4, 128.0, 126.7, 124.4, 121.2, 104.4, 100.8, 99.2, 91.2, 68.8, 53.2, 47.7, 20.8 ppm; HRMS (ESI) calcd for $C_{21}H_{19}N_4O_3$ [M + H] 375.1457 found 375.1458.

1-(4-(*tert*-Butyl)phenyl)-6,7-dihydro-5*H*[1,3]dioxolo[4,5-*f*][1,2,3]triazolo[5',1':3,4][1,4]diazepino[1,2-*a*]indol-6-ol (6k):

Yield (0.130g, 66%); white solid; R_f 0.26 (1:1 ethyl acetate/hexanes); mp 206–208 °C; FT-IR (KBr) 3333, 2954, 1617, 1471, 1216, 839 cm^{-1}; ^1H NMR (300 MHz, $CDCl_3$): δ 7.87 (d, *J* = 9 0 Hz, 2H), 7.40 (d, *J* = 9.0 Hz, 2H), 6.98 (s, 1H), 6.90 (s, 1H), 6.75 (s, 1H), 5.96 (s, 2H), 4.74 (s, 1H), 4.66-4.60 (m, 1H), 4.40-4.26 (m, 2H), 4.09-4.02 (m, 1H), 3.79 (s, 1H), 1.33 (s, 9H) ppm; ^{13}C NMR (50 MHz, $CDCl_3$): δ 151.7, 146.4, 144.2, 143.8, 133.6, 127.5, 127.2, 126.6, 125.7, 124.6, 121.6, 105.1, 101.0, 99.5, 90.4, 70.2, 53.4, 48.3, 34.8, 31.3 ppm; HRMS (ESI) calcd for $C_{24}H_{25}N_4O_3$ [M + H] 417.1926 found 417.1929.

1-(4-Methoxyphenyl)-6,7-dihydro-5*H*-[1,3]dioxolo[4,5-*f*][1,2,3]triazolo[5',1':3,4][1,4]diazepino[1,2-*a*]indol-6-ol (6l):

Yield (0.132g, 69%); white solid; R_f 0.24 (1:1 ethyl acetate/hexanes); mp 152–154 °C; FT-IR (KBr) 3413, 2926, 1635, 1461 cm^{-1}; ^1H NMR (300 MHz, $CDCl_3$): δ 7.81 (d, *J* = 9.0 Hz, 2H), 7.26 (s, 1H), 7.06 (s, 1H), 7.00 (d, *J* = 6.0 Hz, 2H), 6.67 (s, 1H), 5.98 (s, 2H), 5.76 (d, *J* = 6.0 Hz, 1H), 4.61-4.59 (m, 1H), 4.51-4.36 (m, 2H), 4.24-4.18 (m, 1H), 3.91-3.84 (m, 1H), 3.79 (s, 3H) ppm; ^{13}C NMR (50 MHz, DMSO-d_6): δ 159.2, 145.5, 143.0, 133.2, 128.0, 126.1, 124.5, 123.1, 120.8, 114.1, 103.8, 100.6, 98.9, 91.2, 68.7, 55.1, 53.2, 47.5 ppm; HRMS (ESI) calcd for $C_{21}H_{19}N_4O_4$ [M + H] 391.1406 found 391.1406.

9-Methoxy-1-phenyl-6,7-dihydro-5*H*-[1,2,3]triazolo[5',1':3,4][1,4]diazepino[1,2-*a*]indol-6-ol (6m):

Yield (0.140g, 67%); white solid; R_f 0.26 (1:1 ethyl acetate/hexanes); mp 210–212 °C; FT-IR (KBr) 3350, 2942, 1579, 1366, 1261 cm^{-1}; ^{1}H NMR (300 MHz, $CDCl_3$): δ 7.90 (d, *J* = 6.0 Hz, 2H), 7.36-7.26 (m, 3H), 7.24 (t, *J* = 9.0 Hz, 1H), 7.07 (t, *J* = 6.0 Hz, 1H), 6.75 (t, *J* = 6.0 Hz, 2H), 5.00-4.94 (m, 1H), 4.76-4.75 (m, 1H), 4.64-4.58 (m, 1H), 4.48-4.34 (m, 2H), 4.01 (s, 3H), 3.59 (d, *J* = 6.0 Hz, 1H) ppm; ^{13}C NMR (100 MHz, DMSO-d_6): δ 146.9, 143.7, 130.6, 129.0, 128.6, 128.3, 126.8, 126.4, 124.6, 113.8, 104.3, 69.6, 55.7, 53.5, 48.8 ppm; HRMS (ESI) calcd for $C_{20}H_{19}N_4O_2$ [M + H] 347.1508 found 347.1509.

9-Methoxy-1-(4-methylphenyl)-6,7-dihydro-5*H*[1,2,3]triazolo[5',1':3,4][1,4]diazepino[1,2-*a*]indol-6-ol (6n):

Yield (0.146g, 71%); white solid; R_f 0.29 (1:1 ethyl acetate/hexanes); mp 228–230 °C; FT-IR (KBr) 3296, 2927, 1572, 1436, 1252, 823 cm^{-1}; ^{1}H NMR (300 MHz, $CDCl_3$): δ 7.81 (d, *J* = 9.0 Hz, 2H), 7.26 (s, 1H), 7.25-7.17 (m, 2H), 7.08 (t, *J* = 9.0 Hz, 1H), 6.78-6.74 (m, 2H), 4.94-4.87 (m, 1H), 4.76-4.74 (m, 1H), 4.69-4.62 (m, 1H), 4.55-4.48 (m, 1H), 4.43-4.37 (m, 1H), 4.02 (s, 3H), 3.26 (d, *J* = 9.0 Hz, 1H), 2.38 (s, 3H) ppm; ^{13}C NMR (50 MHz, DMSO-d_6): δ 146.9, 143.8, 137.7, 129.2, 127.7, 126.8, 126.8, 126.6, 126.1, 120.6, 113.8, 104.3, 104.2, 69.5, 55.7, 53.4, 48.8, 20.8 ppm; HRMS (ESI) calcd for $C_{21}H_{21}N_4O_2$ [M + H] 361.1665 found 361.1667.

9-Methoxy-1-(4-(*tert*-butyl)phenyl)-6,7-dihydro-5*H*-[1,2,3]triazolo[5',1':3,4][1,4]di-azepino[1,2-*a*]indol-6-ol (6o):

Yield (0.139g, 70%); white solid; R_f 0.31 (1:1 ethyl acetate/hexanes); mp 234–236 °C; FT-IR (KBr) 3353, 2951, 1564, 1358, 1223, 725 cm^{-1}; ^{1}H NMR (300 MHz, $CDCl_3$): δ 7.88 (d, *J* = 6.0 Hz, 2H), 7.39 (d, *J* = 9.0 Hz, 2H), 7.25 (s, 1H), 7.07 (t, *J* = 9.0 Hz, 1H), 6.82 (s, 1H), 6.73 (d, *J* = 9.0 Hz, 1H), 4.90-4.84 (m, 1H), 4.74-4.73 (m, 1H), 4.66-4.60 (m, 1H), 4.48-4.37 (m, 2H), 4.00 (s, 3H), 3.39 (d, *J* = 6.0 Hz, 1H), 1.32 (s, 9H) ppm; ^{13}C NMR (75 MHz, DMSO-d_6): δ 150.8, 146.9, 143.7, 128.9, 127.8, 126.8, 126.3, 126.1, 125.4, 120.6, 113.8, 104.4, 104.3, 69.7, 55.7, 53.4, 48.8, 34.4, 31.0 ppm; HRMS (ESI) calcd for $C_{24}H_{27}N_4O_2$ [M + H] 403.2134 found 403.2135.

11-Chloro-1-(4-methylphenyl)-6,7dihydro-5*H*-[1,2,3]triazolo[5',1':3,4][1,4]diazepino[1,2-*a*]indol-6-ol (6p):

Yield (0.146g, 71%); white solid; R_f 0.27 (1:1 ethyl acetate/hexanes); mp 224–226 °C; FT-IR (KBr) 3405, 2925, 2862, 1732, 1458, 793 cm^{-1}; ^1H NMR (300 MHz, DMSO-d_6): δ 7.74 (d, *J* = 7.8 Hz, 2H), 7.68 (d, *J* = 9.0 Hz, 2H), 7.25-7.23 (m, 3H), 6.77 (s, 1H), 5.84 (d, *J* = 6.0 Hz, 1H), 4.65-4.64 (m, 1H), 4.60-4.44 (m, 2H), 4.26-4.20 (m, 1H), 4.02-3.96 (m, 1H), 2.32 (s, 3H) ppm; ^{13}C NMR (50 MHz, DMSO-d_6): δ 144.1, 138.0, 136.2, 129.3, 128.0, 127.6, 126.8, 126.0, 124.6, 122.8, 120.1, 111.9, 103.1, 68.7, 53.2, 47.7, 20.9.ppm; HRMS (ESI) calcd for $C_{20}H_{18}ClN_4O$ [M + H] 365.1169 found 365.1179.

11-Chloro-1-phenyl-6,7-dihydro-5*H*-[1,2,3]triazolo[5',1':3,4][1,4]diazepino[1,2-*a*]indol-6-ol (6q):

Yield (0.150g, 72%); white solid; R_f 0.24 (1:1 ethyl acetate/hexanes); mp 230–232 °C; FT-IR (KBr) 3433, 2925, 1502, 525 cm^{-1}; ^1H NMR (300 MHz, DMSO-d_6): δ 7.87 (d, *J* = 9 0 Hz, 2H), 7.70 (s, 2H), 7.46-7.38 (m, 3H), 7.25 (d, *J* = 9.0 Hz, 1H), 6.85 (s, 1H), 5.84 (d, *J* = 6.0 Hz, 1H), 4.66-4.64 (m, 1H), 4.60-4.45 (m, 2H), 4.27-4.20 (m, 1H), 4.03-3.96 (m, 1H) ppm; ^{13}C NMR (50 MHz, DMSO-d_6): δ 144.1, 136.2, 130.5, 128.8, 128.5, 128.1, 127.7, 126.8, 124.6, 122.8, 120.1, 111.9, 103.2, 68.7, 53.3, 47.7 ppm; HRMS (ESI) calcd for $C_{19}H_{16}ClN_4O$ [M + H] 351.1013 found 351.1008.

1-Butyl-6,7-dihydro-5*H*-[1,2,3]triazolo[5',1':3,4][1,4]diazepino[1,2-*a*]indol-6-ol (6r):

Yield (0.142g, 63%); white solid; R_f 0.32 (1:1 ethyl acetate/hexanes); mp 128–130 °C; FT-IR (KBr) 3303, 2934, 1451, 1248, 747 cm^{-1}; ^1H NMR (300 MHz, DMSO-d_6): δ 7.64 (t, *J* = 9.0 Hz, 2H), 7.25 (t, *J* = 6.0 Hz, 1H), 7.11 (t, *J* = 6.0 Hz, 1H), 6.84 (s, 1H), 5.81 (d, *J* = 3.0 Hz, 1H), 4.62-4.56 (m, 2H), 4.38-4.26 (m, 2H), 3.93-3.86 (m, 1H), 2.80 (t, *J* = 6.0 Hz, 2H), 1.68 (t, *J* = 6.0 Hz, 2H), 1.39-1.32 (m, 2H), 0.89 (t, *J* = 6.0 Hz, 3H) ppm; ^{13}C NMR (100 MHz, DMSO-d_6): δ 145.2, 138.0, 127.4, 127.1, 126.8, 122.7, 120.9, 120.1, 110.1, 102.4, 68.4, 53.3, 47.9, 30.9, 24.5, 21.8, 13.7 ppm; HRMS (ESI) calcd for $C_{17}H_{21}N_4O$ [M + H] 297.1715 found 297.1725.

1-Butyl-6,7-dihydro-5*H*-[1,3]dioxolo[4,5-*f*][1,2,3]triazolo[5',1':3,4][1,4]diazepino[1,2-*a*]indol-6-ol (6s):

Yield (0.138g, 61%); white solid; R_f 0.25 (1:1 ethyl acetate/hexanes); mp 150–152 °C; FT-IR (KBr) 3327, 2925, 1624, 1471, 1217 cm^{-1}; ^1H NMR (300 MHz, $CDCl_3$): δ 7.24 (s, 1H), 7.09 (s, 1H), 6.67 (s, 1H), 5.98 (s, 2H), 5.73 (d, *J* = 3.0 Hz, 1H), 4.60-4.50 (m, 2H), 4.30-4.23 (m, 2H), 3.83-3.76 (m, 1H), 2.78-2.73 (m, 2H), 1.69-1.62 (m, 2H), 1.36-1.30 (m, 2H) 0.85-0.80 (m, 3H) ppm; ^{13}C NMR (75 MHz, DMSO-d_6): δ 145.3, 144.4, 142.9, 133.5, 127.2, 125.1, 121.2, 102.7, 100.5, 98.9, 91.2, 68.3, 53.2, 48.1, 31.0, 24.4, 21.7, 13.7 ppm; HRMS (ESI) calcd for $C_{18}H_{21}N_4O_3$ [M + H] 341.1613 found 341.1621.

1-Hexyl-6,7-dihydro-5*H*-[1,3]dioxolo[4,5-*f*][1,2,3]triazolo[5',1':3,4][1,4]diazepino[1,2-*a*]indol-6-ol (6t):

Yield (0.129g, 63%); white solid; R_f 0.28 (1:1 ethyl acetate/hexanes); mp 144–146 °C; FT-IR (KBr) 3239, 2928, 1620, 1468, 1216 cm^{-1}; ^1H NMR (300 MHz, $CDCl_3$): δ 7.24(s, 1H), 7.09 (s, 1H), 6.67 (s, 1H), 5.98 (s, 2H), 5.73 (d, *J* = 3.0 Hz, 1H), 4.60-4.50 (m, 2H), 4.30-4.23 (m, 2H), 3.83-3.78 (m, 1H), 2.78-2.73 (m, 3H), 1.70-1.65 (m, 2H), 1.32-1.26 (m, 6H), 0.85-0.80 (m, 3H) ppm; ^{13}C NMR (75 MHz, DMSO-d_6): δ 145.3, 144.4, 142.9, 133.5, 127.3, 125.1, 121.2, 102.7, 100.6, 98.9, 68.4, 53.3, 48.1, 31.0, 28.7, 28.3, 24.7, 22.0, 13.8 ppm; HRMS (ESI) calcd for $C_{20}H_{25}N_4O_3$ [M + H] 369.1926 found 369.1927.

6,7-Dihydro-5*H*-[1,2,3]triazolo[5',1':3,4][1,4]diazepino[1,2-*a*]indol-6-ol (6u):

Yield (0.096g, 57%); white solid; R_f 0.17 (1:1 ethyl acetate/hexanes); mp 188–190 °C; FT-IR (KBr) 3353, 2951, 1564, 1358, 1223, 725 cm^{-1}; ^1H NMR (300 MHz, DMSO-d_6): δ 8.21 (s, 1H), 7.63-7.56 (m, 2H), 7.27-7.22 (m, 1H), 7.14-7.09 (m, 1H), 7.04 (s, 1H), 5.76 (s, 1H), 4.76-4.64 (m, 3H), 4.43-4.38 (m, 2H) ppm; ^{13}C NMR (75 MHz, DMSO-d_6): δ 138.3, 132.4, 131.0, 127.3, 126.4, 122.6, 120.6, 120.2, 110.2, 102.6, 65.1, 54.4, 49.3 ppm; HRMS (ESI) calcd for $C_{13}H_{13}N_4O$ [M + H] 241.1089 found 241.1097.

10,11-Dimethoxy-6,7-dihydro-5*H*-[1,2,3]triazolo[5',1':3,4][1,4]diazepino[1,2-*a*]indol-6-ol (6v):

Yield (0.089g, 54%); white solid; R_f 0.14 (1:1 ethyl acetate/hexanes); mp 200–202 °C; FT-IR (KBr) 3218, 2934, 1597, 1479, 1226, 834 cm^{-1}; ^1H NMR (300 MHz, DMSO-d_6): δ 8.09 (s, 1H), 7.16 (s, 1H), 7.09 (s, 1H), 6.86 (s, 1H), 5.71 (d, *J* = 6.0 Hz, 1H), 4.65-4.60 (m, 3H), 4.42-4.36 (m, 1H), 4.28-4.21 (m, 1H), 3.85 (s, 3H), 3.78 (s, 3H) ppm; ^{13}C NMR (50 MHz, DMSO-d_6): δ 147.7, 145.4, 133.2, 131.5, 124.4, 120.0, 102.4, 94.0, 65.3, 55.8, 54.4, 49.5 ppm; HRMS (ESI) calcd for $C_{15}H_{17}N_4O_3$ [M + H] 301.1301 found 301.1328.

^1H NMR and ^{13}C NMR spectra

For copies of ^1H and ^{13}C NMR spectra of componds **1a–1d**, **1f**, **1g**, **1i**, **1m**, **1u –** see reference [1].

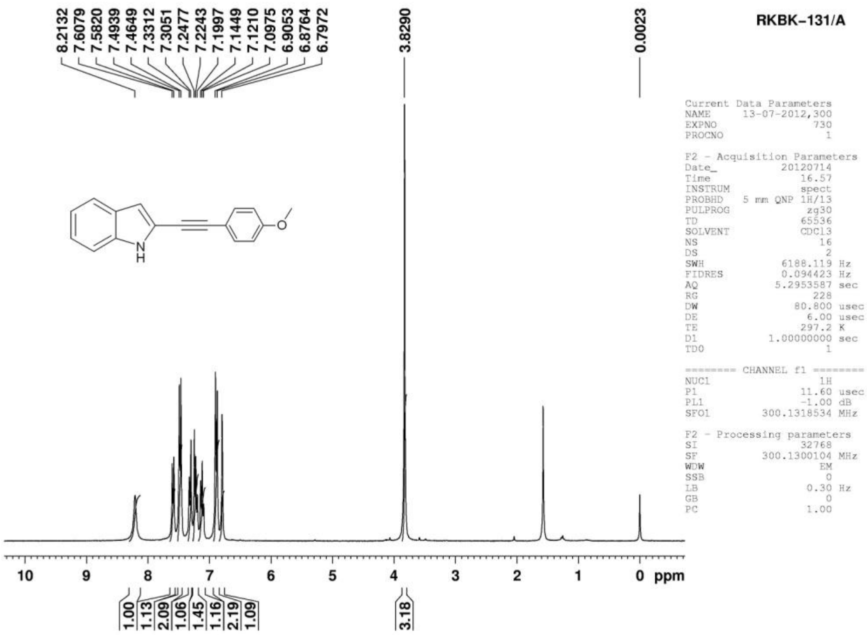

Figure 1: ^{1}H NMR of 1e

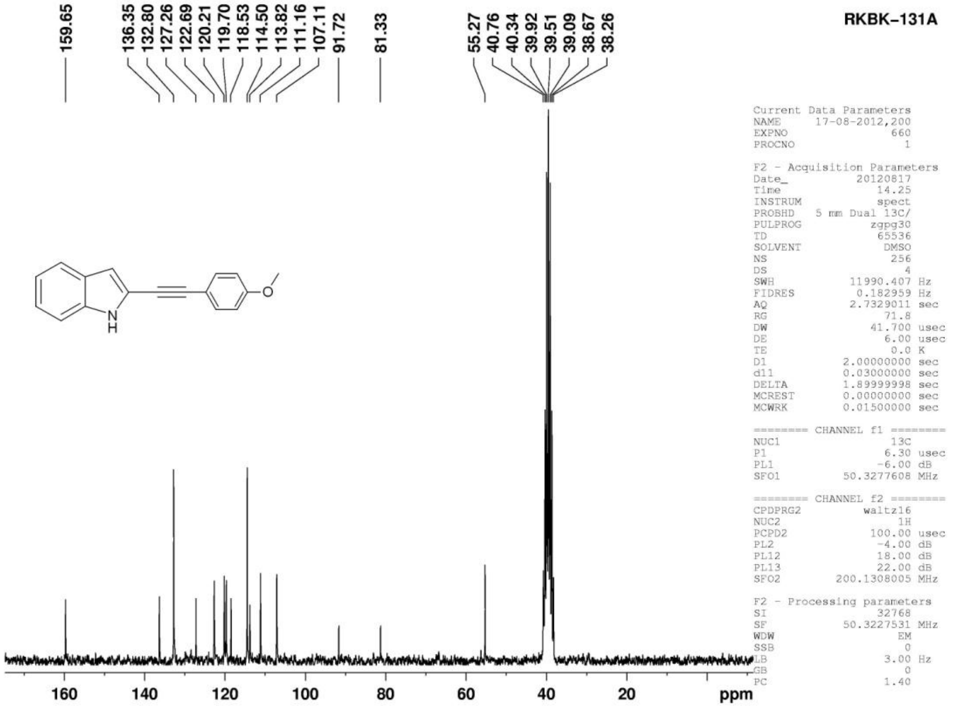

Figure 2: ^{13}C NMR of 1e

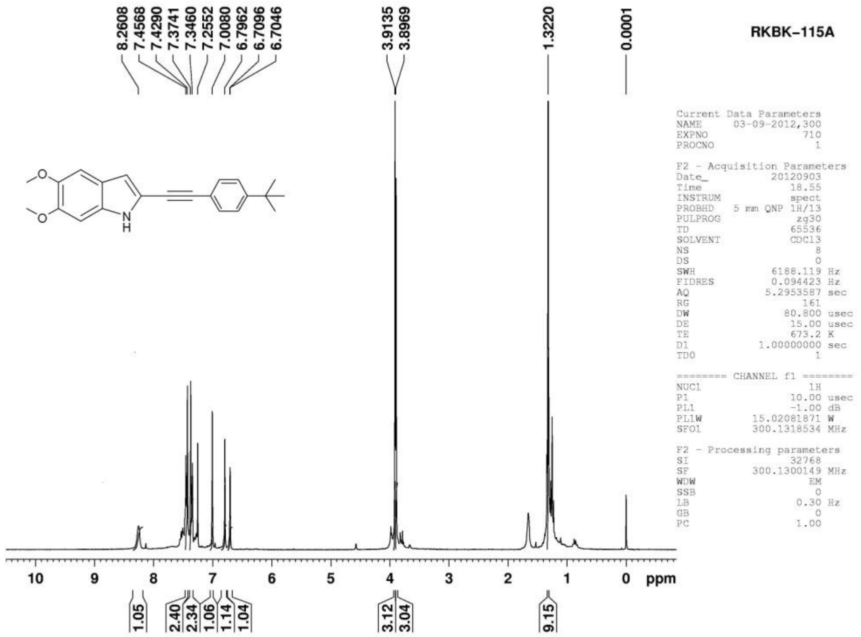

Figure 3: ^{1}H NMR of 1h

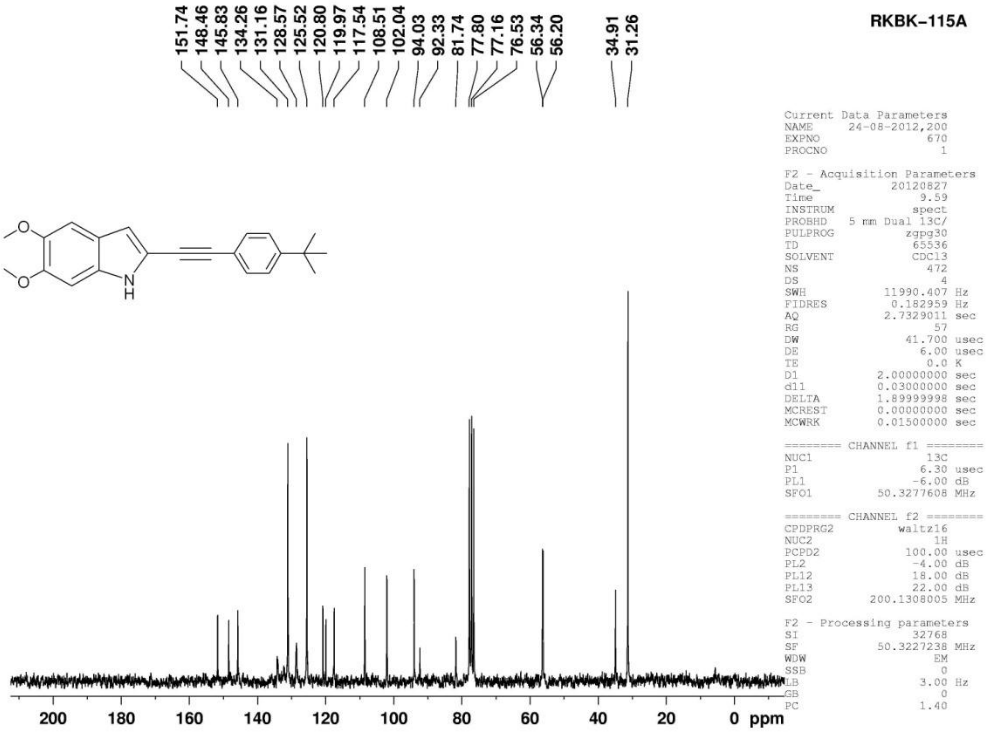

Figure 4: ^{13}C NMR of 1h

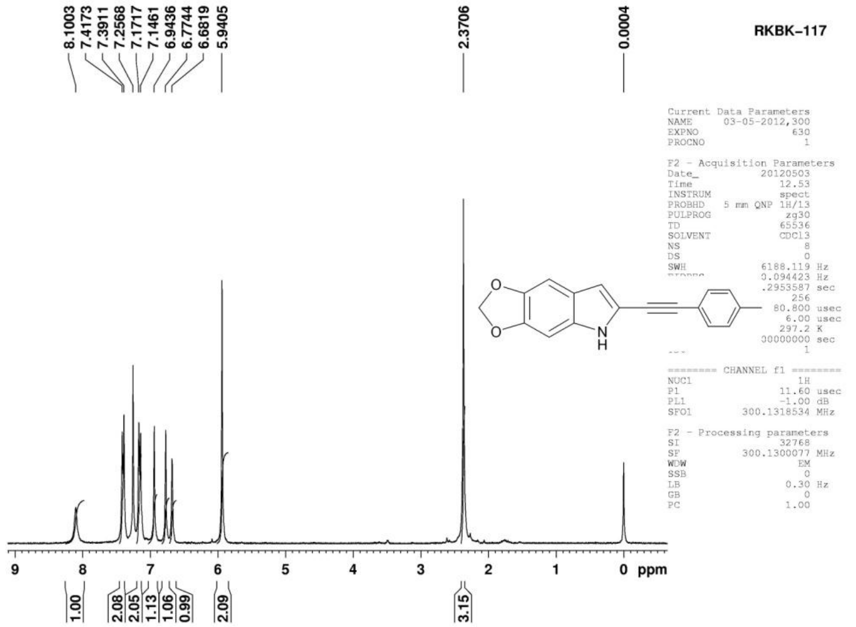

Figure 5: ^{1}H NMR of 1j

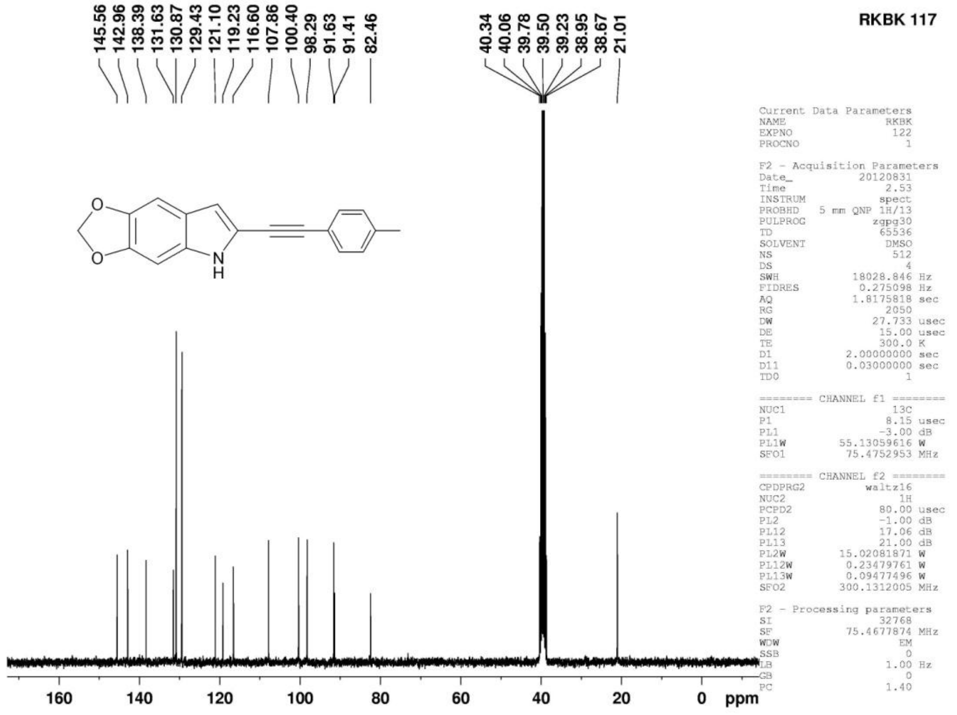

Figure 6: ^{13}C NMR of 1j

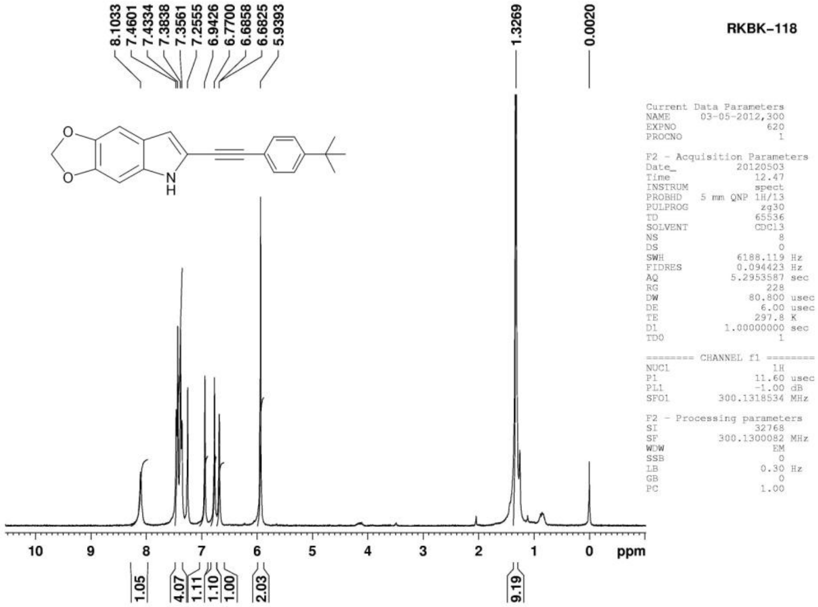

Figure 7: ^{1}H NMR of 1k

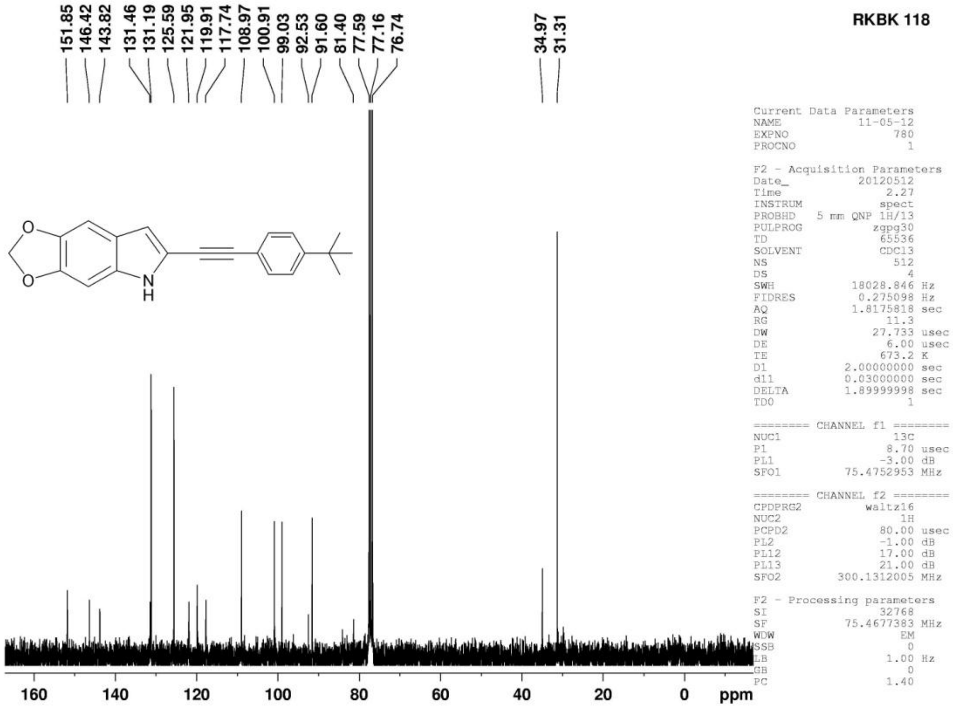

Figure 8: ^{13}C NMR of 1k

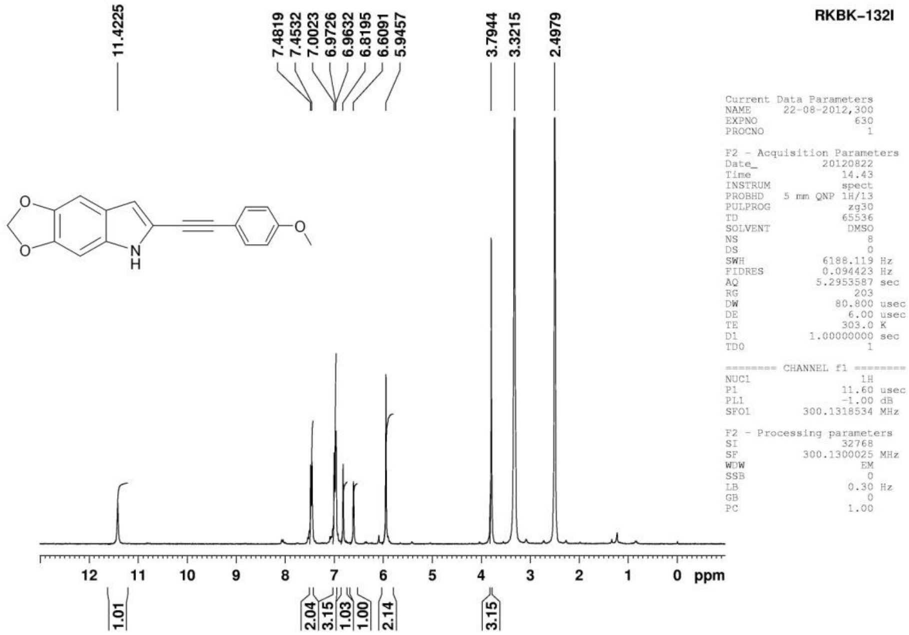

Figure 9: ^{1}H NMR of 1l

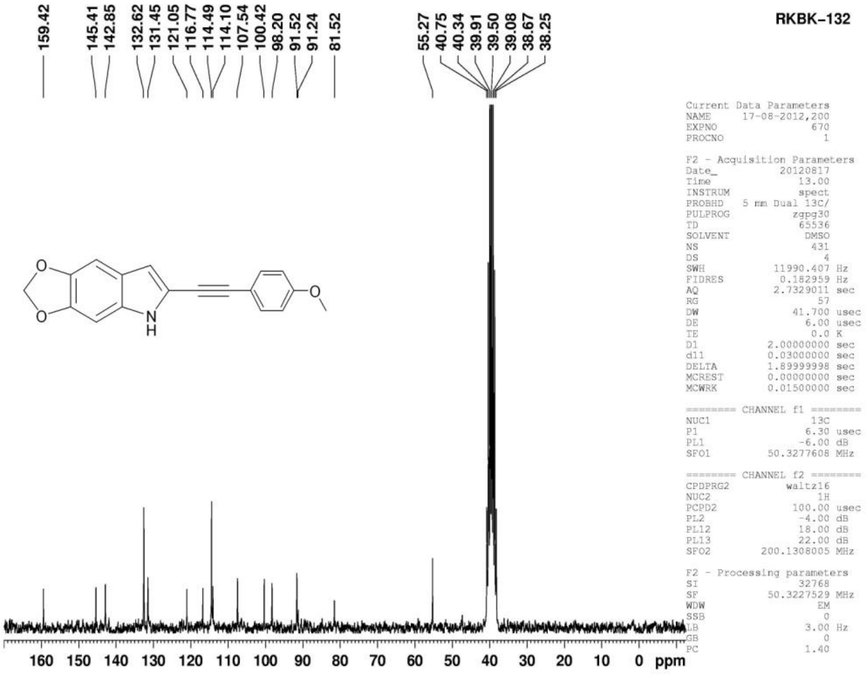

Figure 10: ^{13}C NMR of 1l

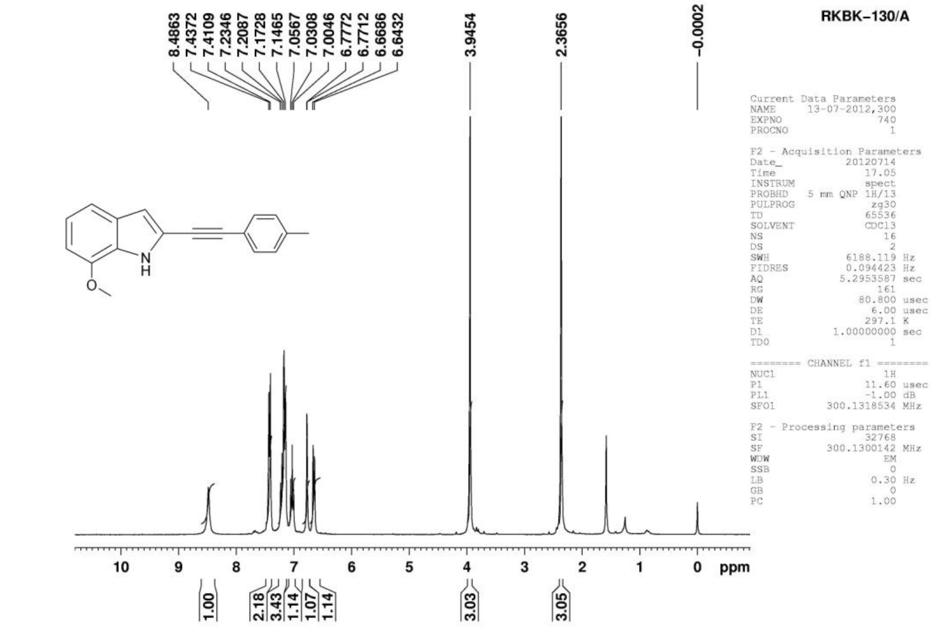

Figure 11: ^{1}H NMR of 1n

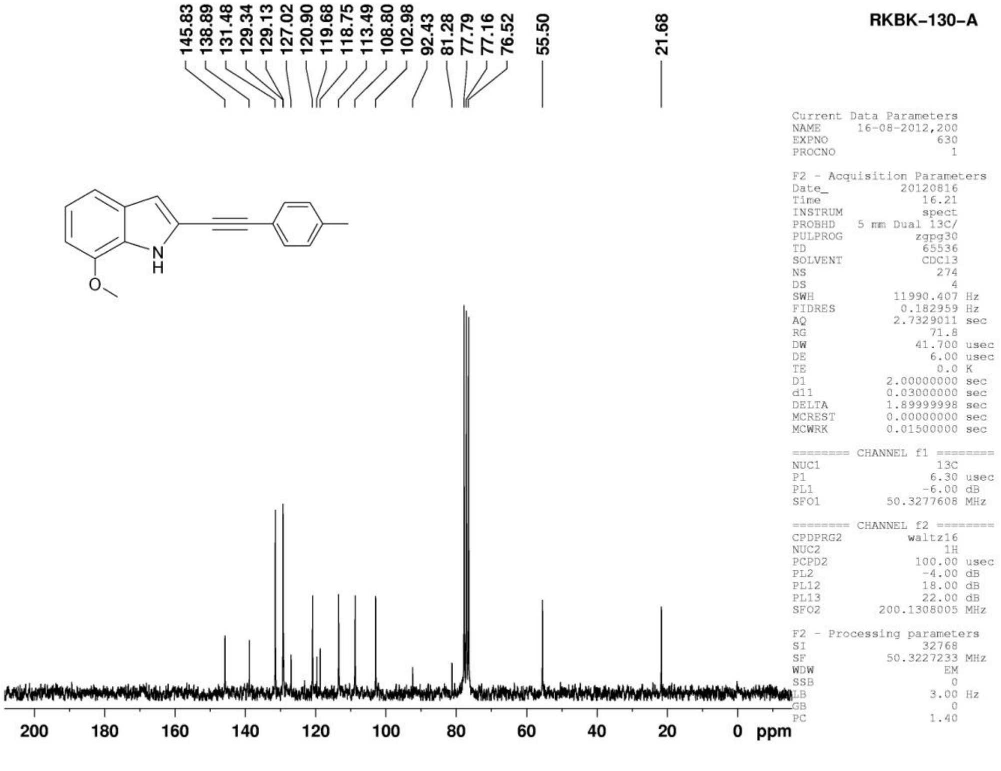

Figure 12: ^{13}C NMR of 1n

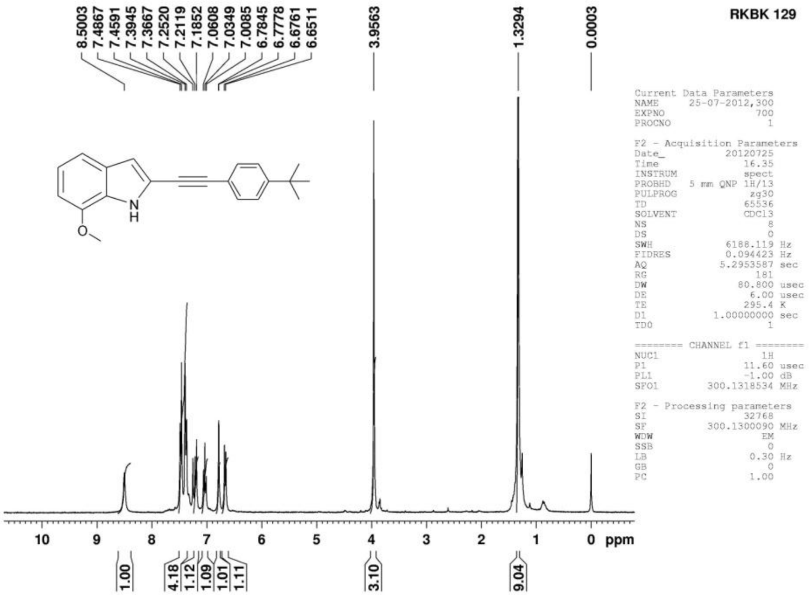

Figure 13: ^{1}H NMR of 1o

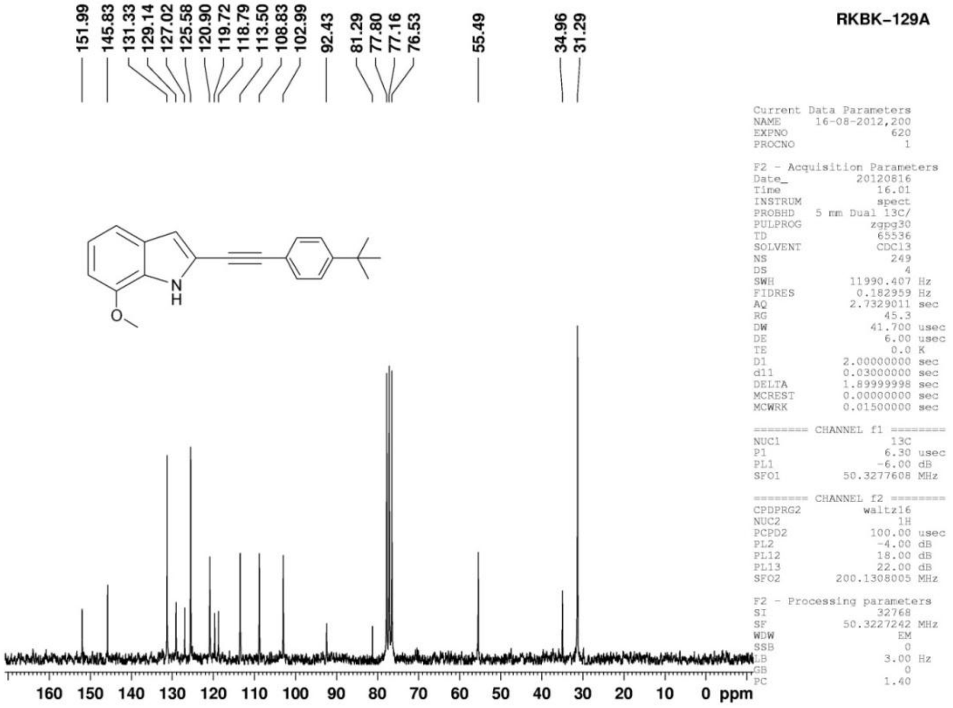

Figure 14: ^{13}C NMR of 1o

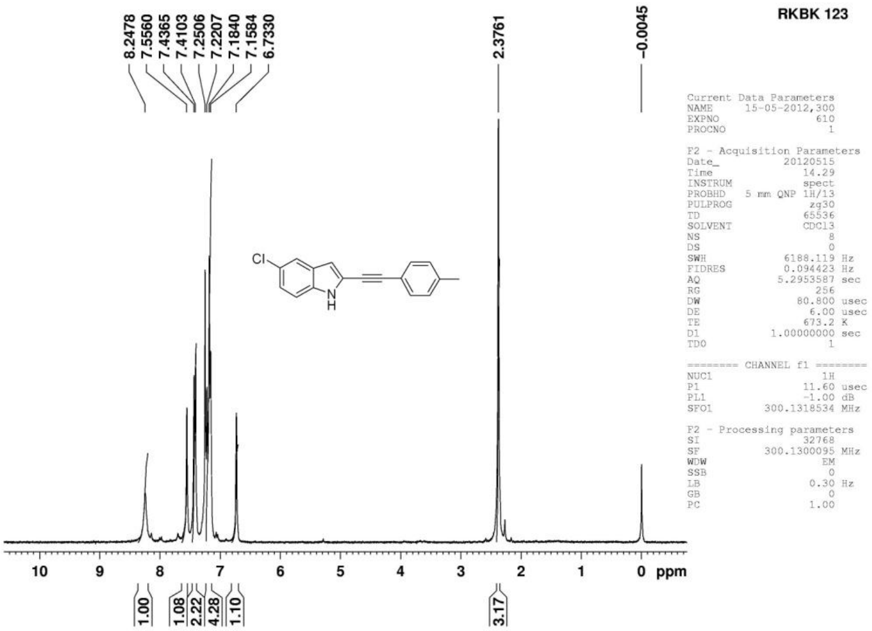

Figure 15: ^{1}H NMR of 1p

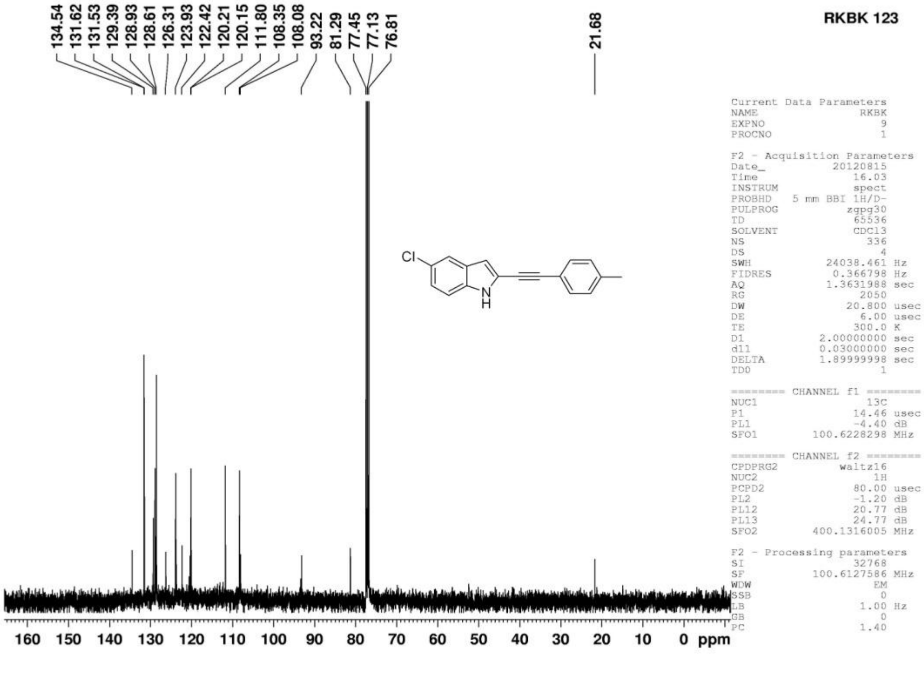

Figure 16: ^{13}C NMR of 1p

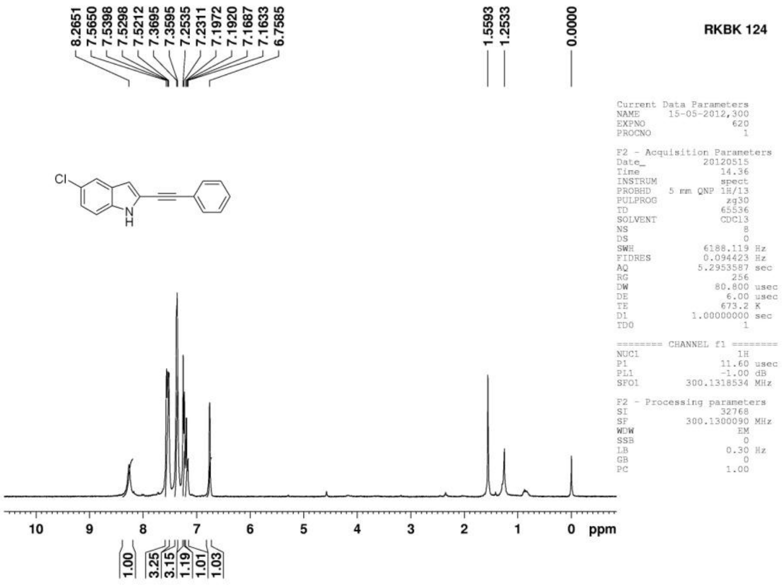

Figure 17: ^{1}H NMR of 1q

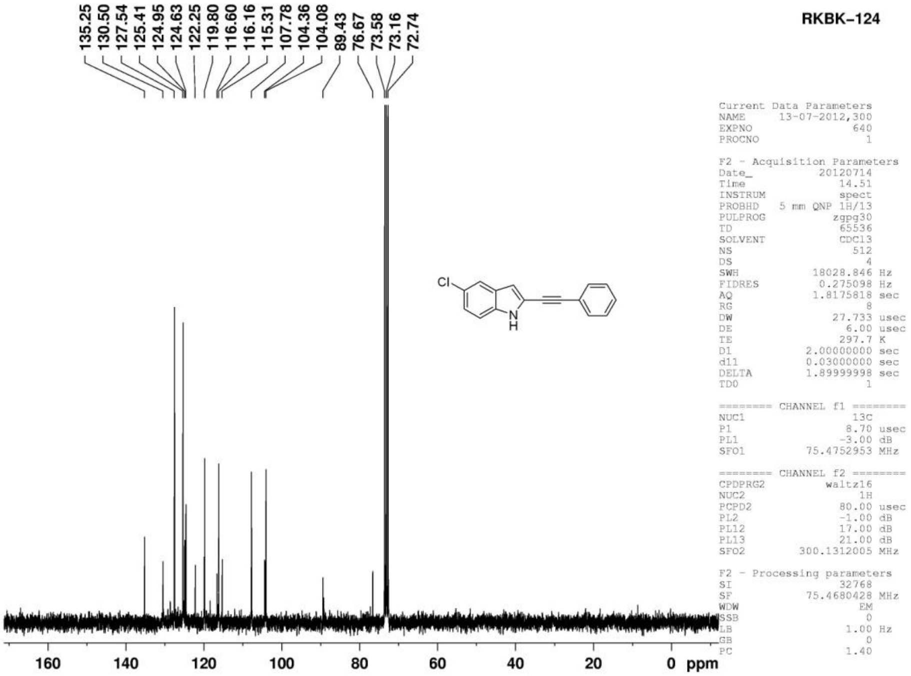

Figure 18:^{13}C NMR of 1q

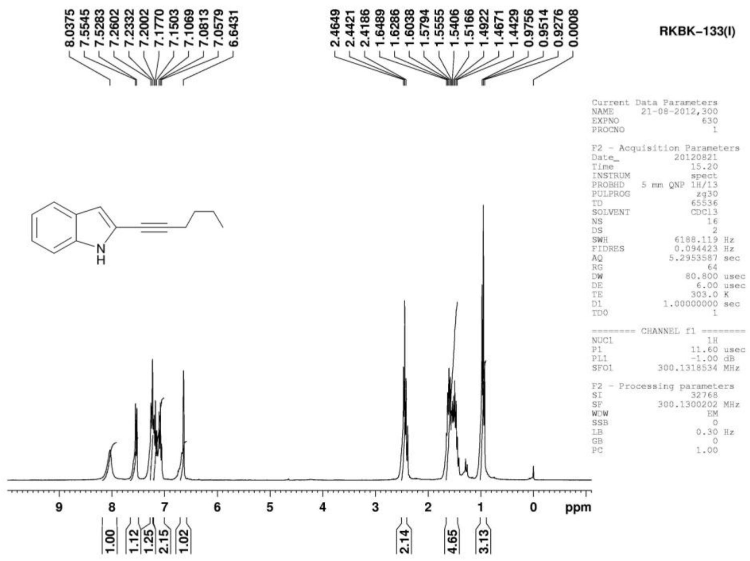

Figure 19:^{1}**H NMR of 1r**

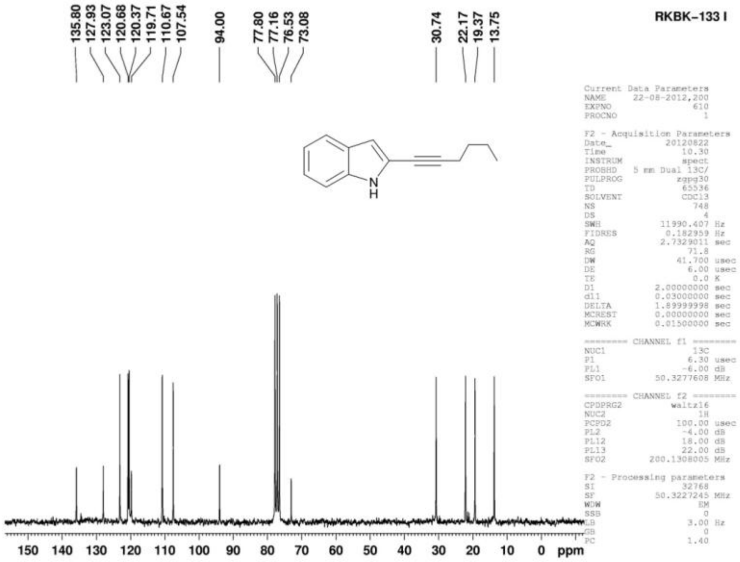

Figure 20: ^{13}**C NMR of 1r**

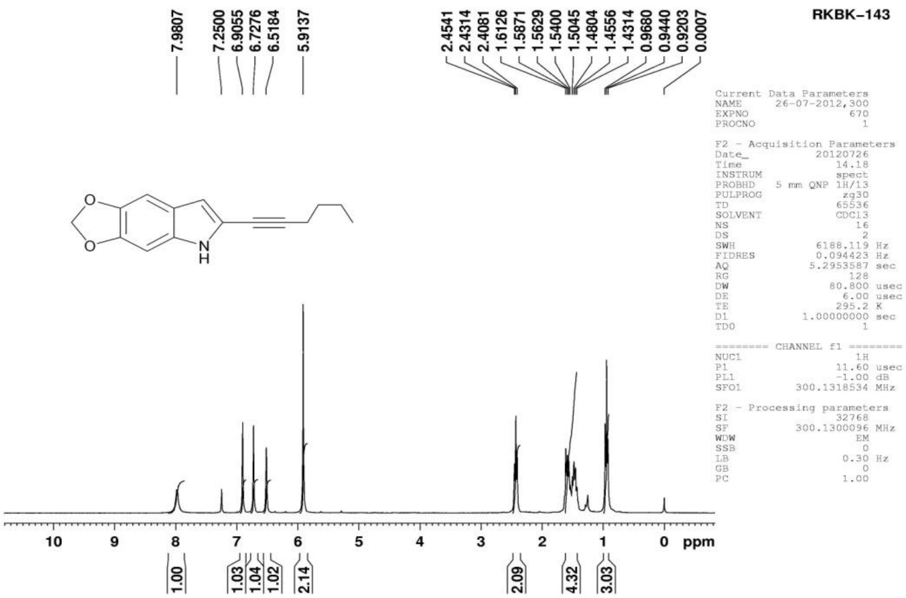

Figure 21: ^{1}H NMR of 1s

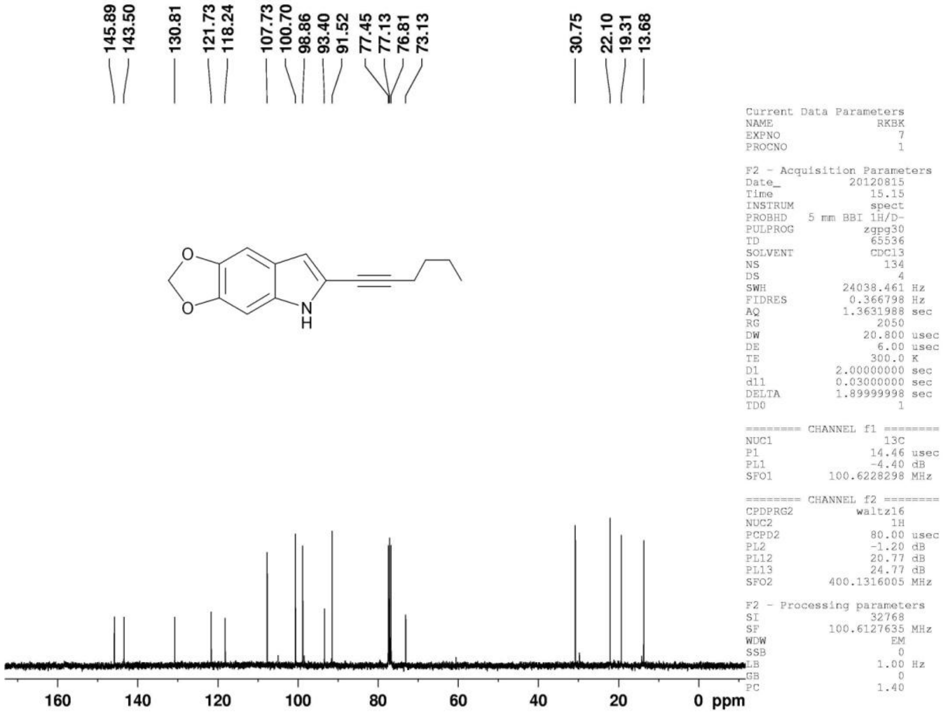

Figure 22: ^{13}C NMR of 1s

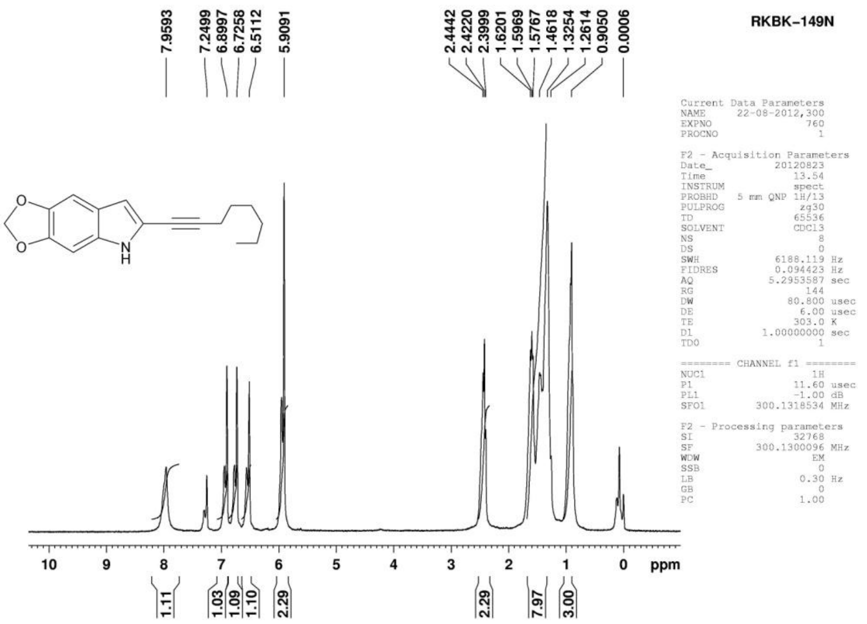

Figure 23: ^{1}H NMR of 1t

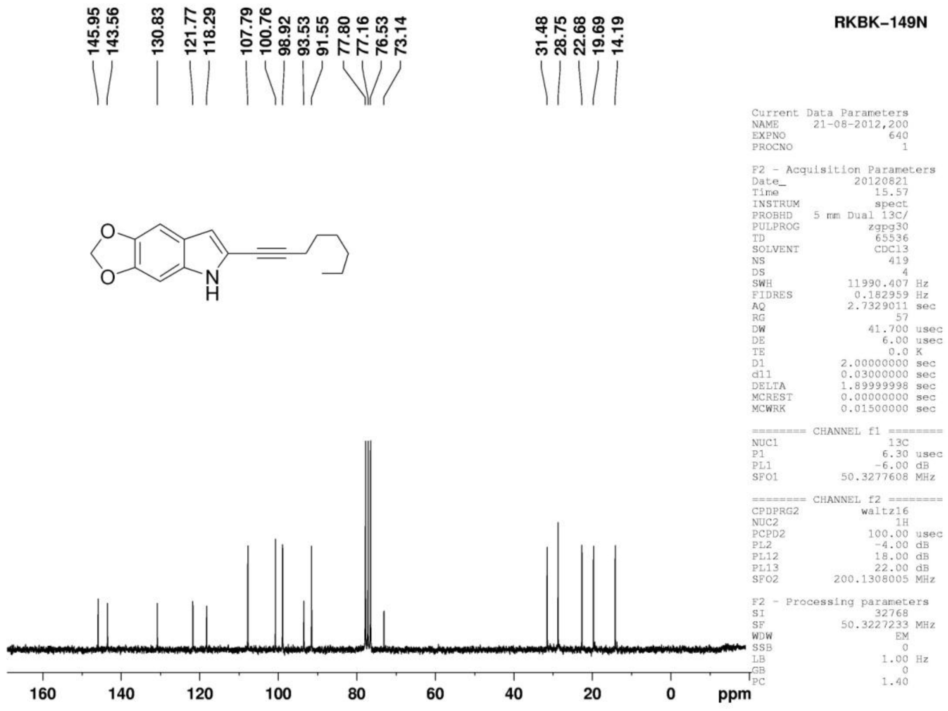

Figure 24: ^{13}C NMR of 1t

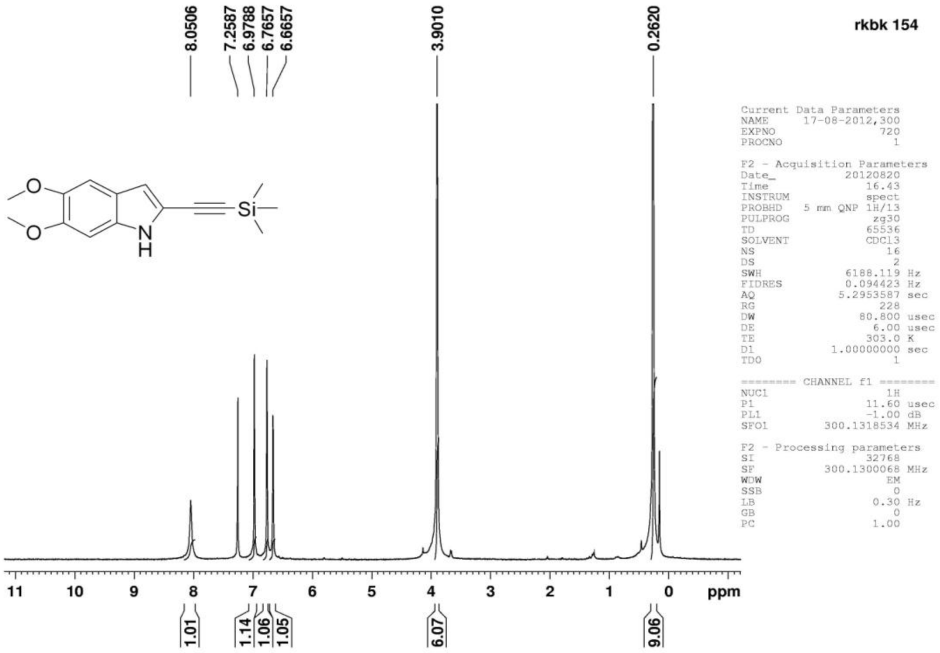

Figure 25: ^{1}H NMR of 1v

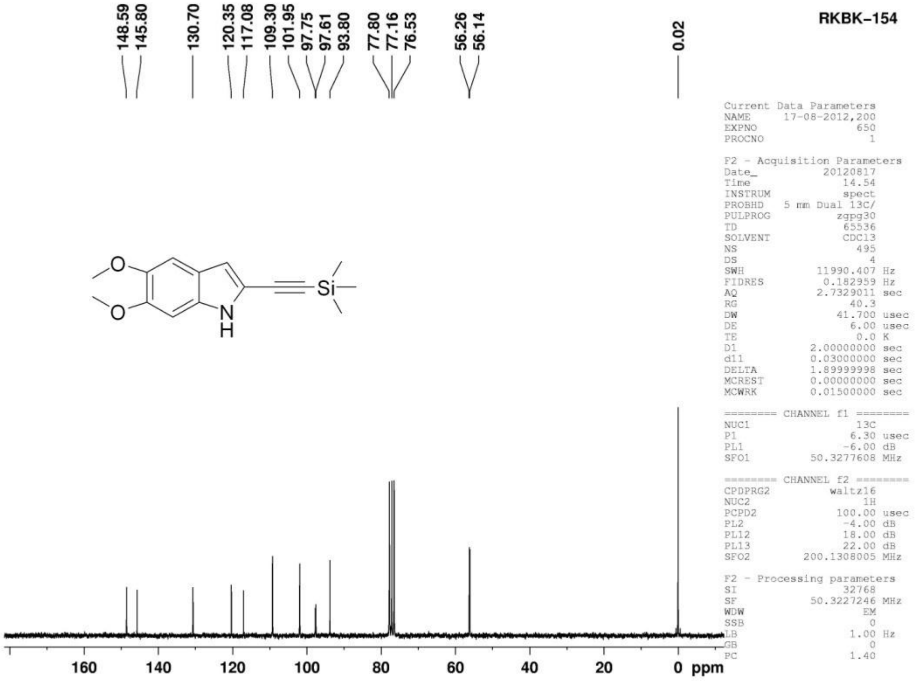

Figure 26: ^{13}C NMR of 1v

^{1}H & ^{13}C Spectra of intermediate 4a and 5a

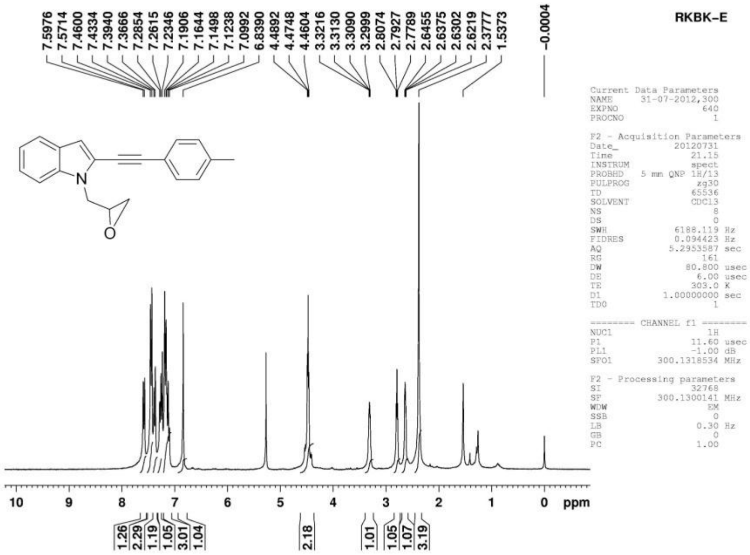

Figure 27: 1H NMR of 4a

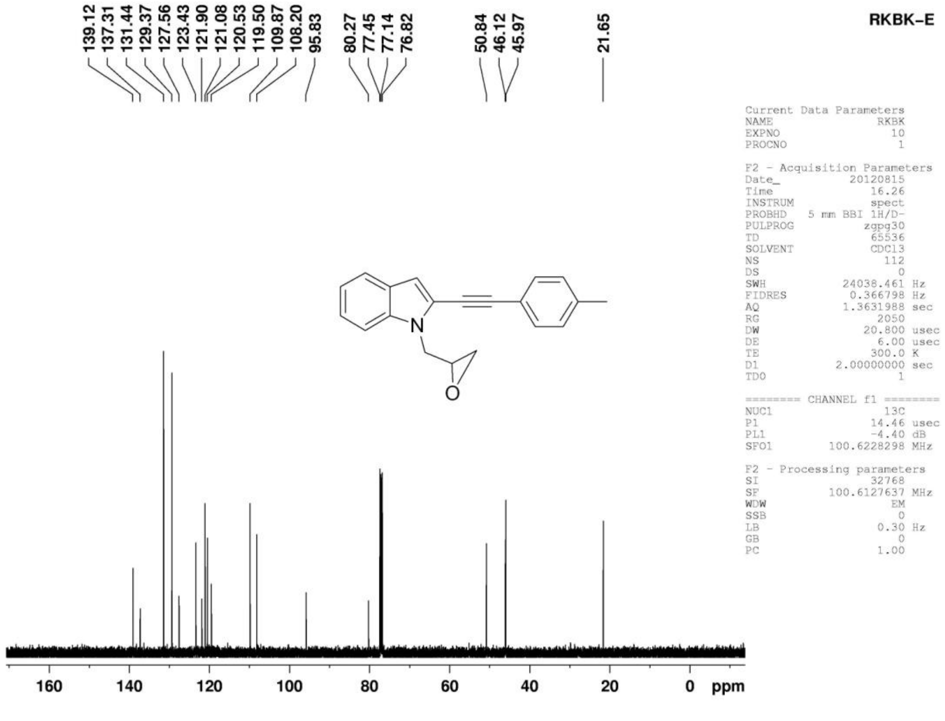

Figure 28: ^{13}C NMR of 4a

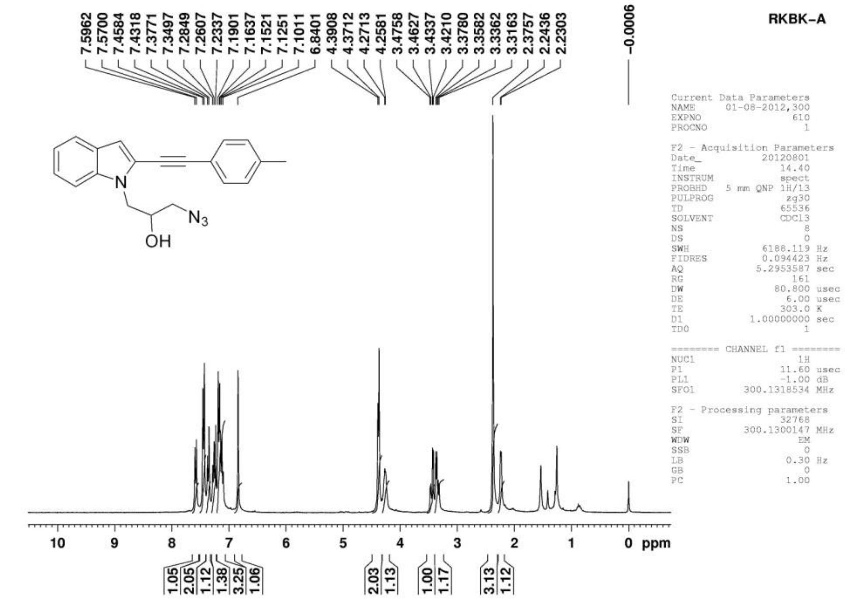

Figure 29: ^{1}H NMR of 5a

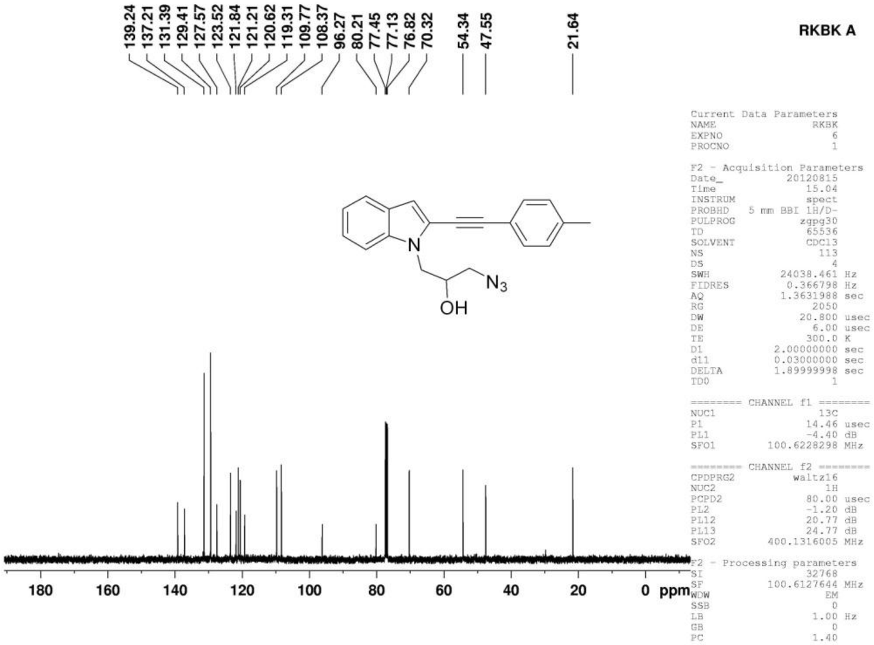

Figure 30: ^{13}C NMR of 5a

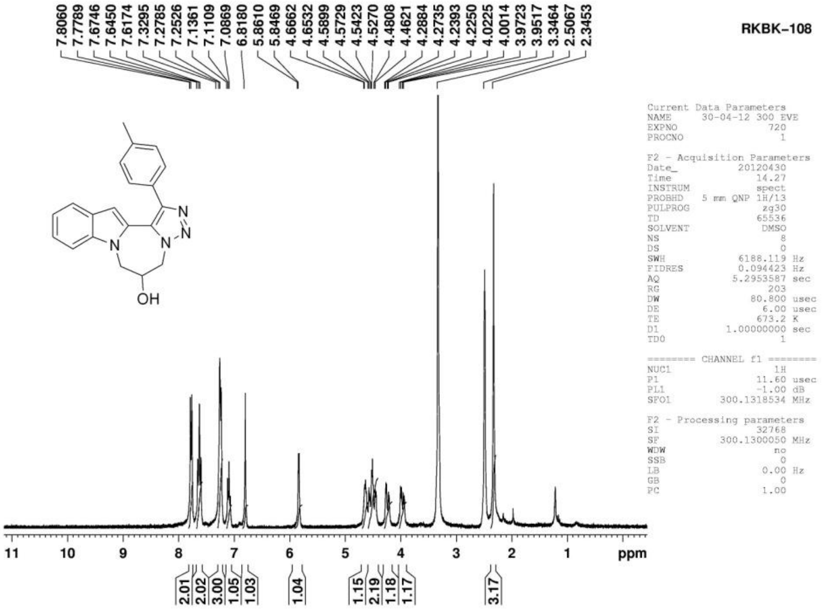

Figure 31: ^{1}H NMR of 6a

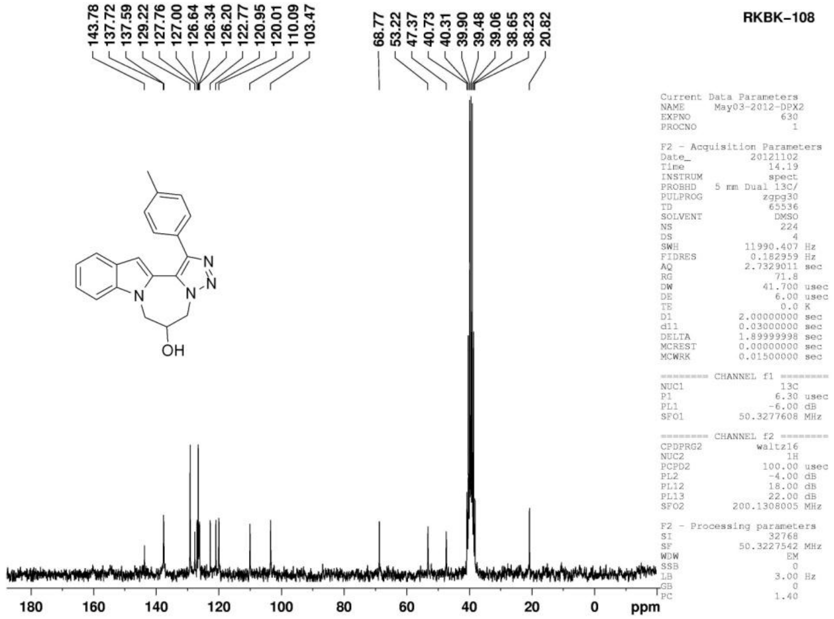

Figure 32: ^{13}C NMR of 6a

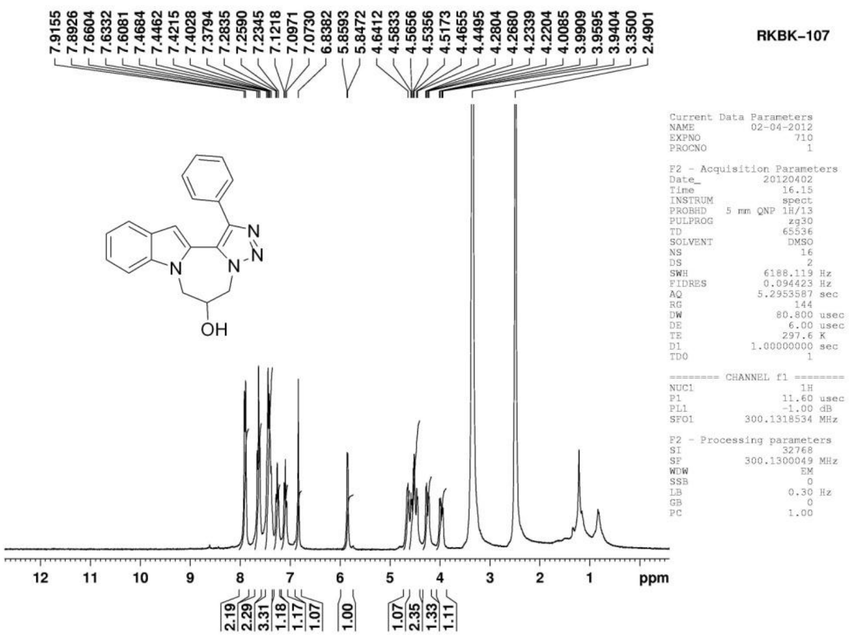

Figure 33: ^{1}H NMR of 6b

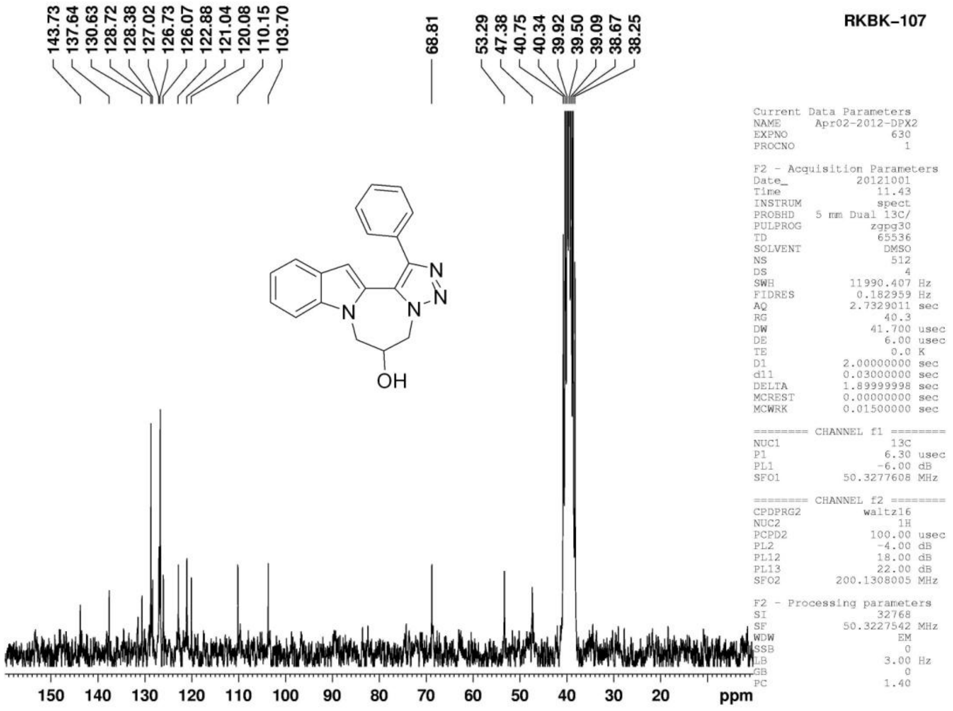

Figure 34: ^{13}C NMR of 6b

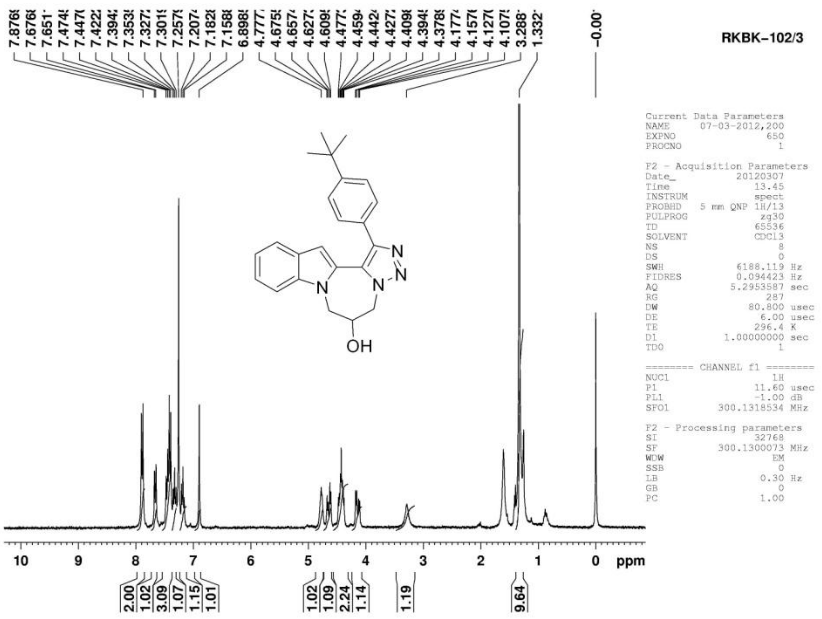

Figure 35: ^{1}H NMR of 6c

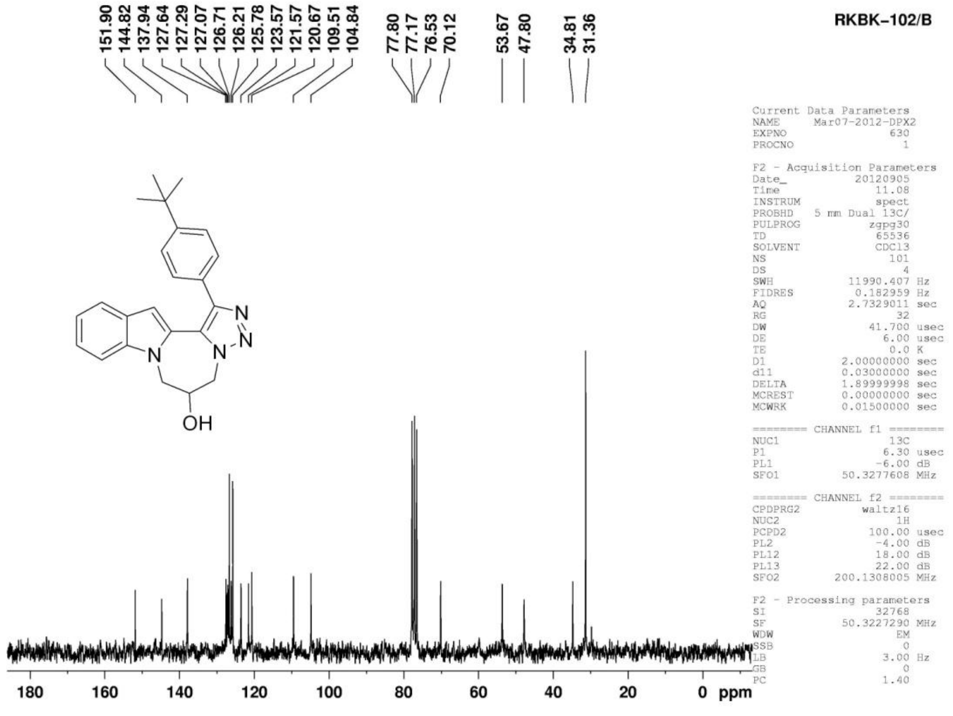

:

Figure 36: ^{13}C NMR of 6c

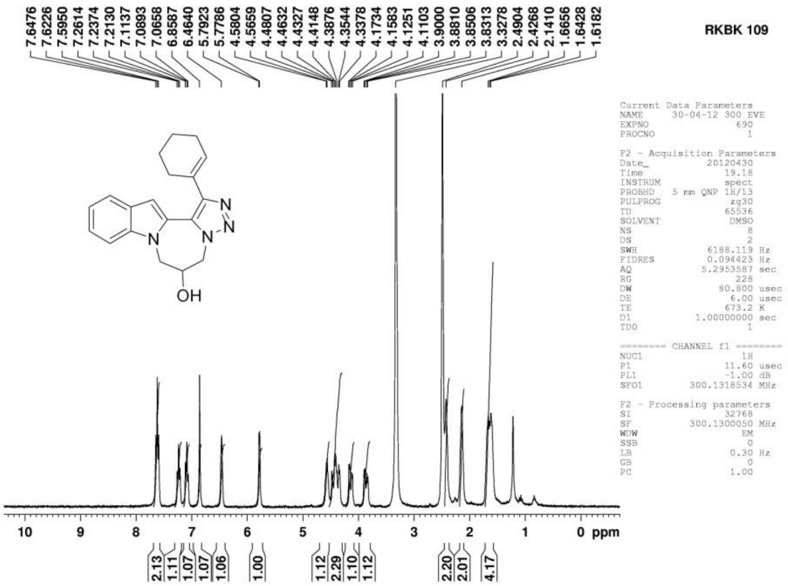

Figure 37: ^{1}H NMR of 6d

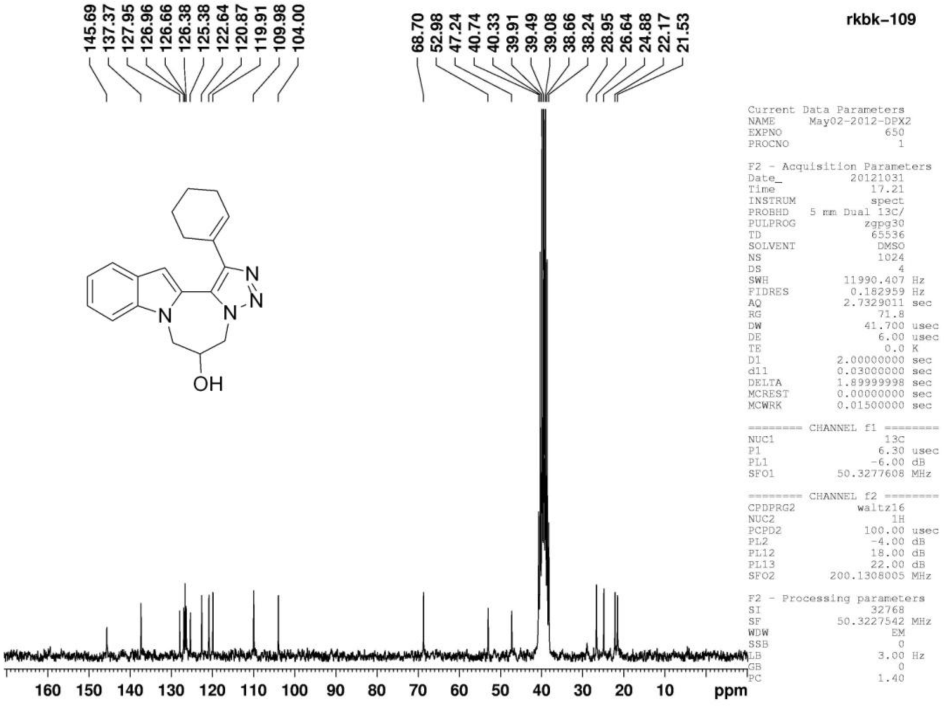

Figure 38: ^{13}C NMR of 6d

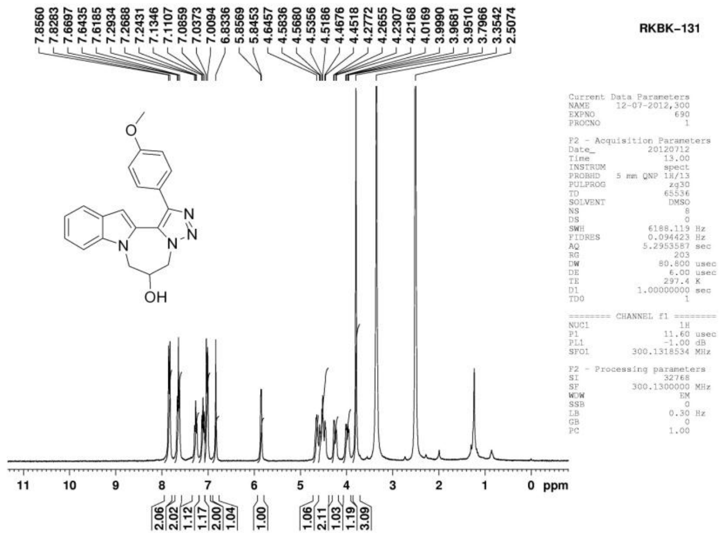

Figure 39: ^{1}H NMR of 6e

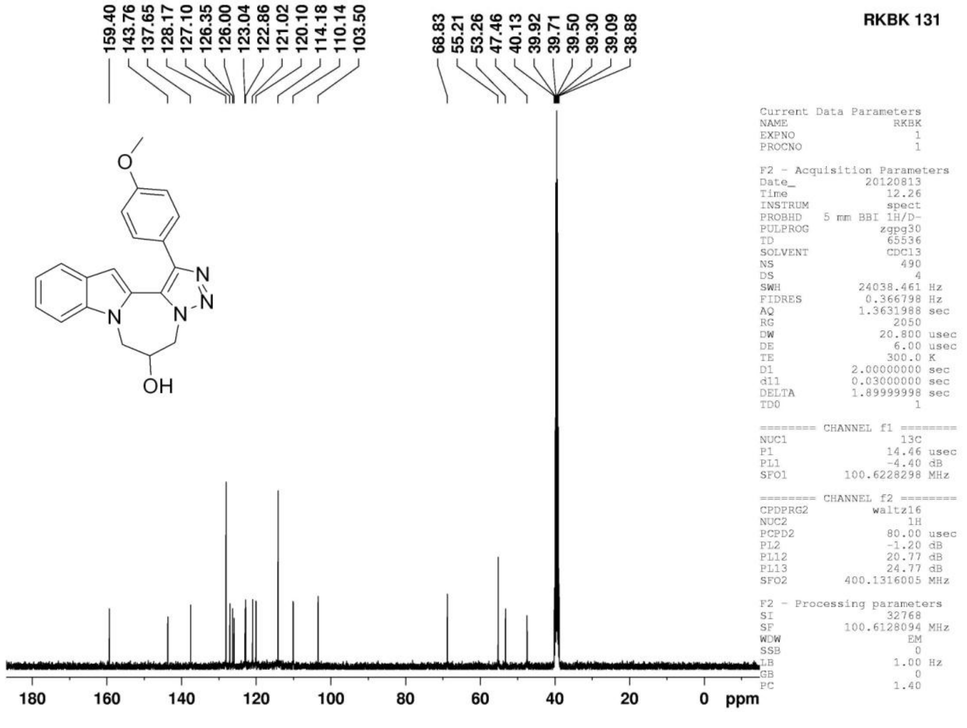

Figure 40: ^{13}C NMR of 6e

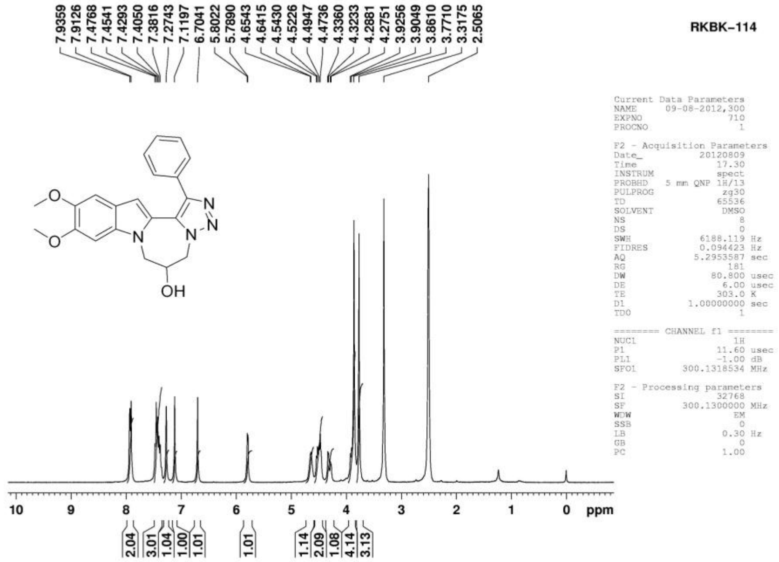

Figure 41: ^{1}H NMR of 6f

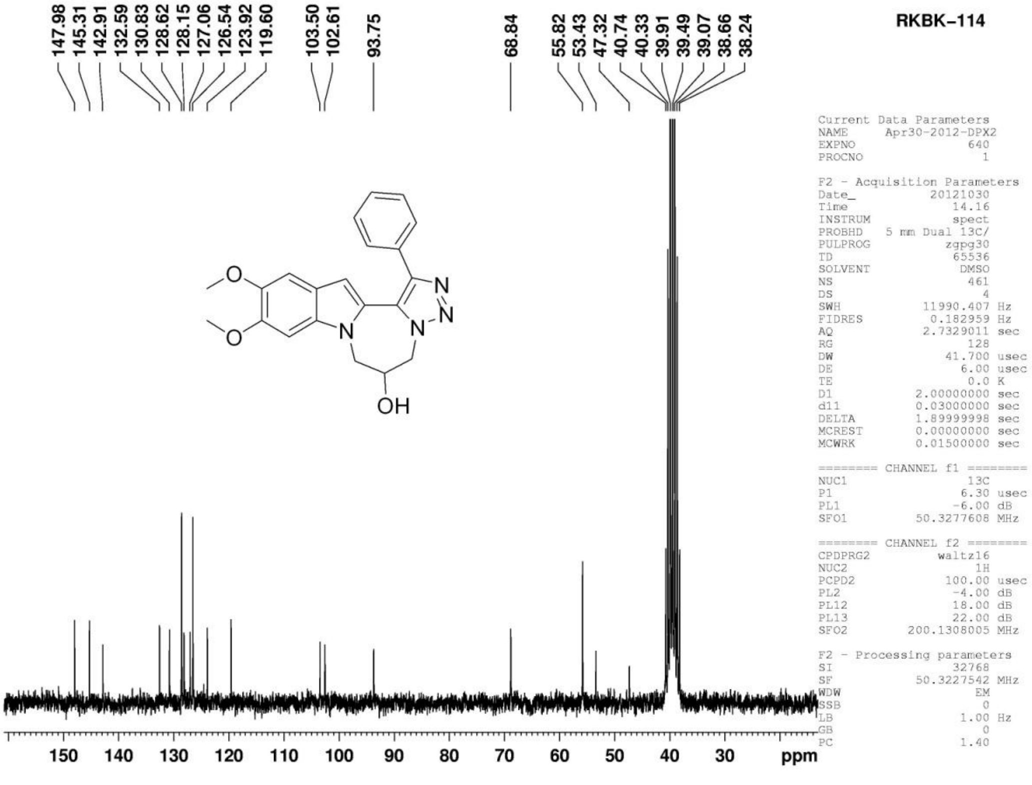

Figure 42: ^{13}C NMR of 6f

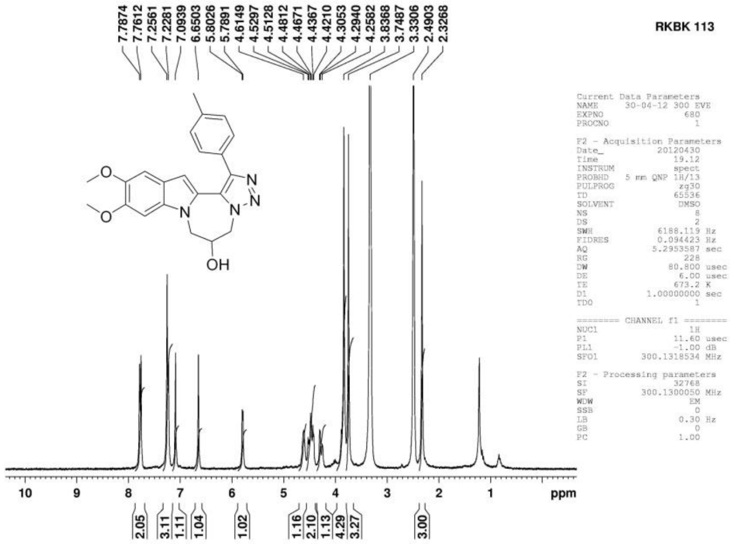

Figure 43: ^{1}H NMR of 6g

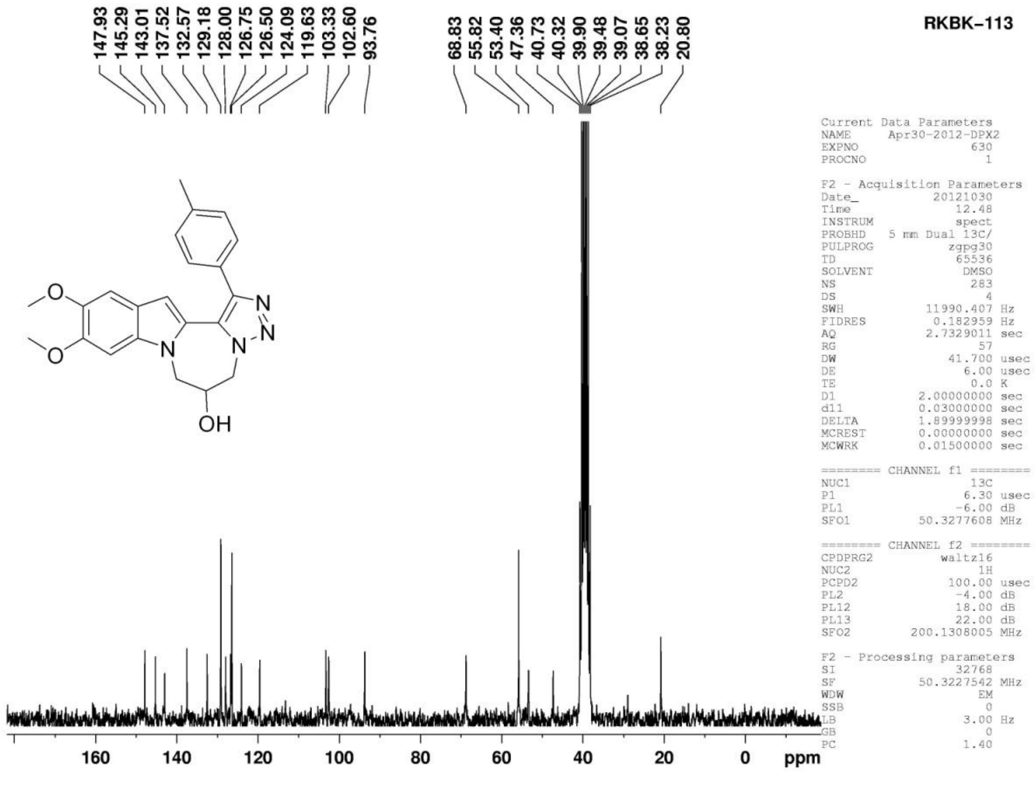

Figure 44: ^{13}C NMR of 6g

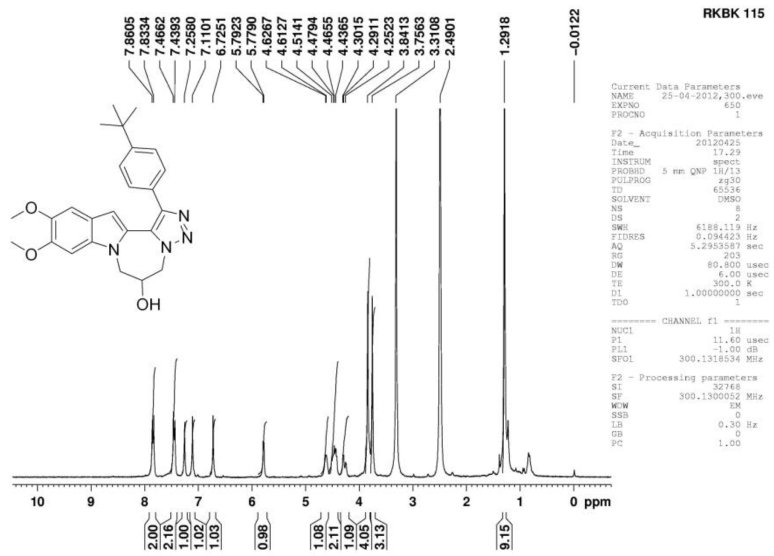

Figure 45: ^{1}H NMR of 6h

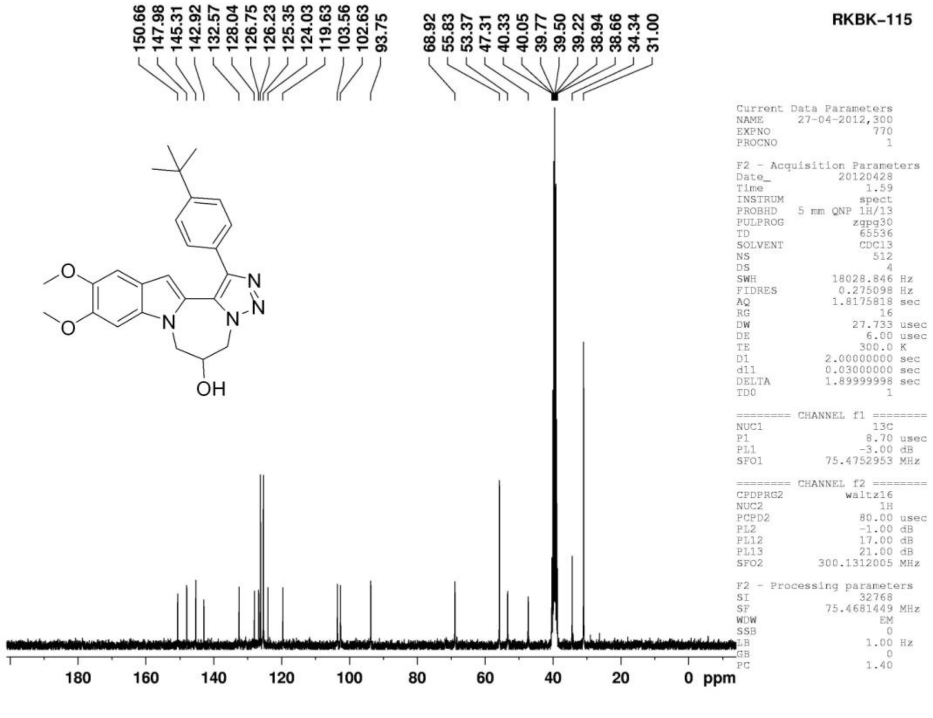

Figure 46: ^{13}CNMR of 6h

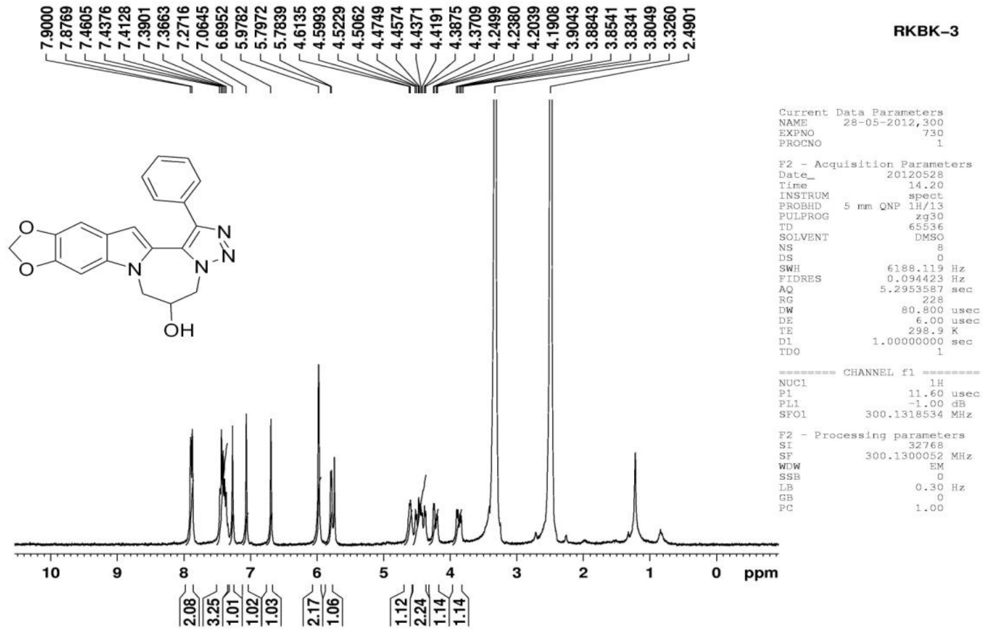

Figure 47: ^{1}H NMR of 6i

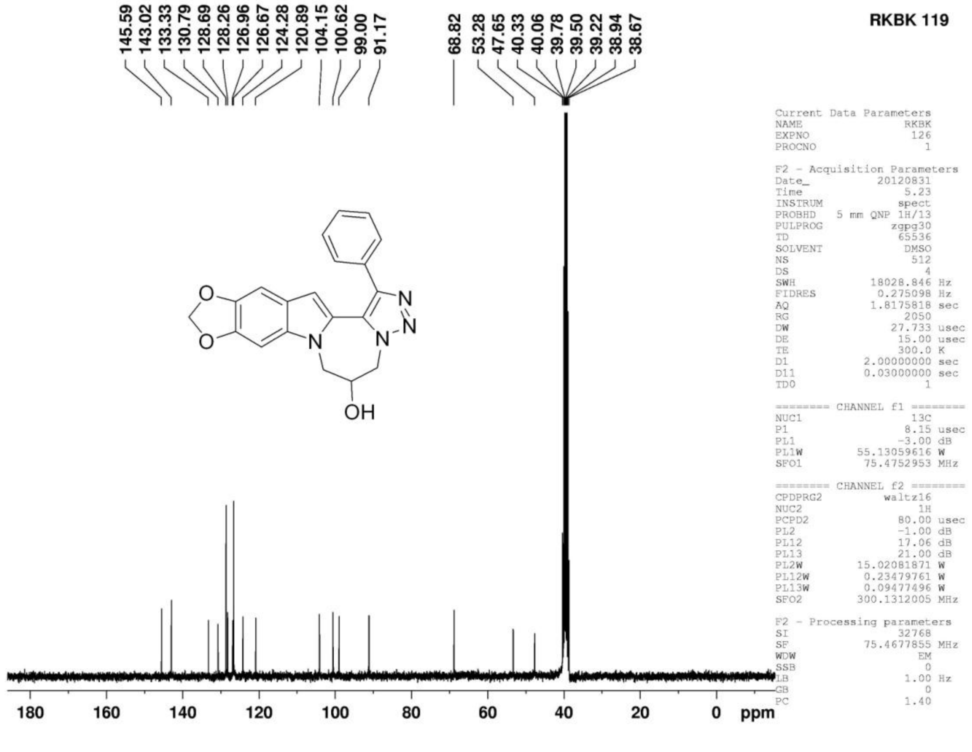

Figure 48: ^{13}C NMR of 6i

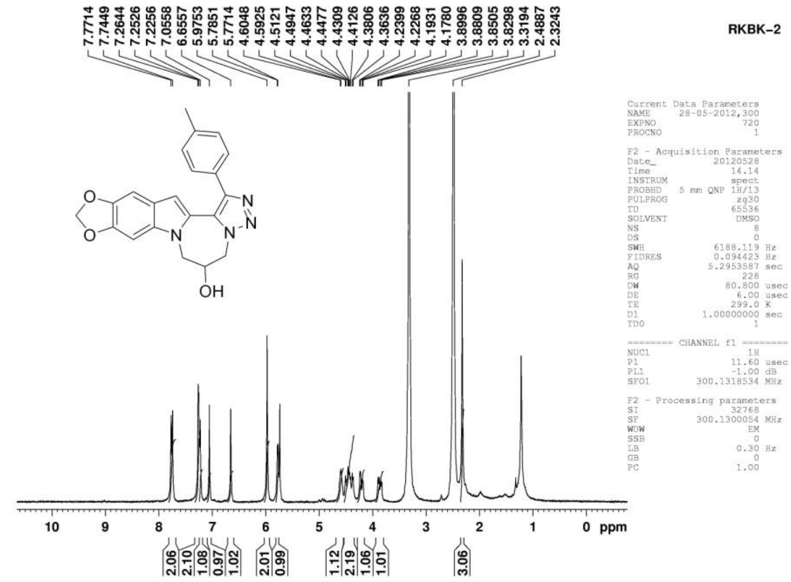

Figure 49: ^{1}H NMR of 6j

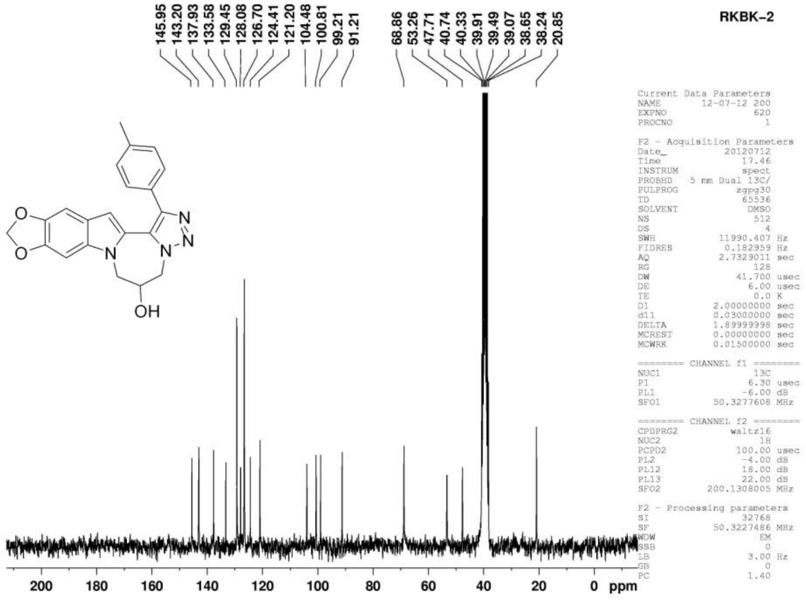

Figure 50: ^{13}C NMR of 6i

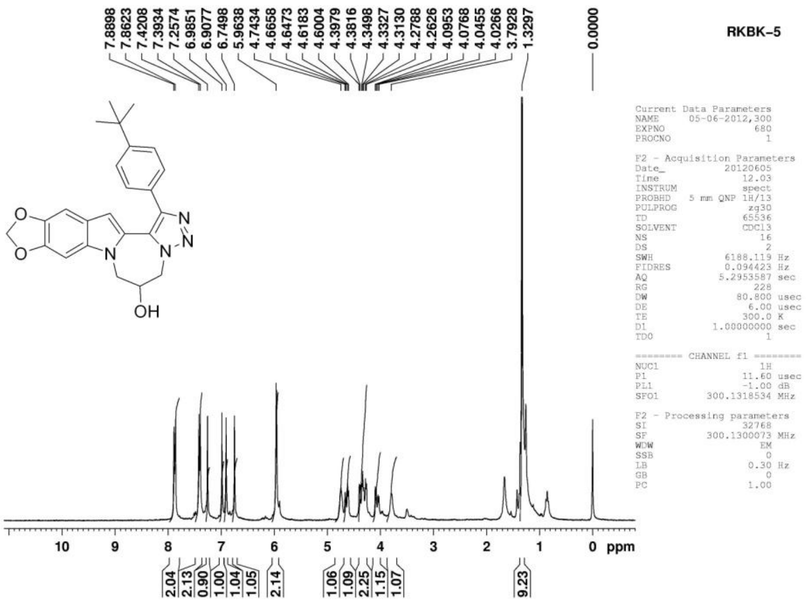

Figure 51: ^{1}H NMR of 6k

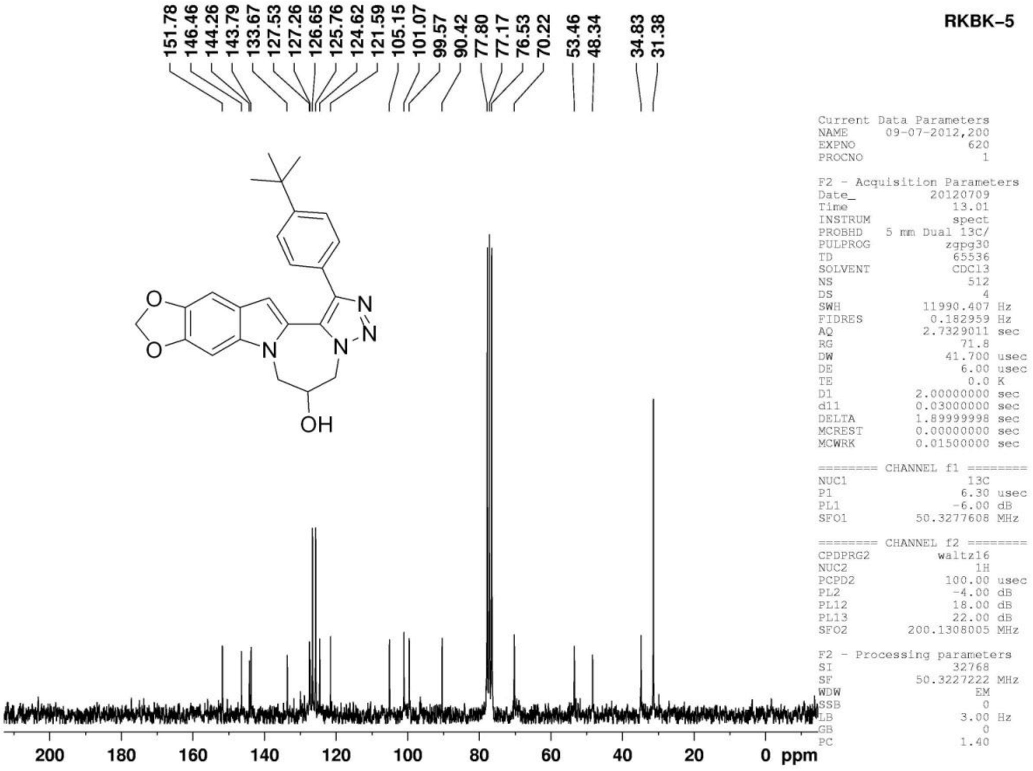

Figure 52: ^{13}C NMR of 6k

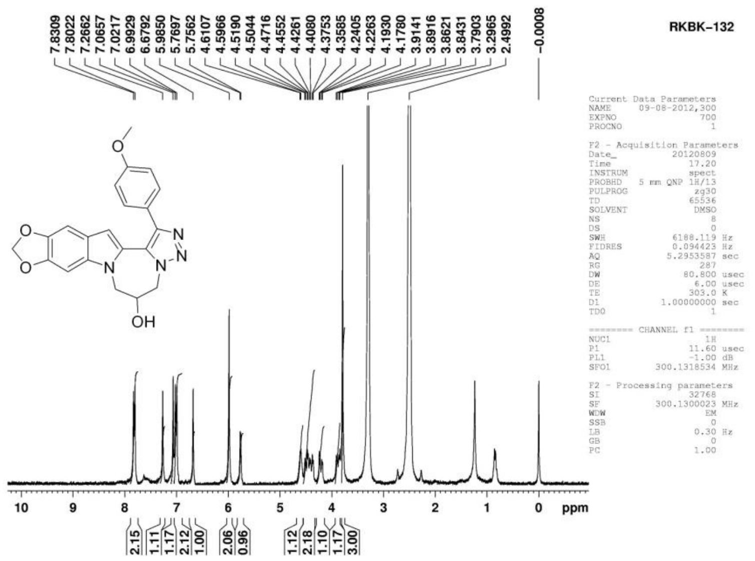

Figure 53: ^{1}H NMR of 6l

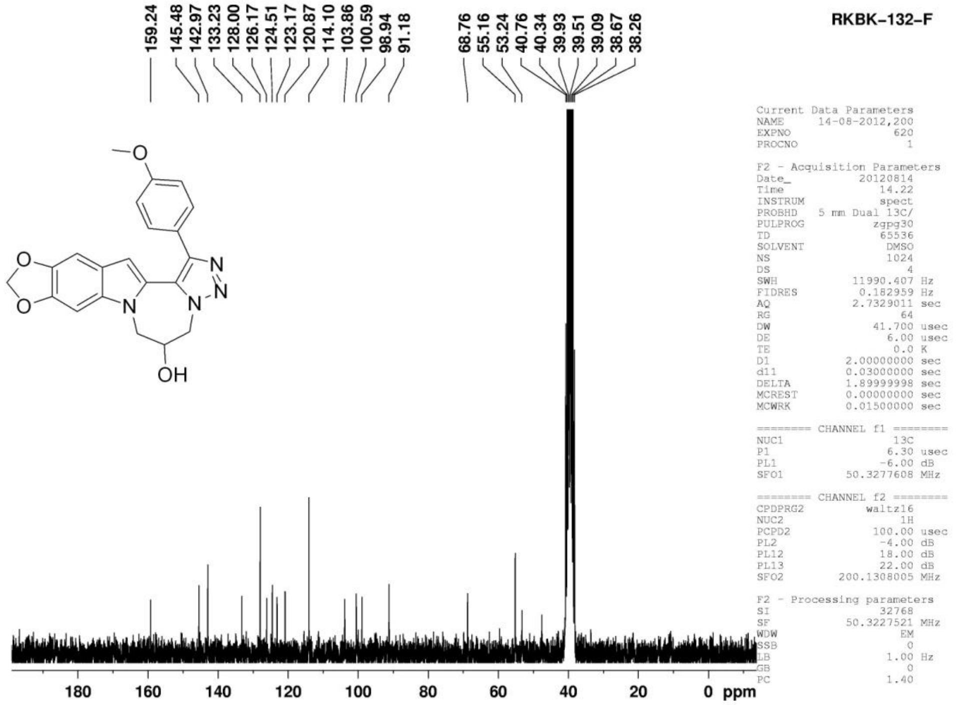

Figure 54: ^{13}C NMR of 6l

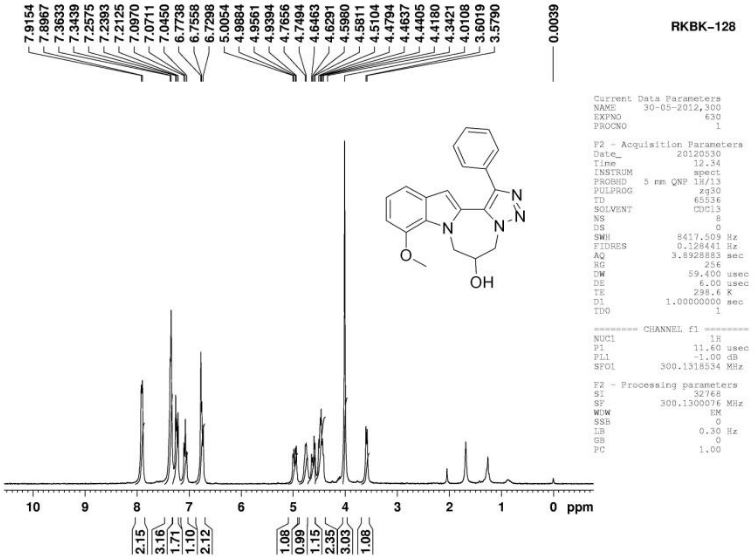

Figure 55: ^{1}H NMR of 6m

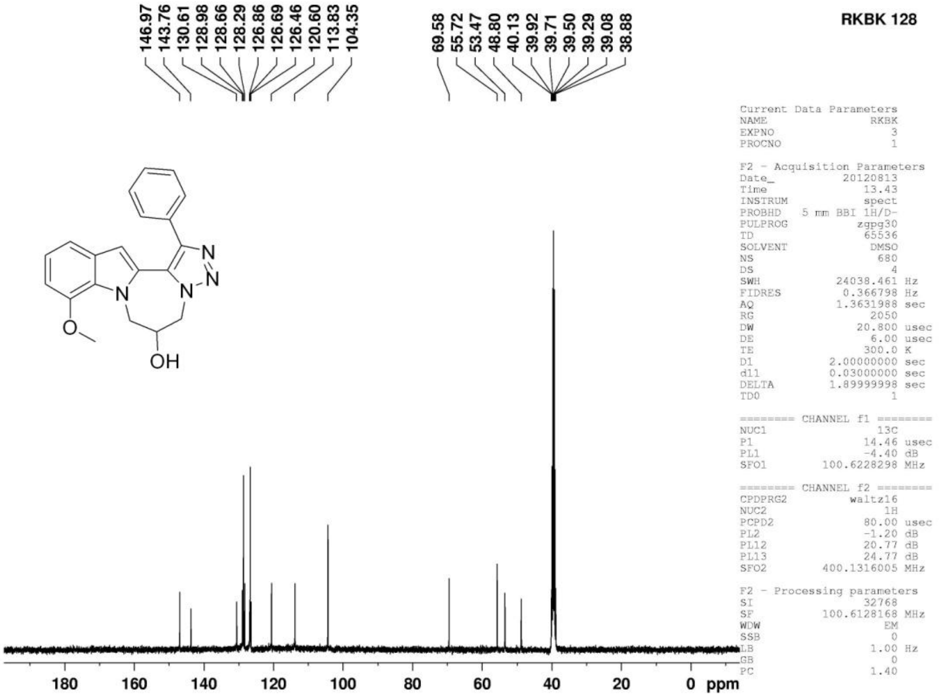

Figure 56: ^{13}C NMR of 6m

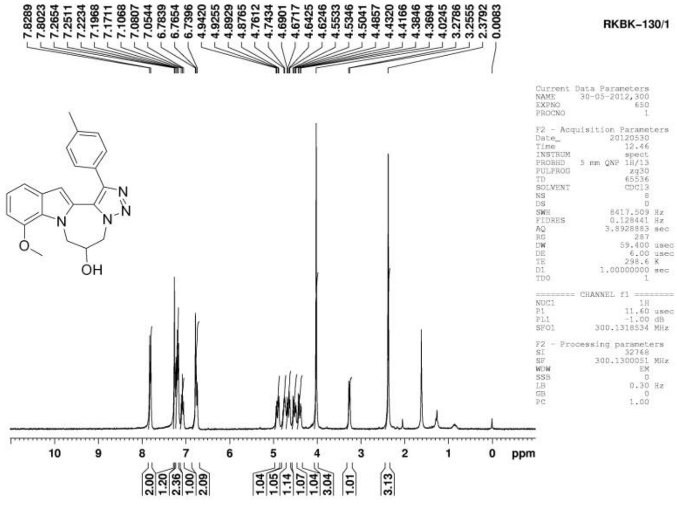

Figure 57: ^{1}H NMR of 6n

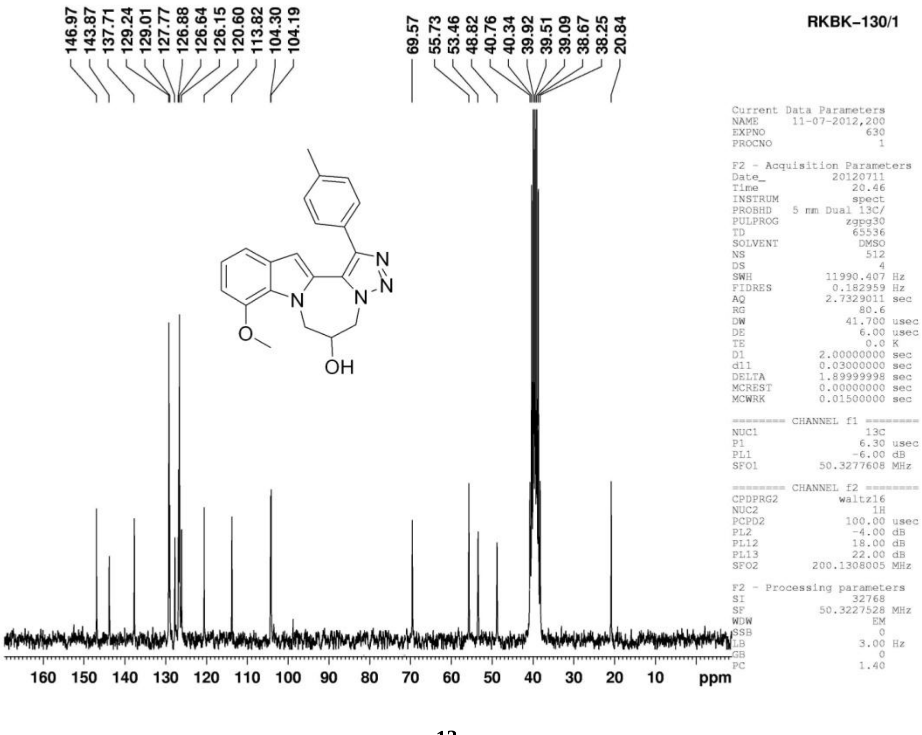

Figure 58: ^{13}C NMR of 6n

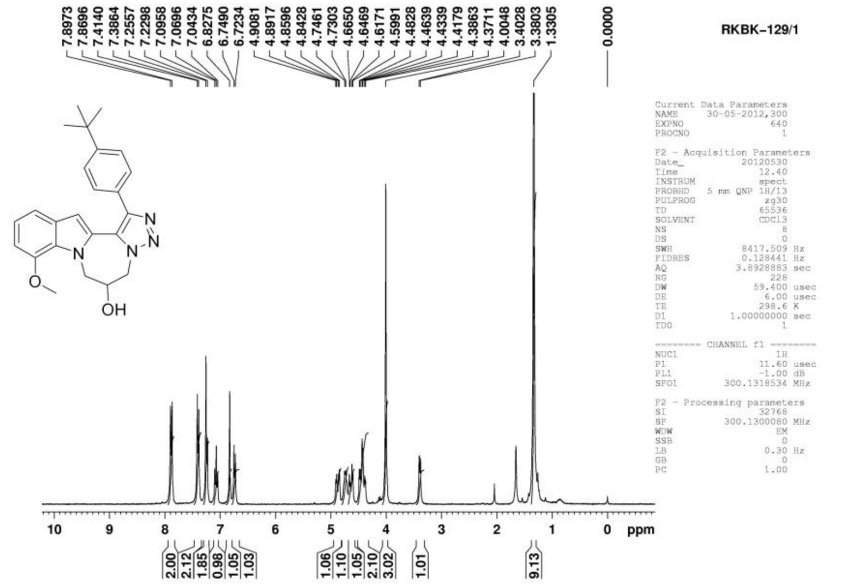

Figure 59: ^{1}H NMR of 6o

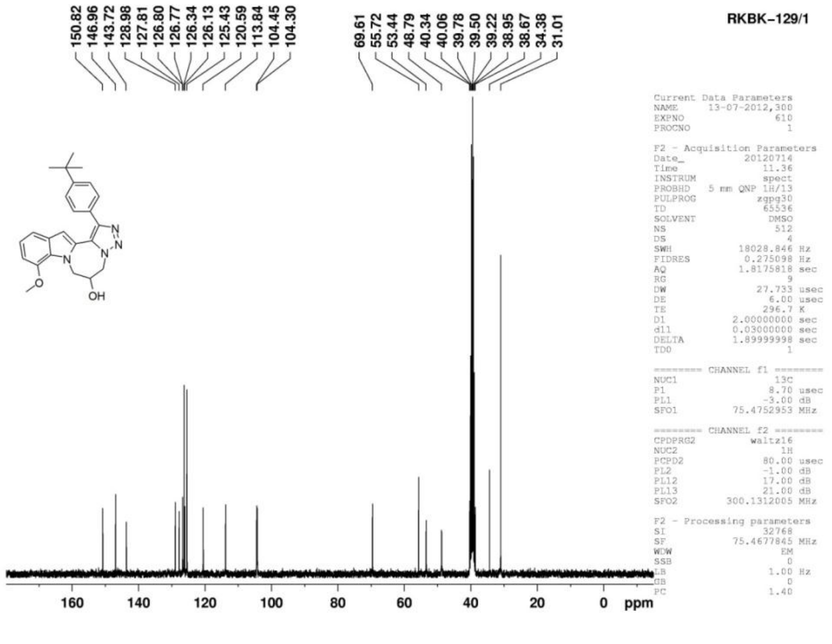

Figure 60: ^{13}C NMR of 6o

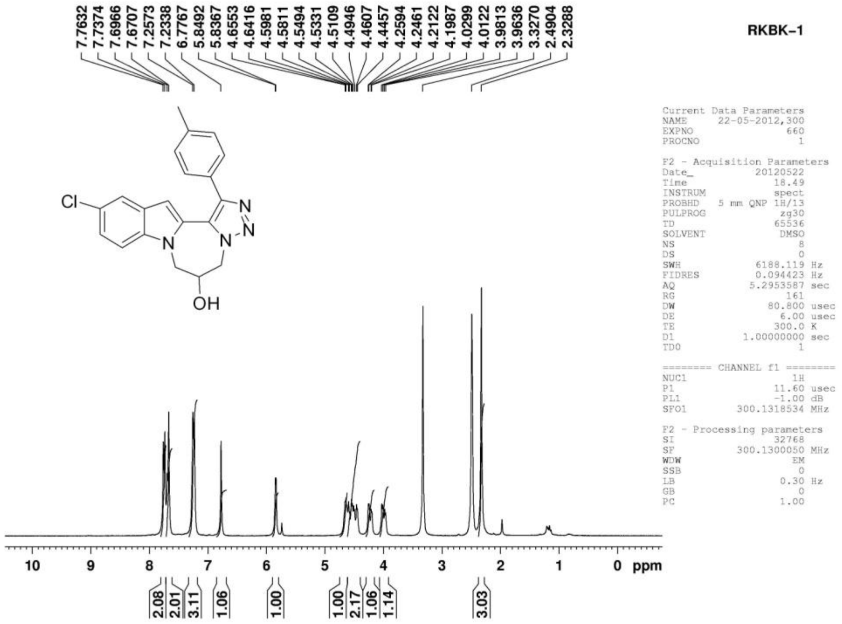

Figure 61: ^{1}H NMR of 6p

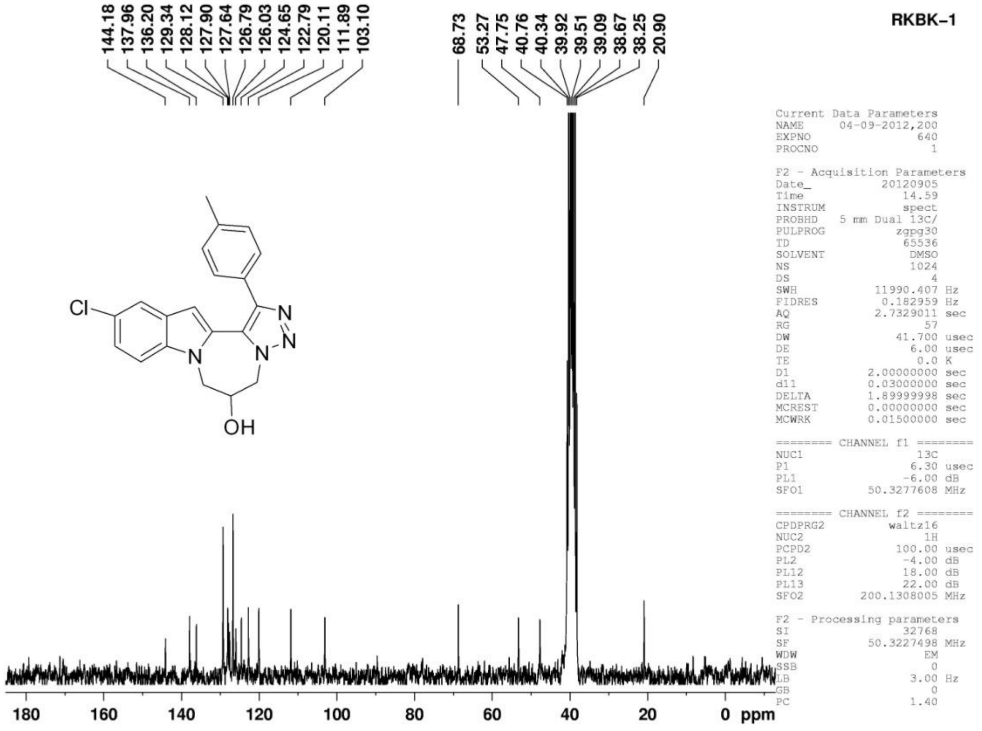

Figure 62: ^{13}C NMR of 6p

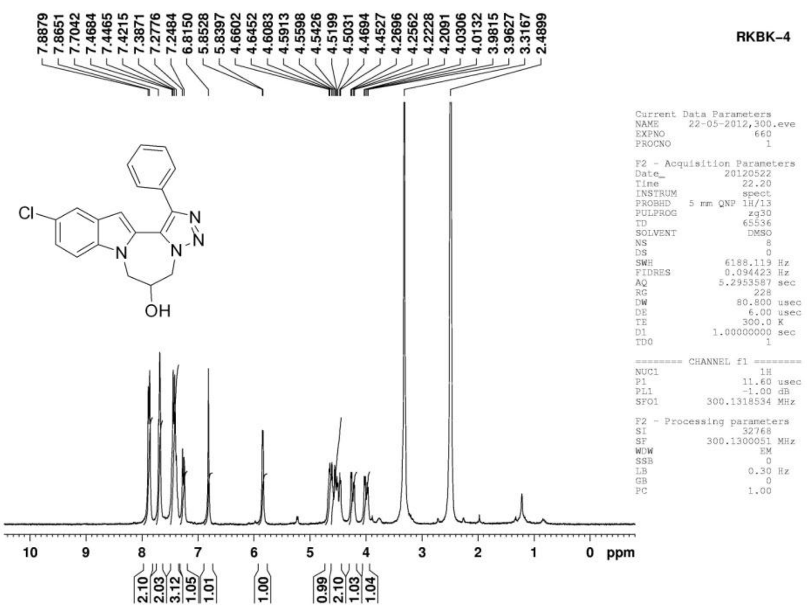

Figure 63: ^{1}H NMR of 6q

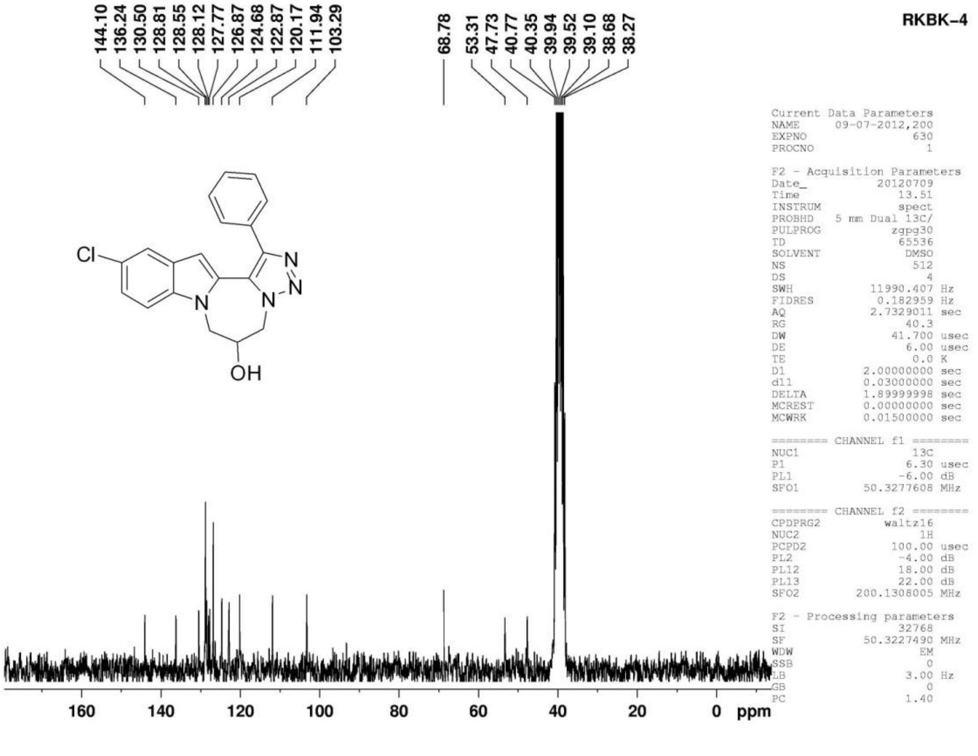

Figure 64: ^{13}C NMR of 6q

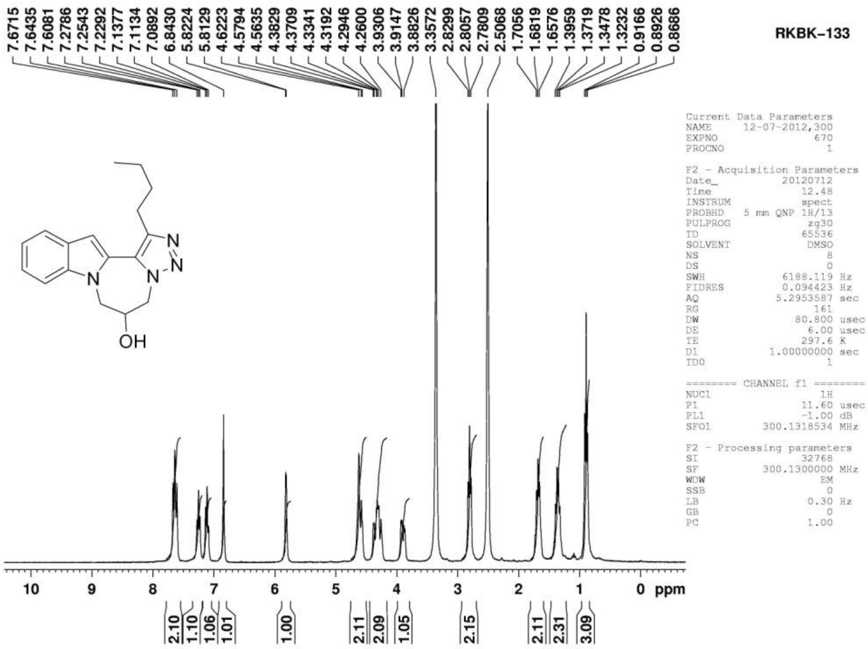

Figure 65: ^{1}H NMR of 6r

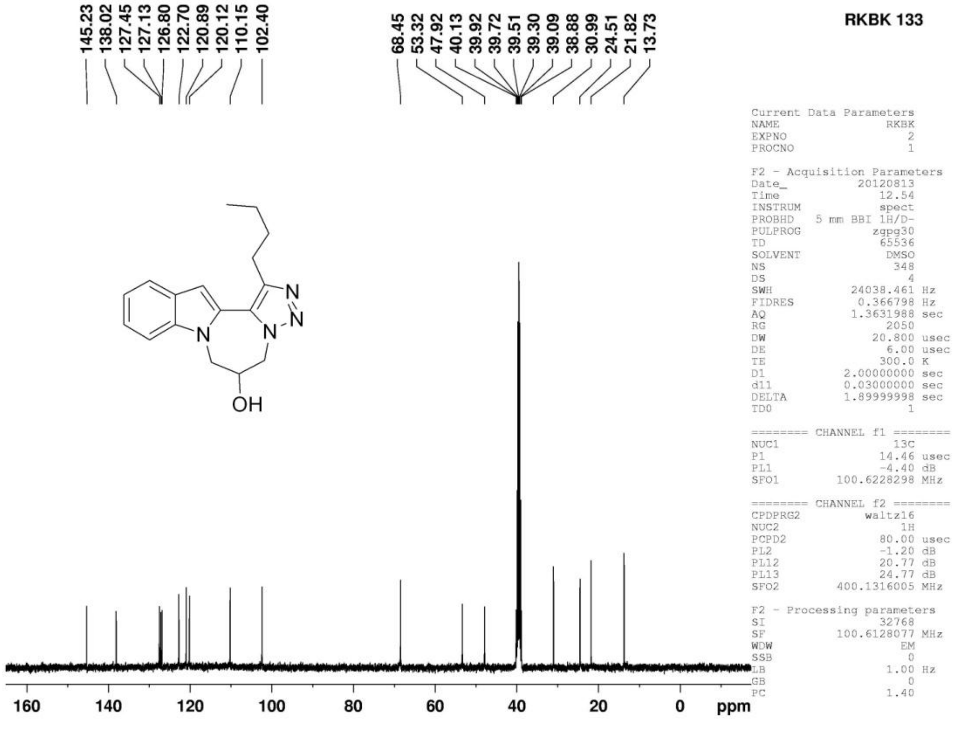

Figure 66: ^{13}C NMR of 6r

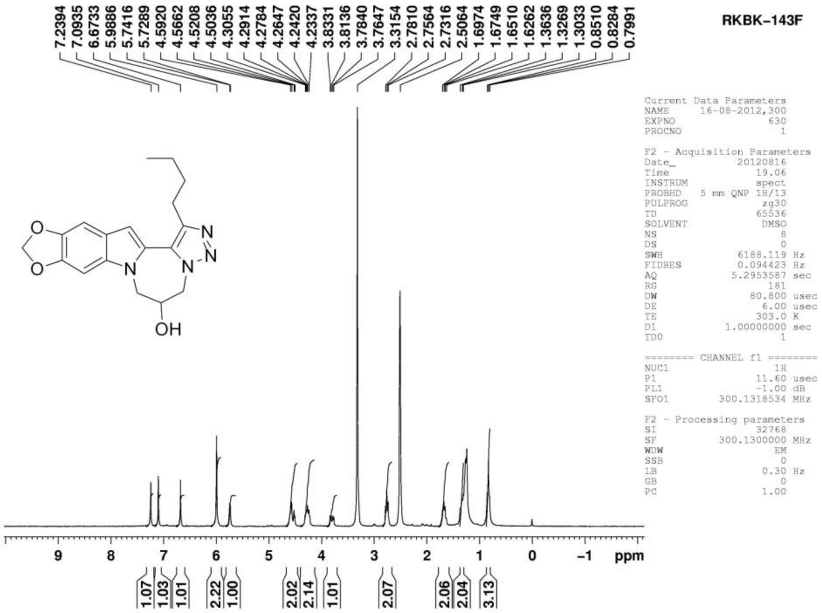

Figure 67: ^{1}H NMR of 6s

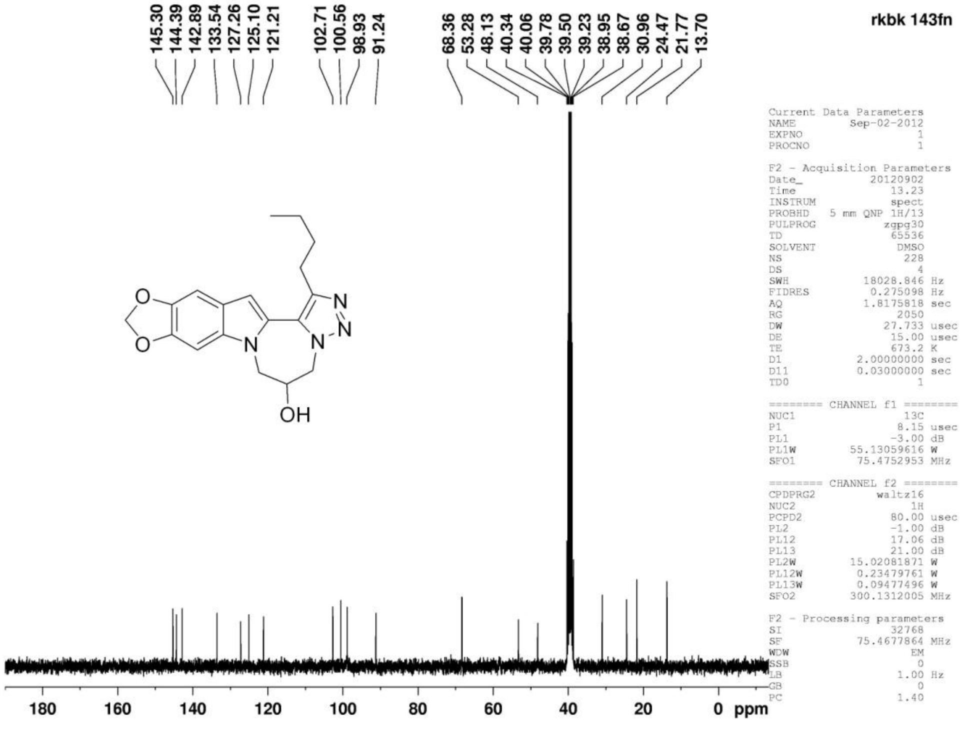

Figure 68: ^{13}C NMR of 6s

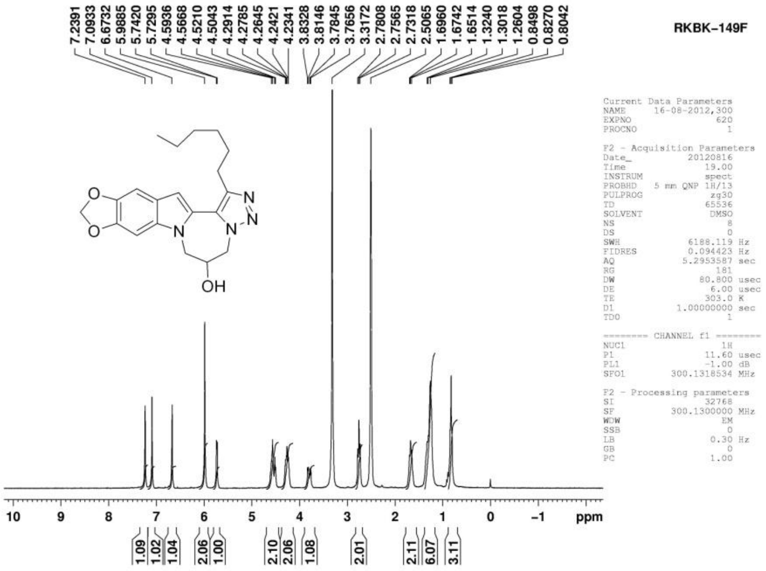

Figure 69: ^{1}H NMR of 6t

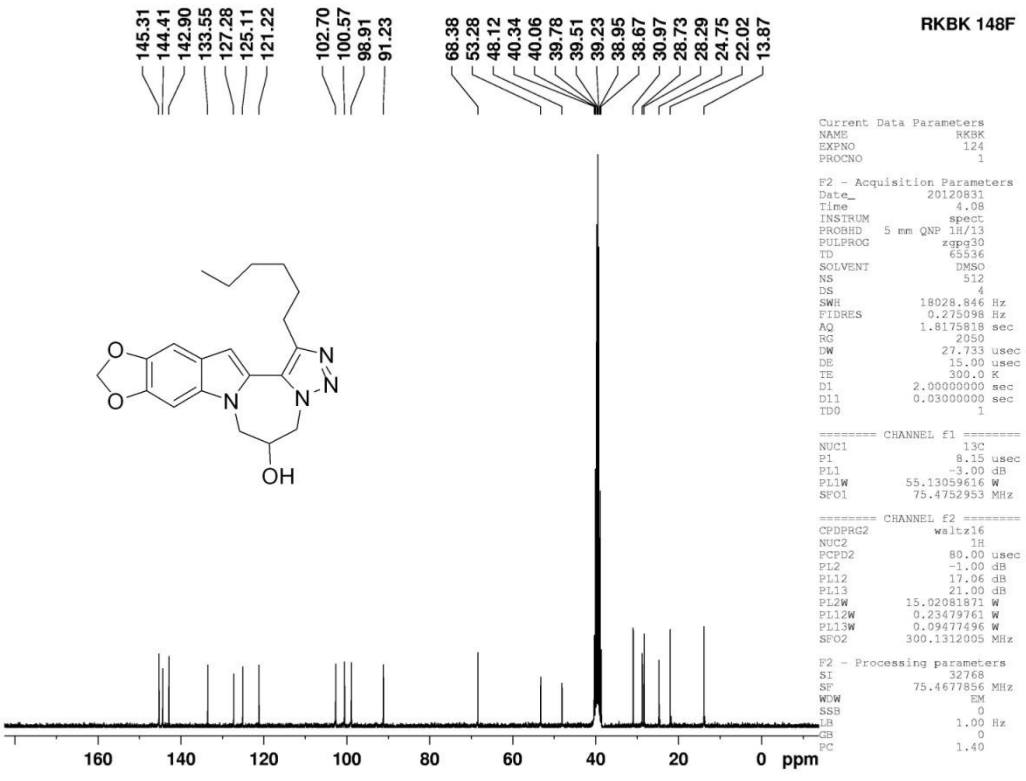

Figure 70: ^{13}C NMR of 6t

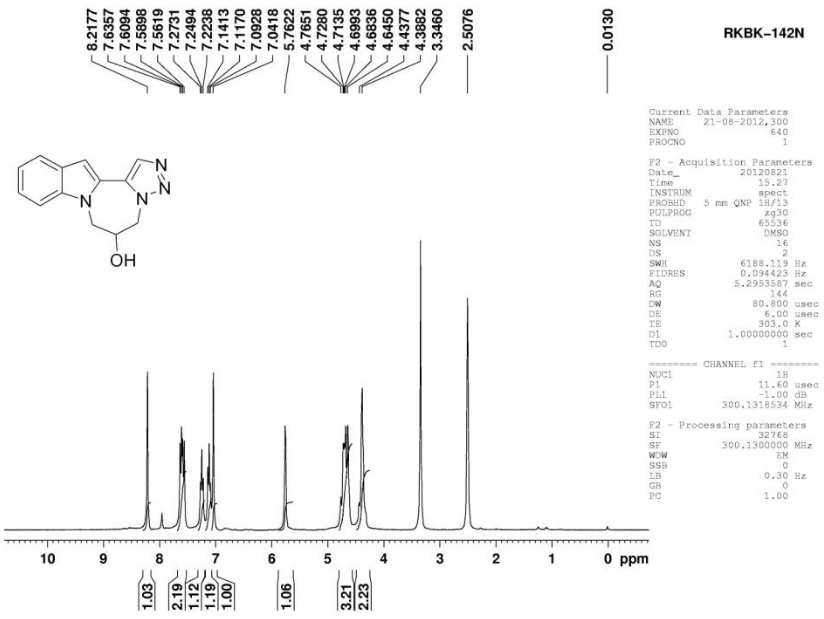

Figure 71: ^{1}H NMR of 6u

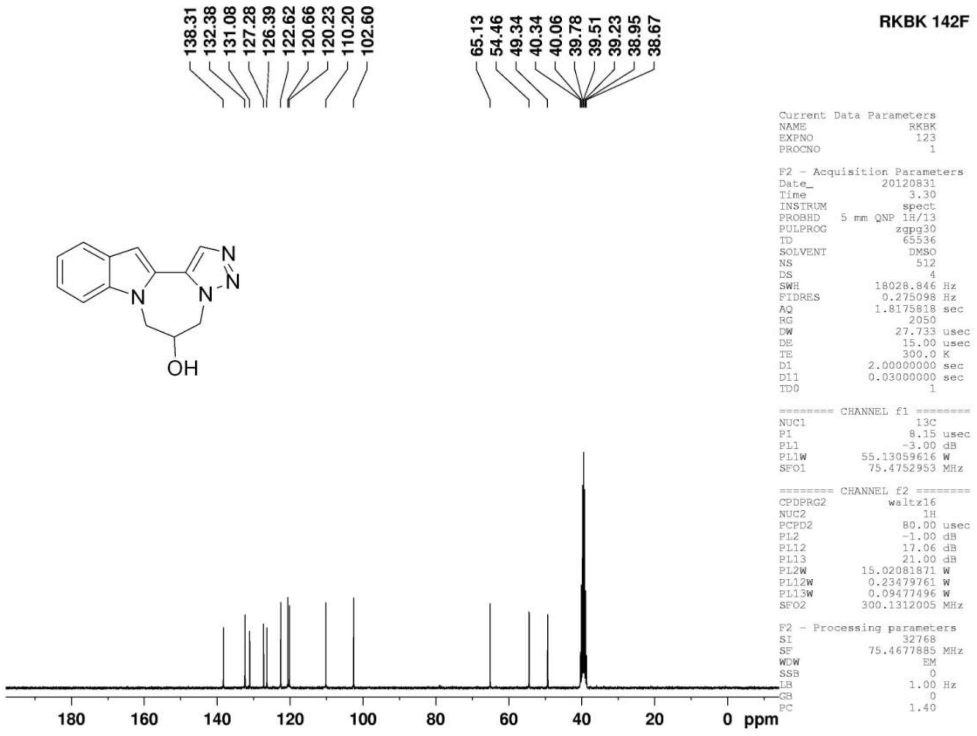

Figure 72: ^{13}C NMR of 6u

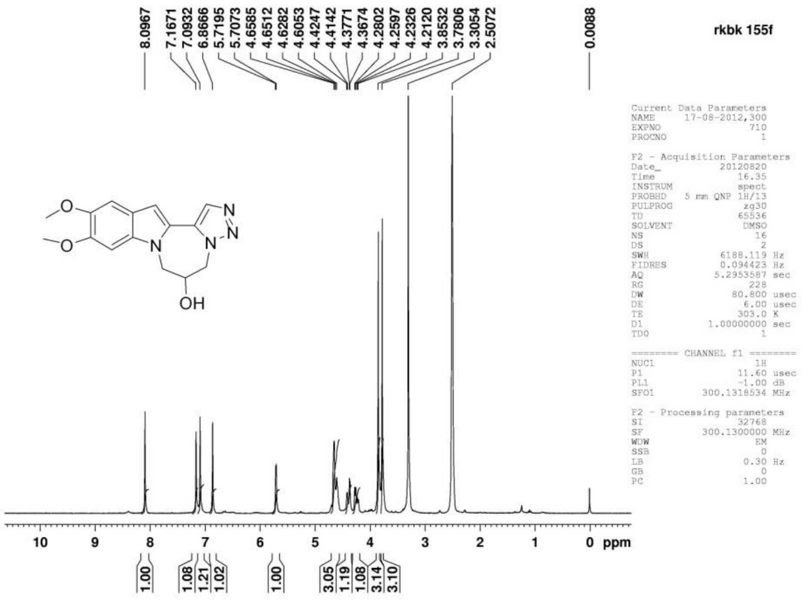

Figure 73: ^{1}H NMR of 6v

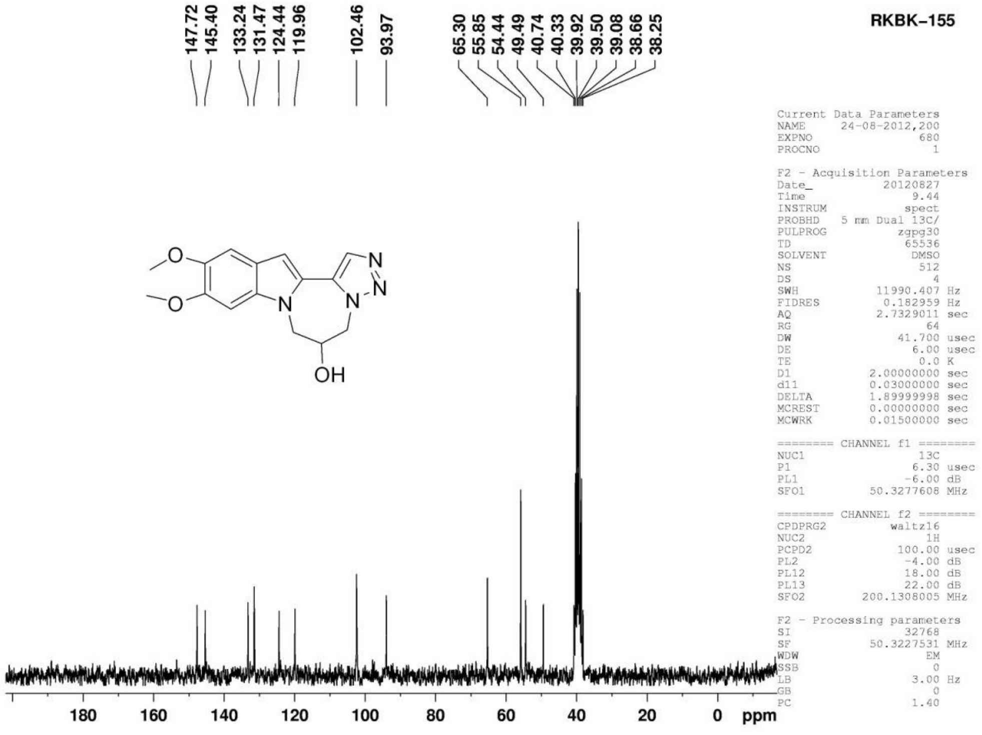

Figure 74: ^{13}C NMR of 6v

Copies of HRMS of starting and final compounds

For Copies of ^{1}H &^{13}C NMR of componds **1a-1d**, **1f**, **1g**, **1i**, **1m**, **1u** see reference [1].

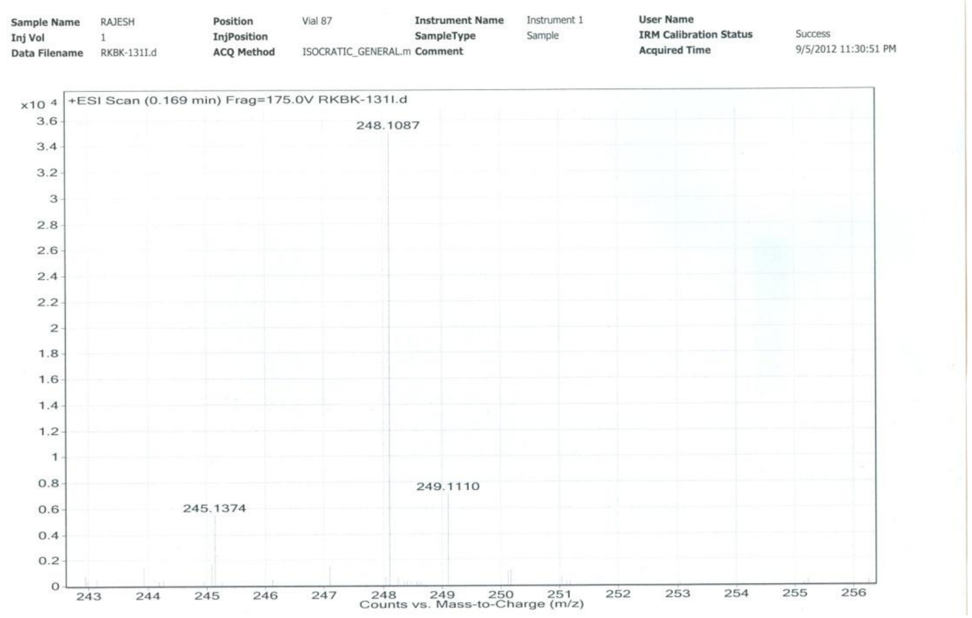

Figure 75: HRMS of 1e

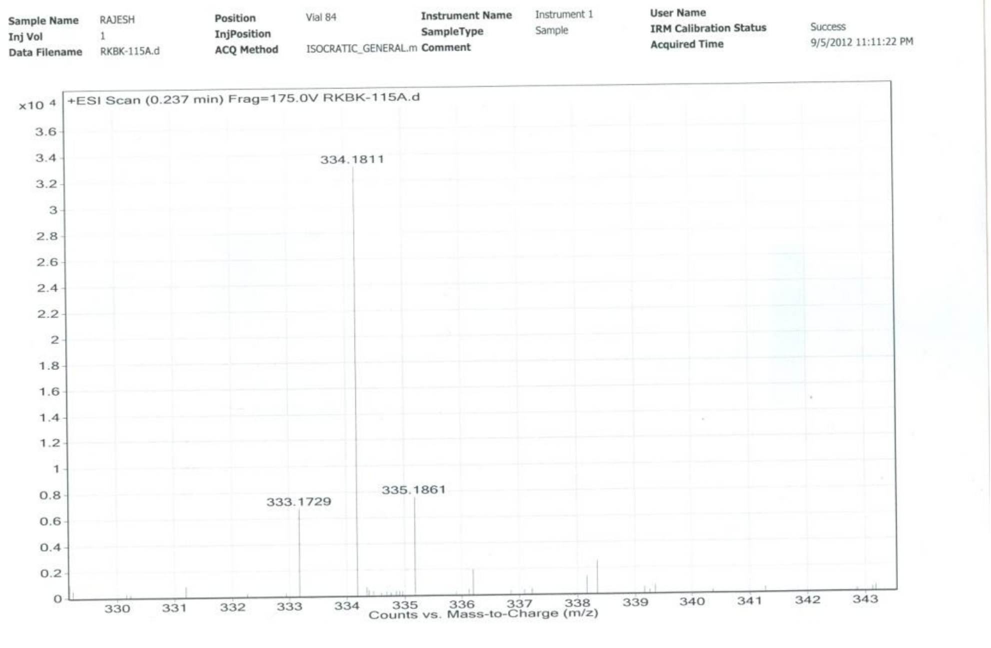

Figure 76:HRMS of 1h

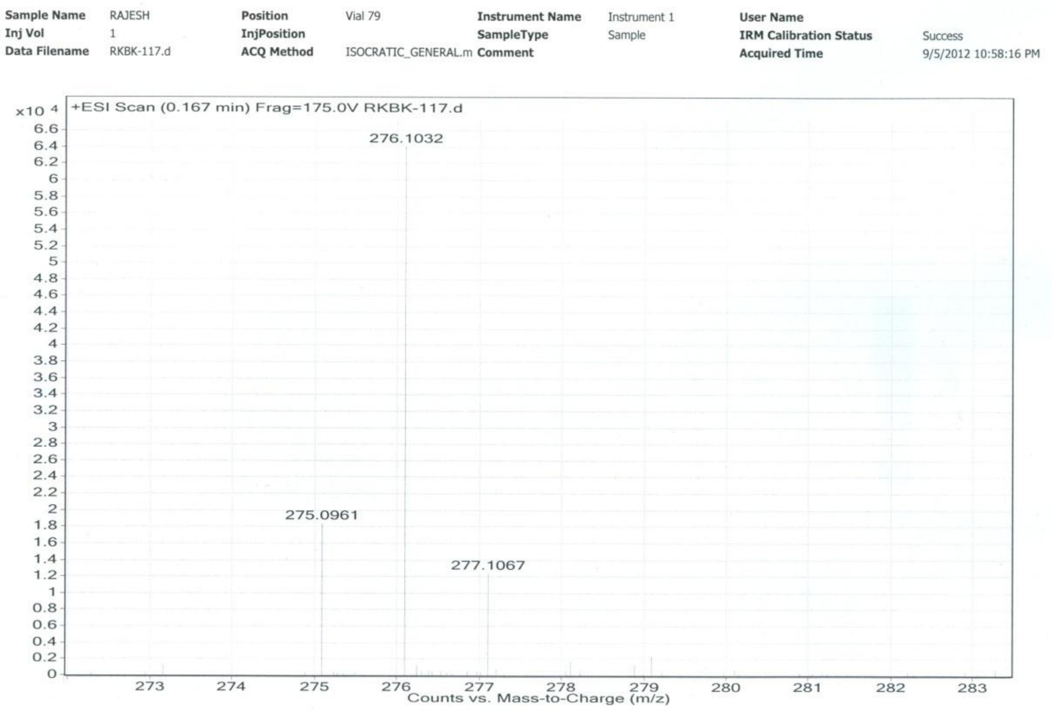

Figure 77: HRMS of 1j

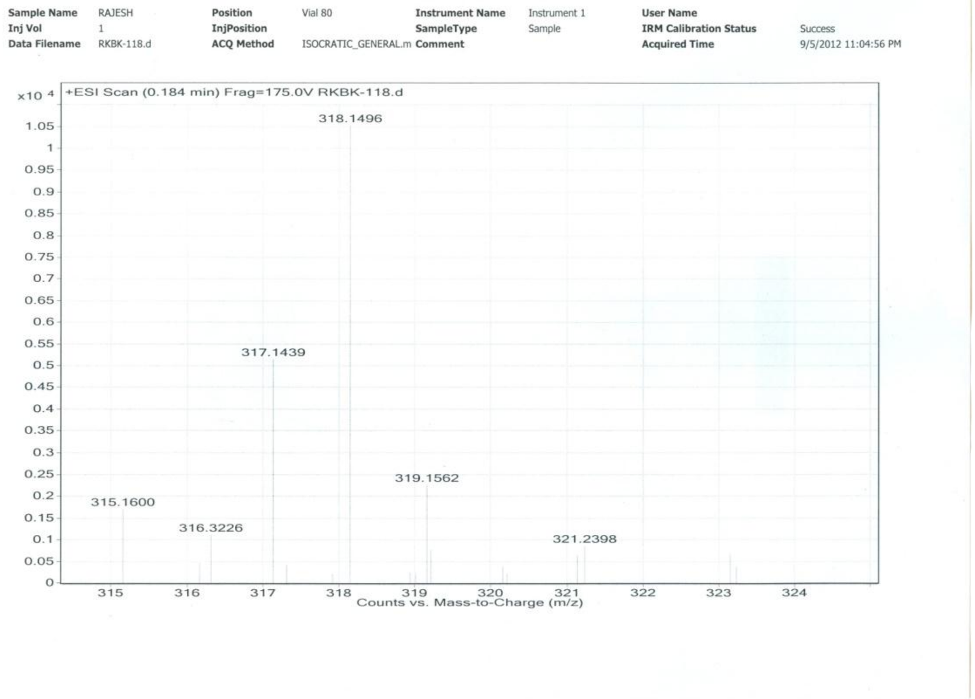

Figure 78: HRMS of 1k

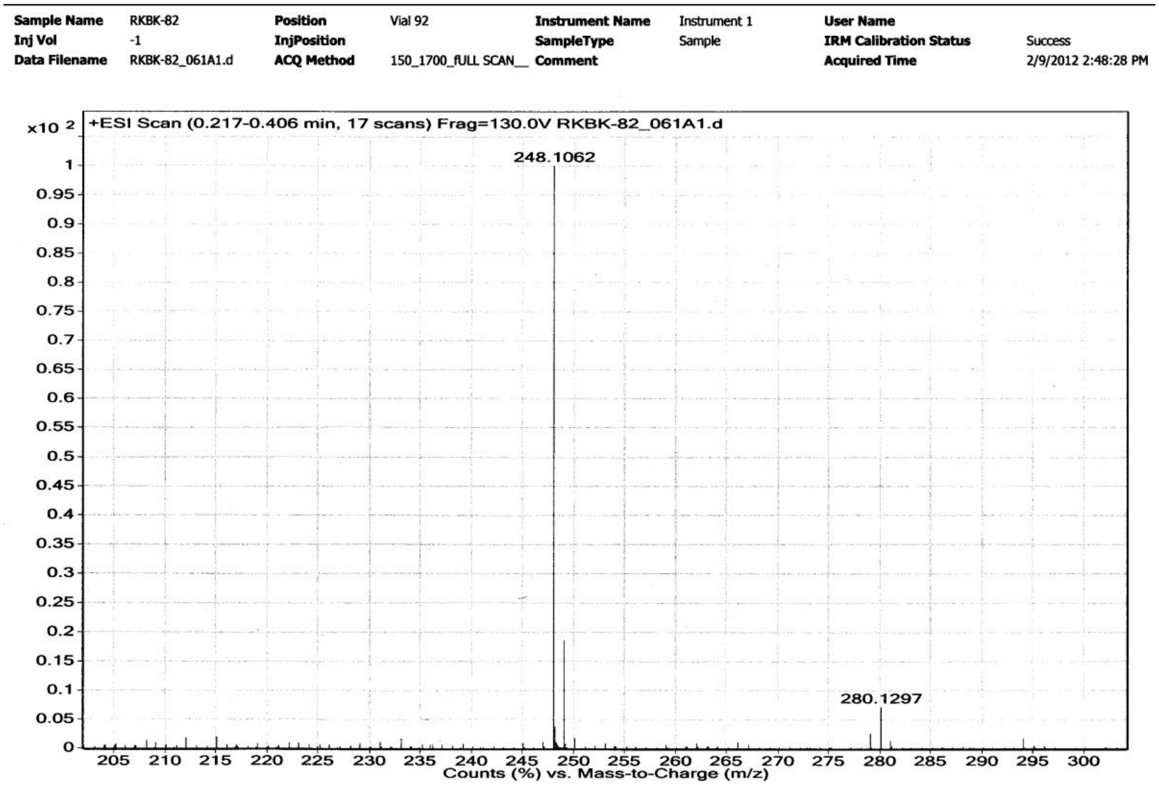

Figure 79: HRMS of 1l

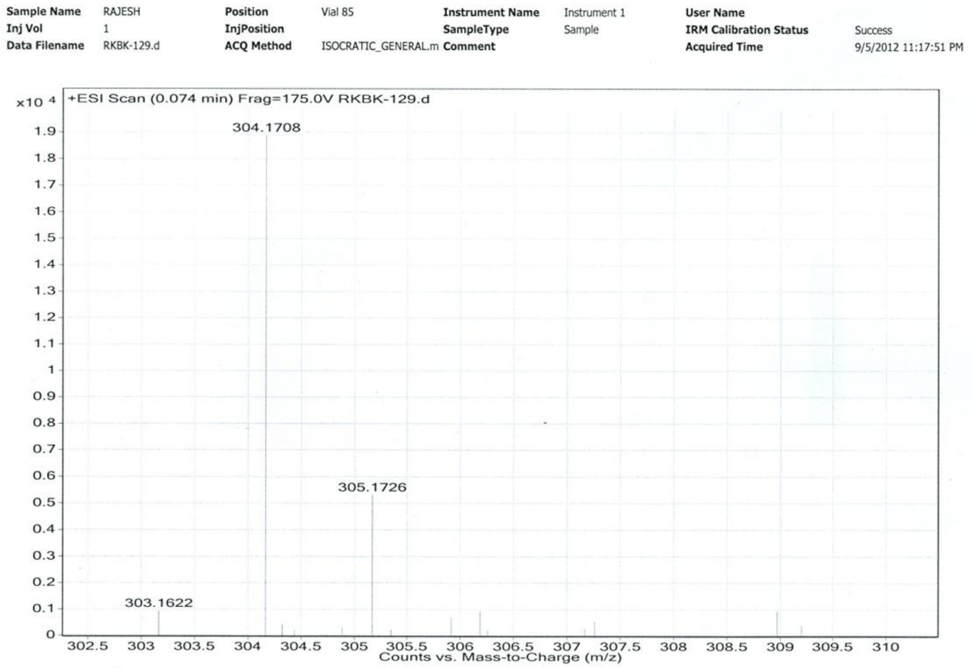

Figure 80: HRMS of 1n

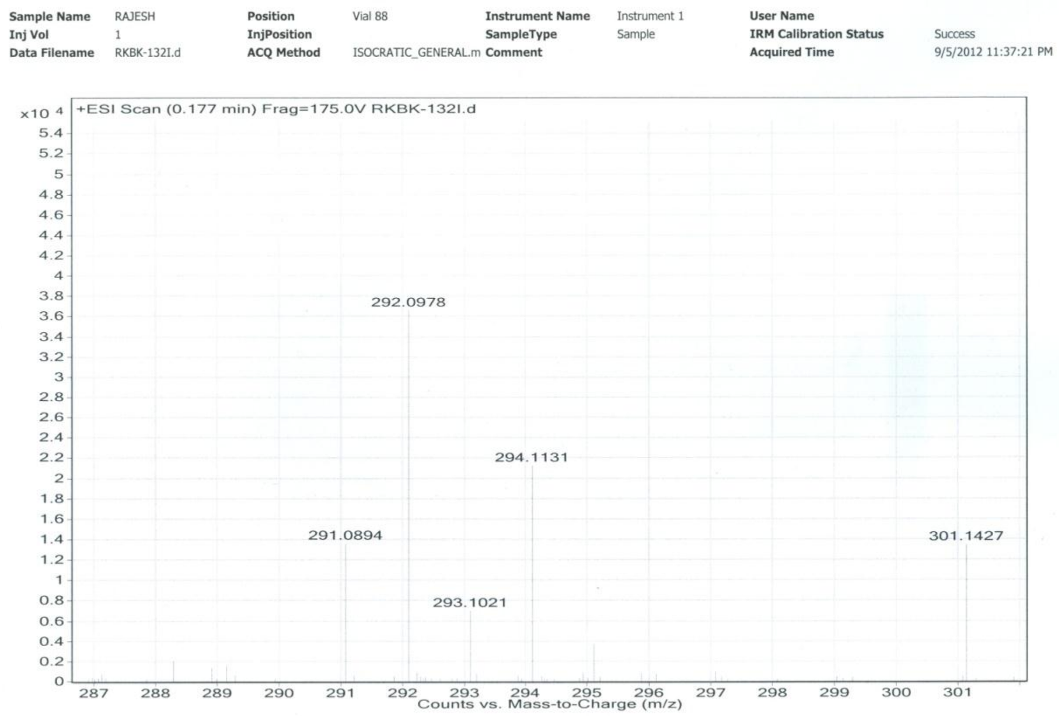

Figure 81: HRMS of 1o

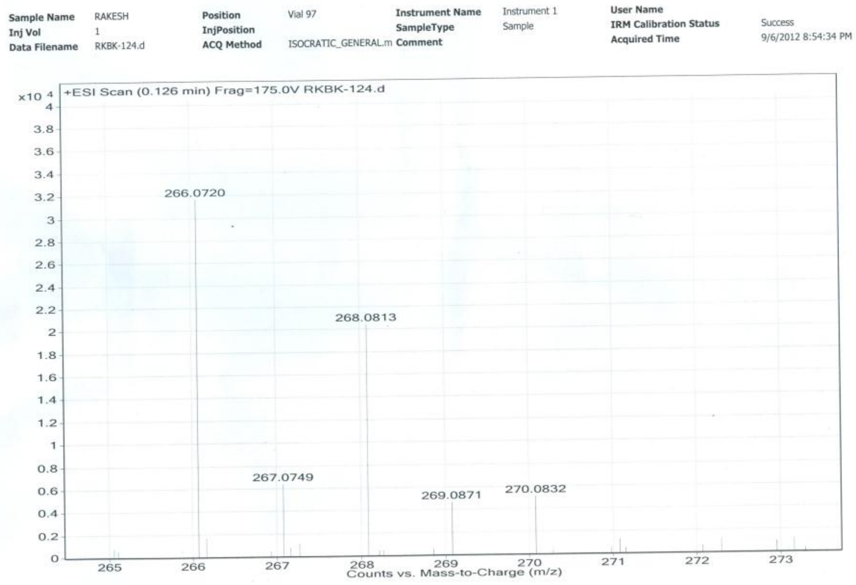

Figure 82: HRMS of 1p

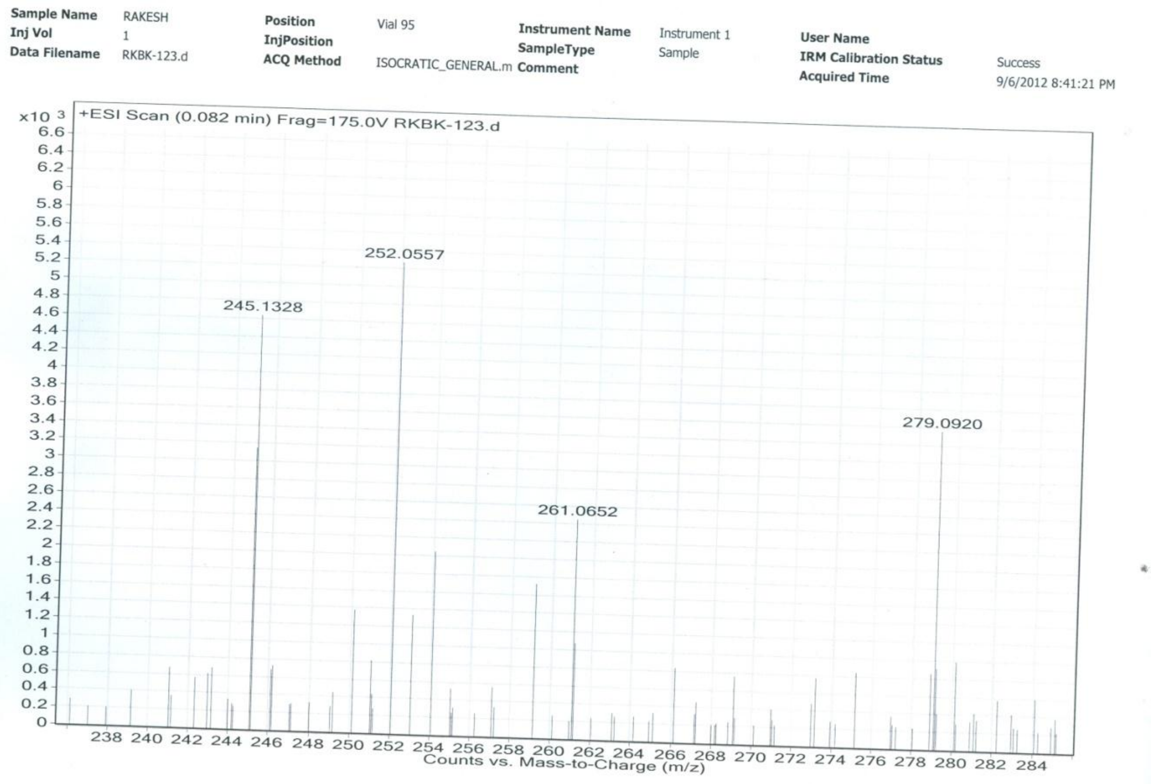

Figure 83: HRMS of 1q

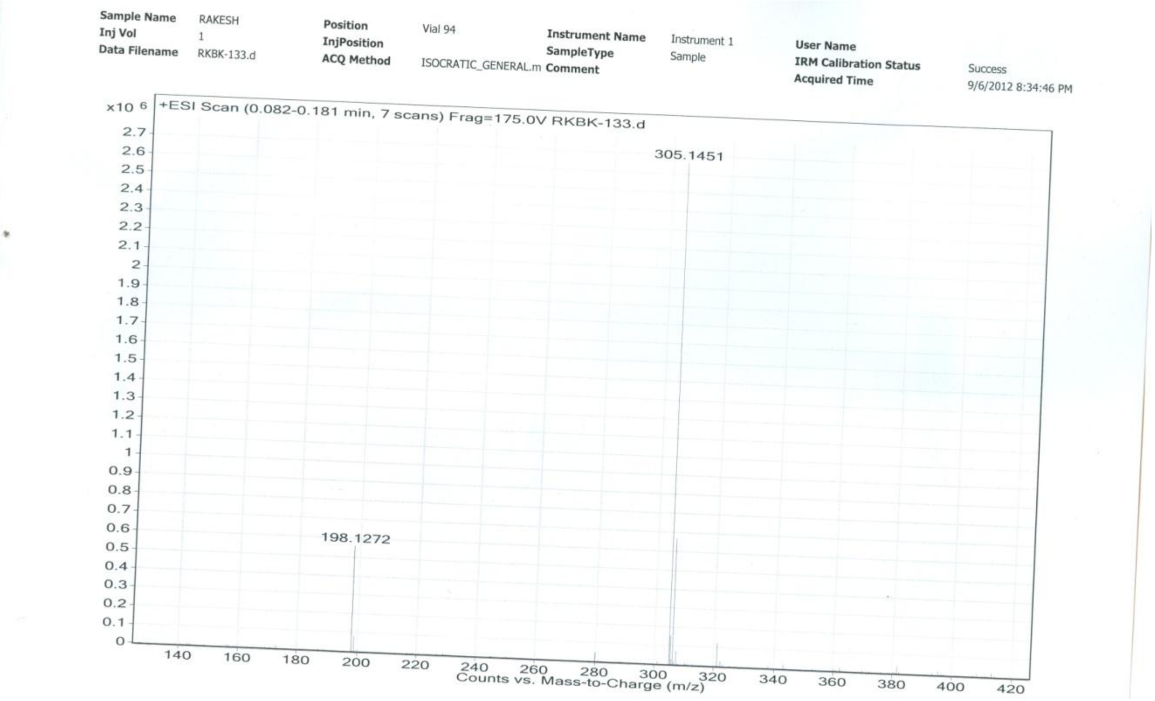

Figure 84: HRMS of 1r

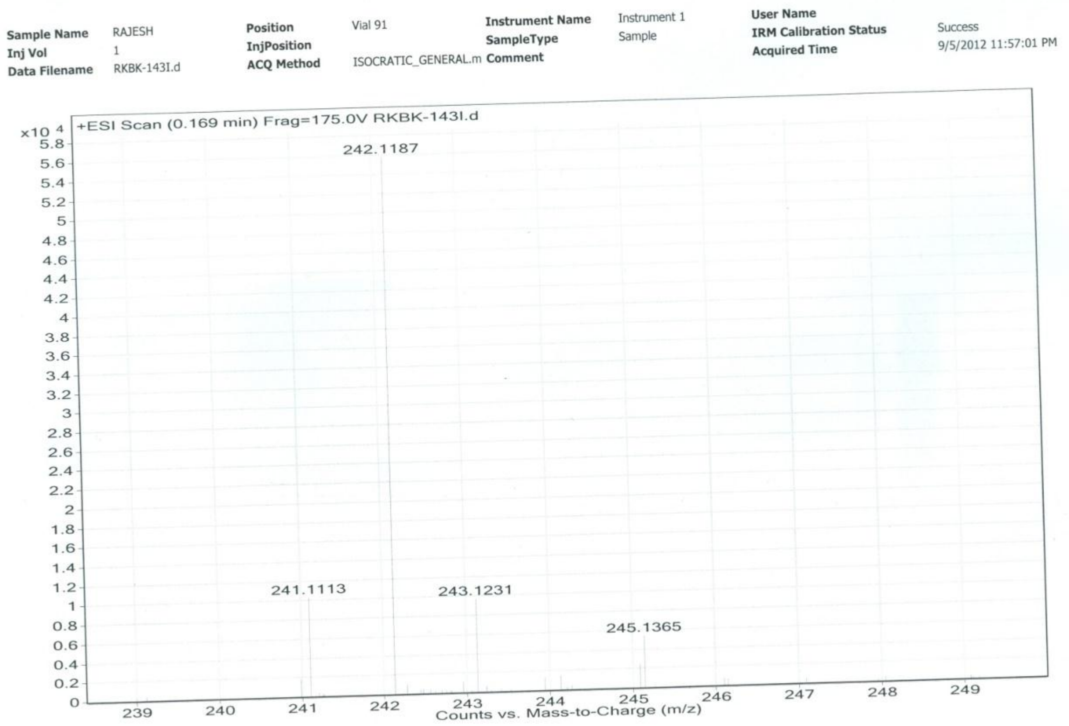

Figure 85: HRMS of 1s

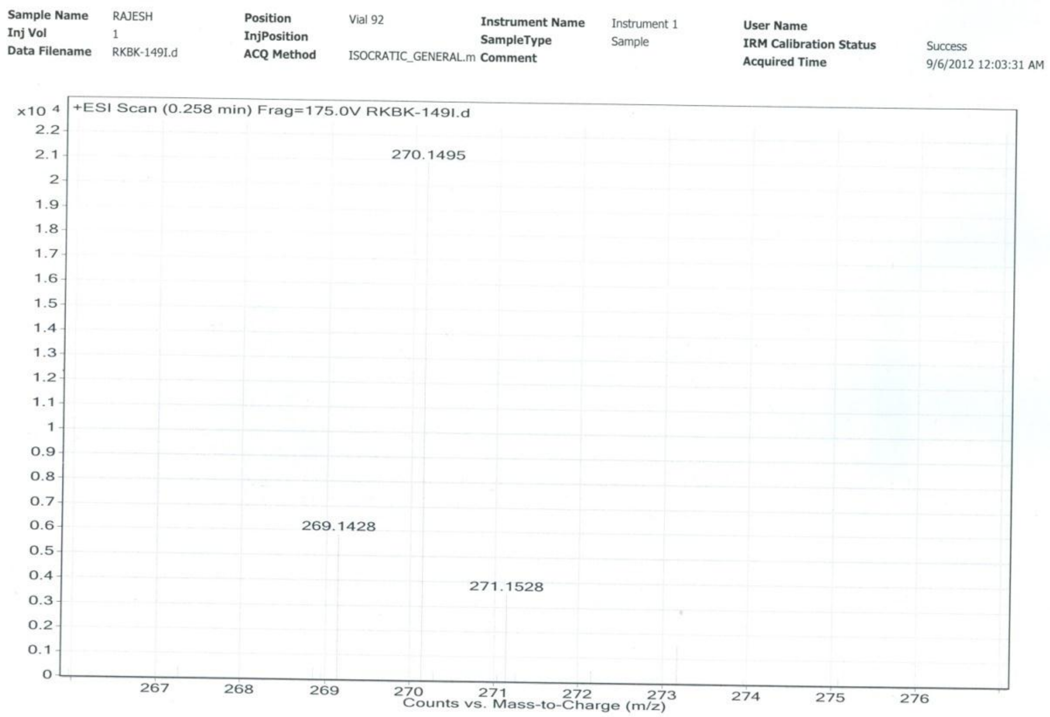

Figure 86: HRMS of 1t

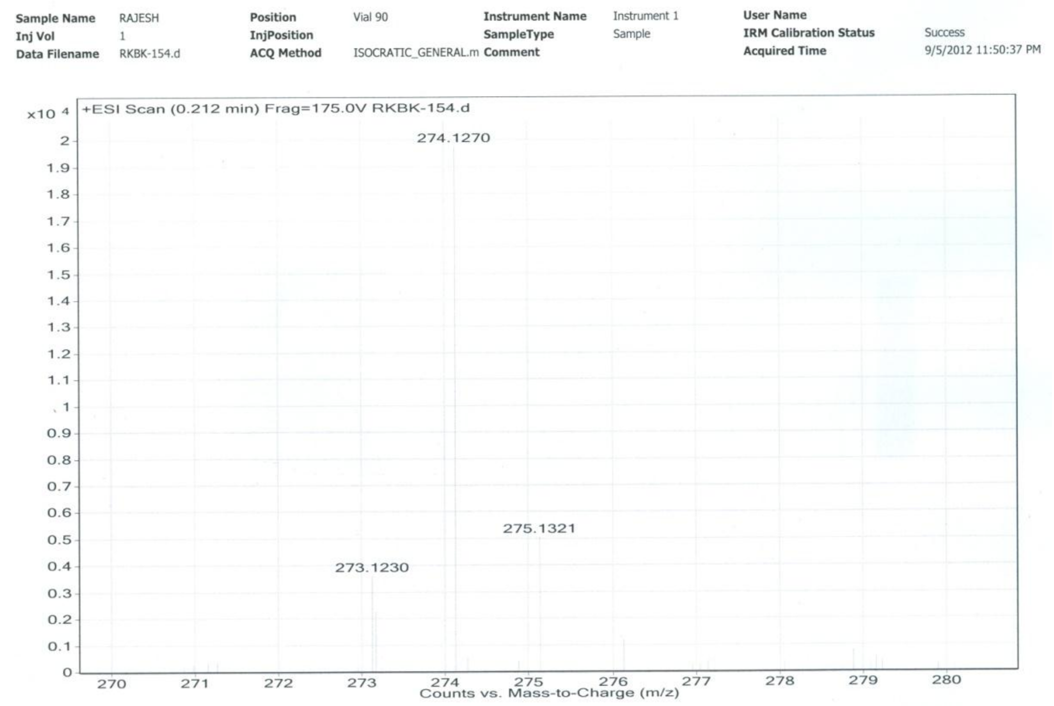

Figure 87: HRMS of 1v

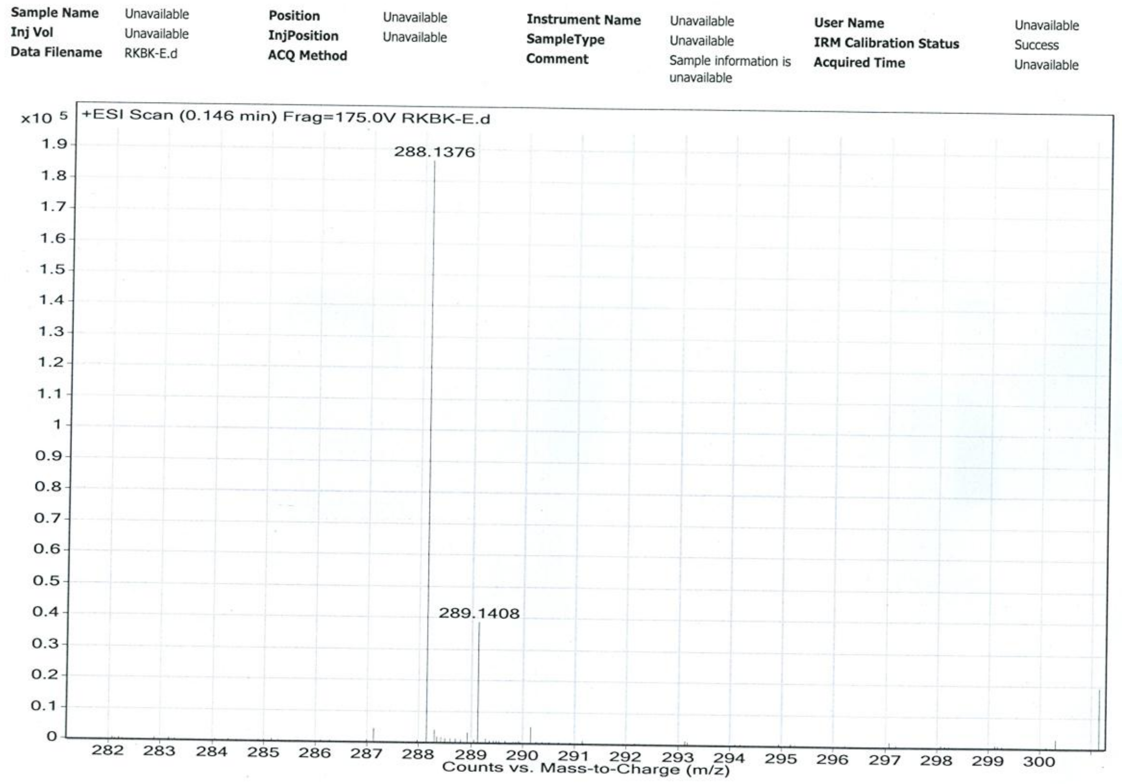

Figure 88: HRMS of 4a

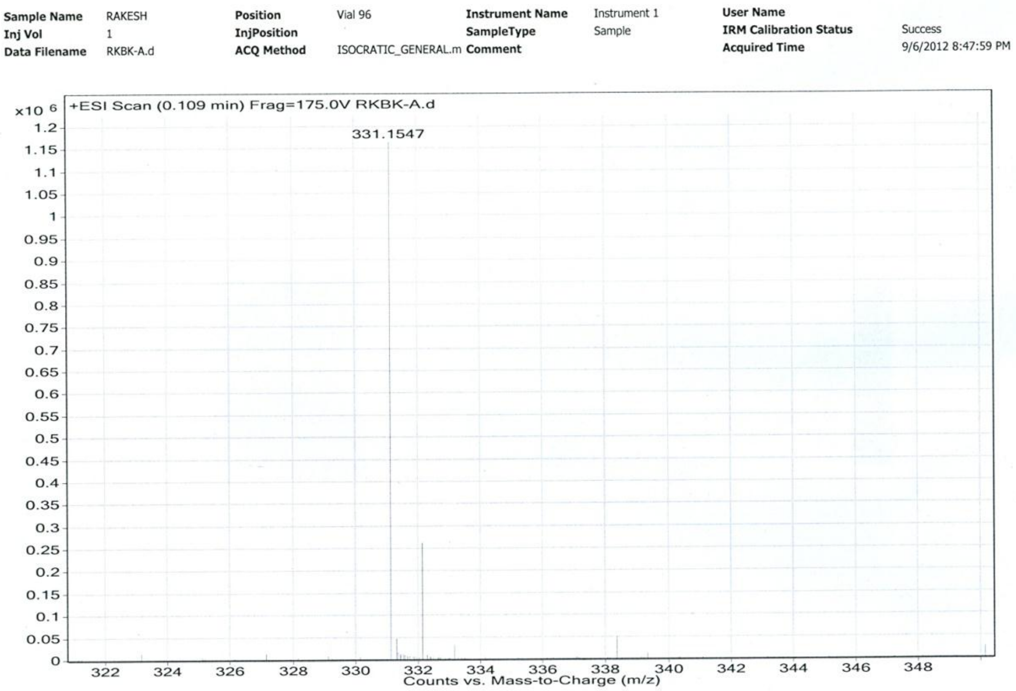

Figure 89: HRMS of 5a

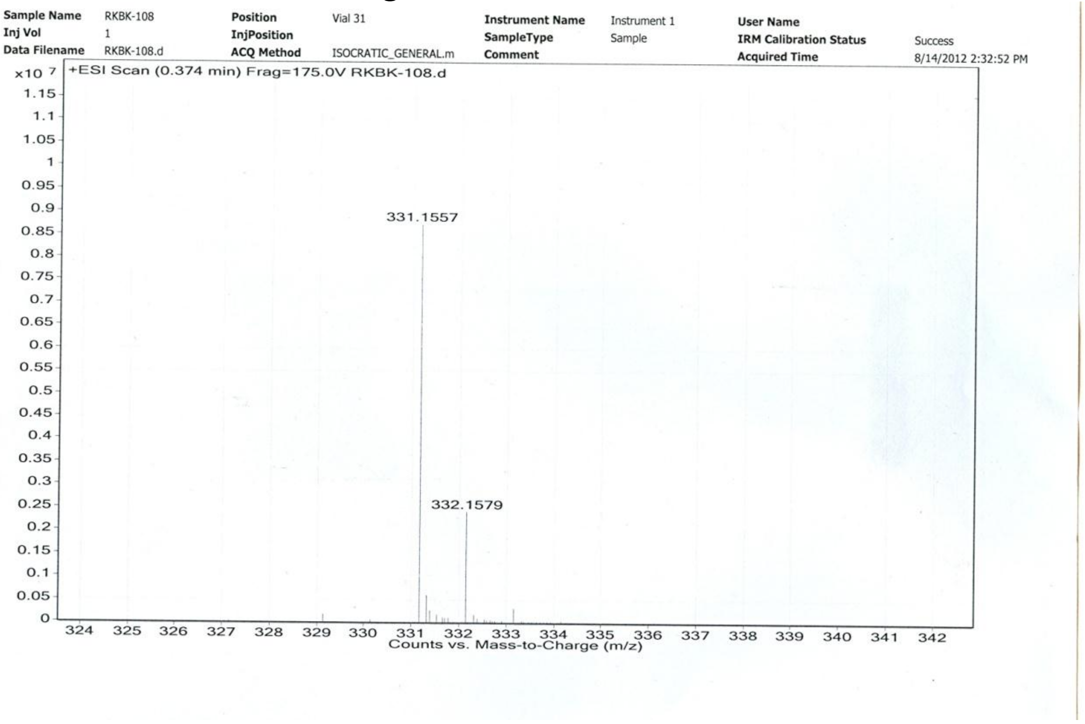

Figure 90: HRMS of 6a

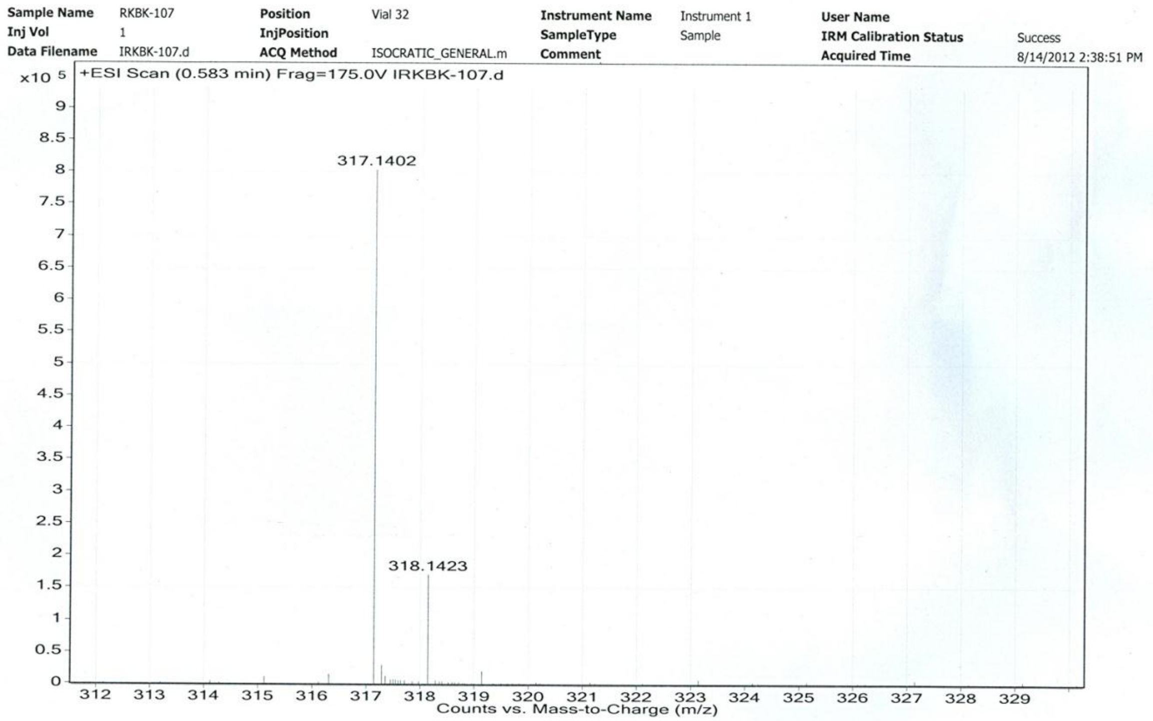

Figure 91: HRMS of 6b

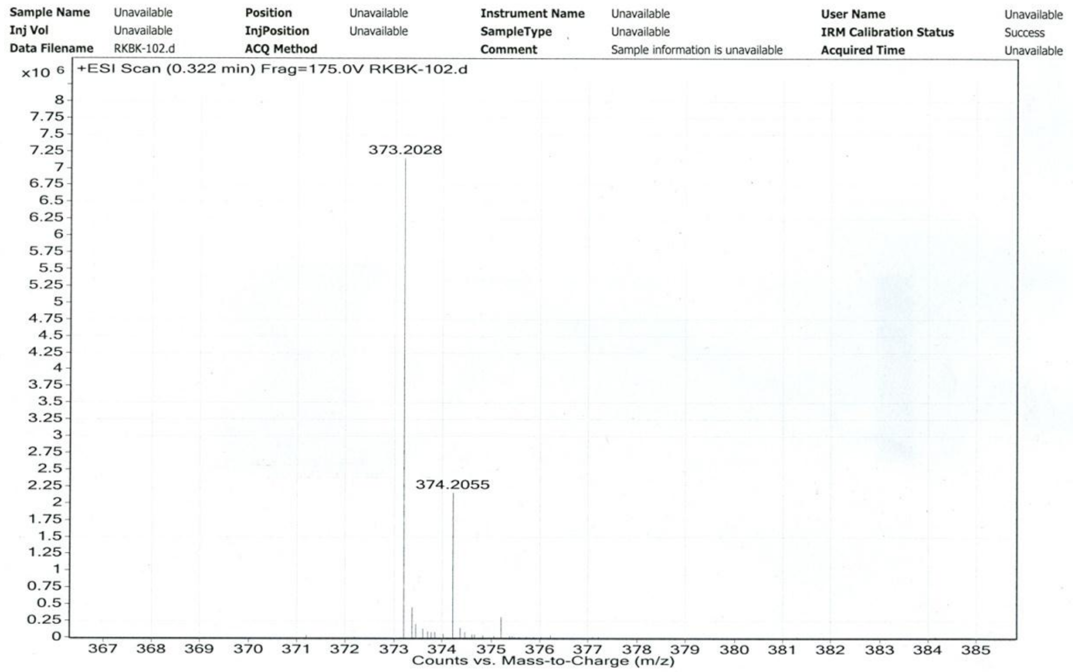

Figure 92: HRMS of 6c

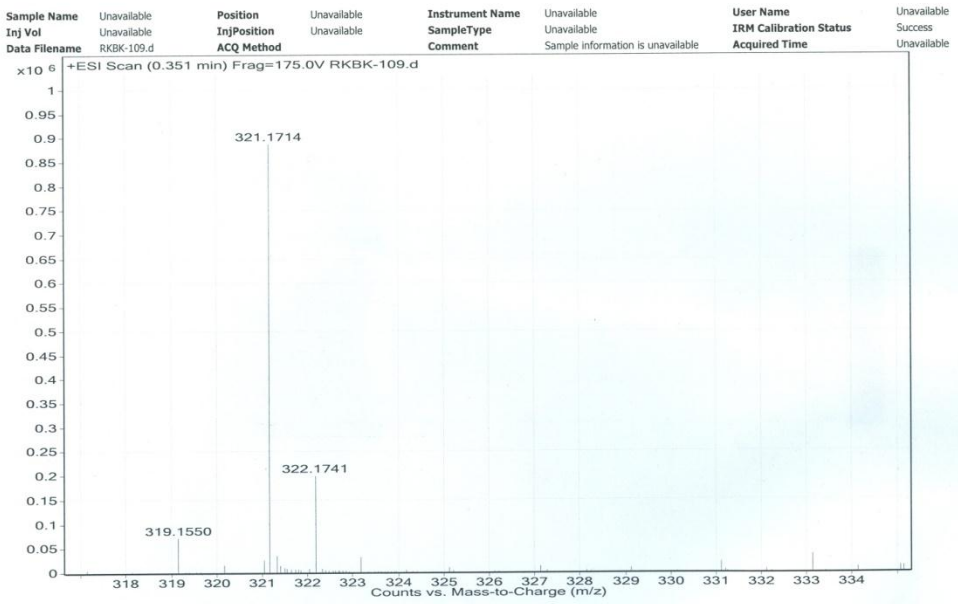

Figure 93: HRMS of 6d

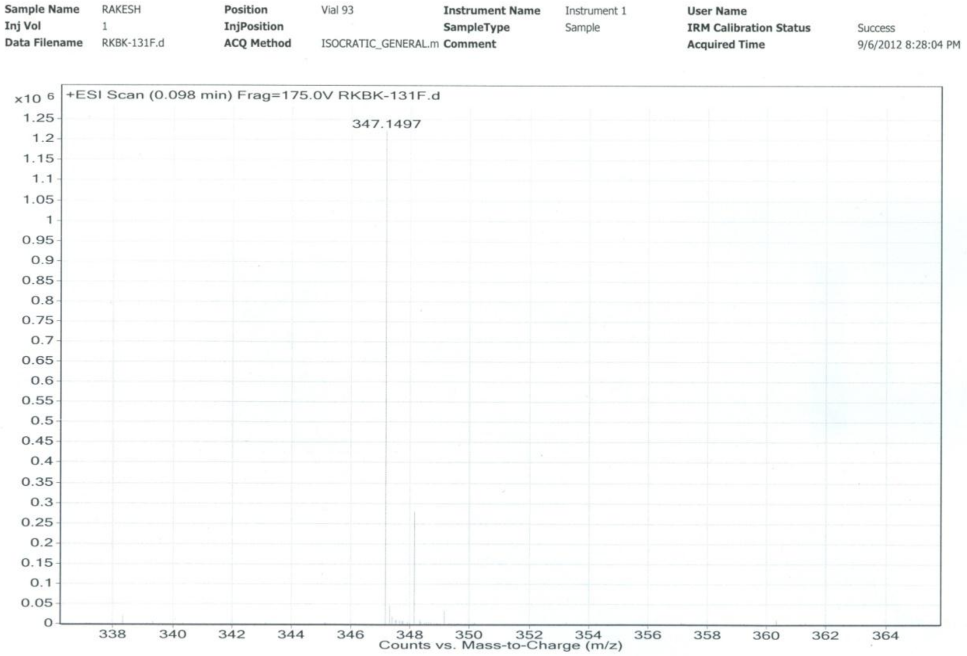

Figure 94: HRMS of 6e

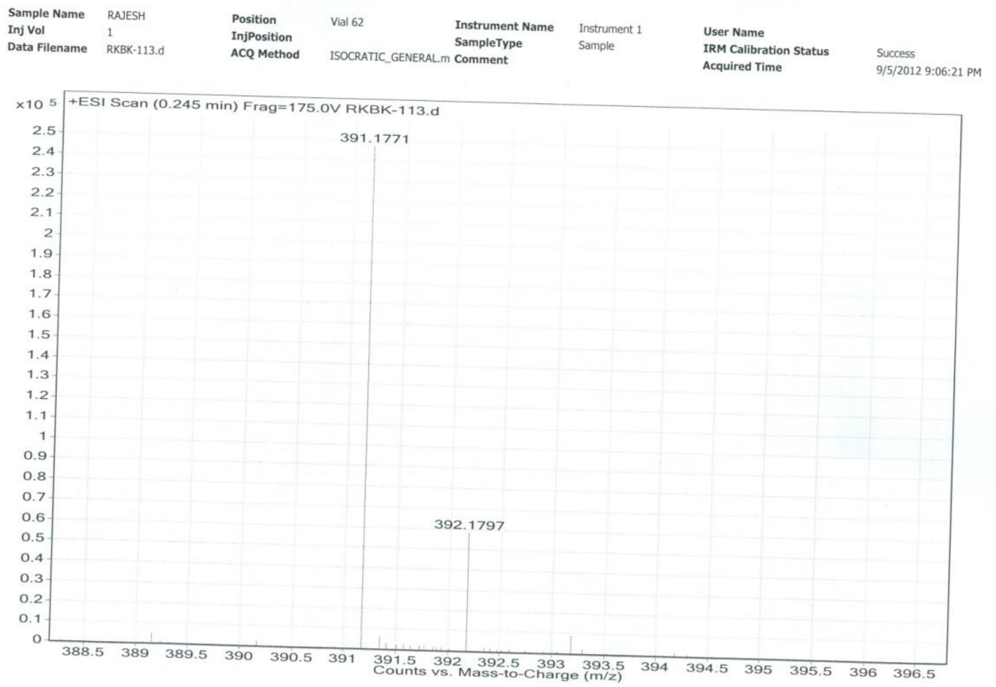

Figure 95: HRMS of 6f

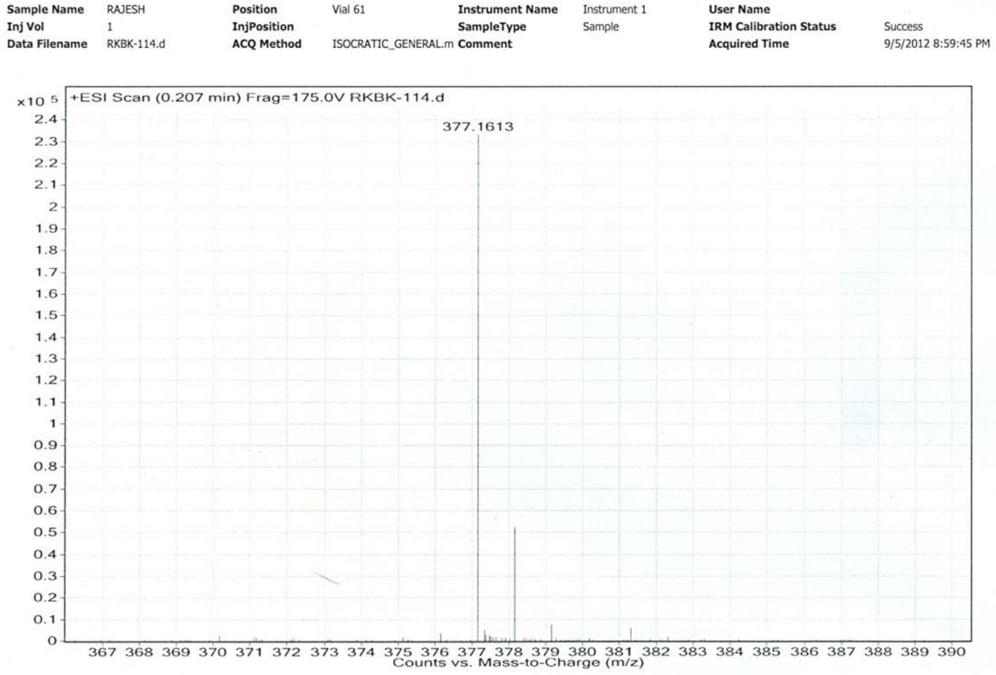

Figure 96: HRMS of 6g

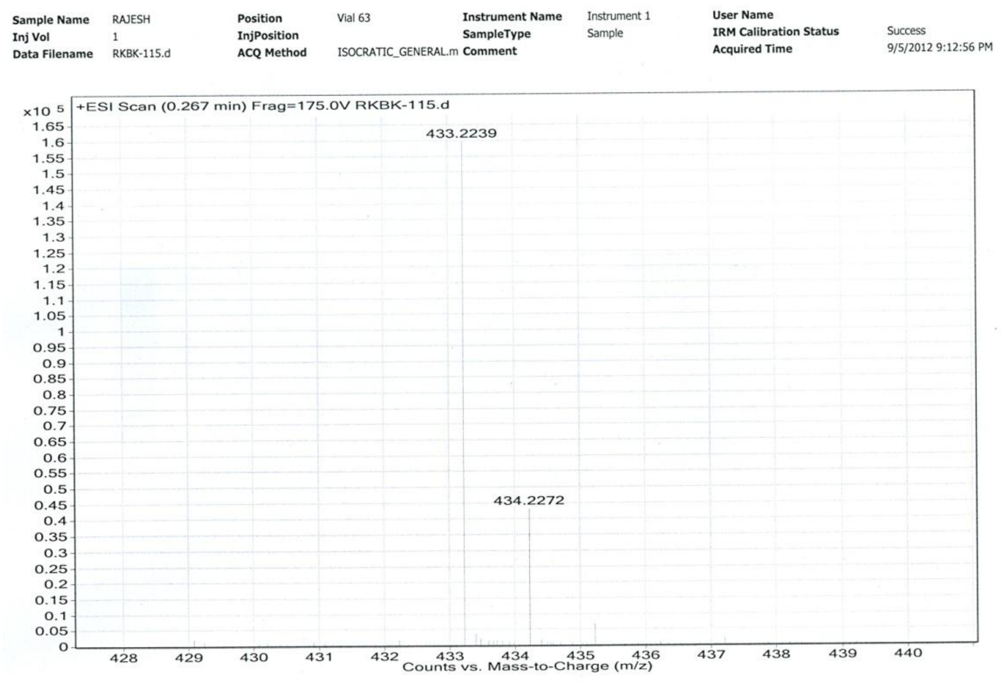

Figure 97: HRMS of 6h

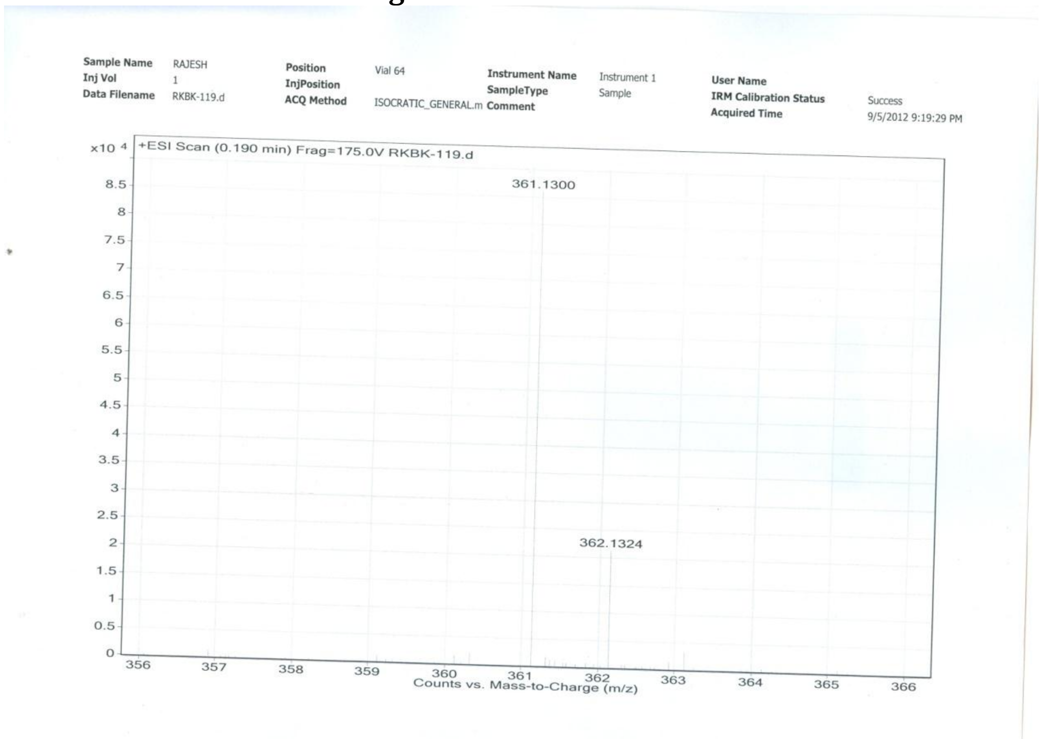

Figure 98: HRMS of 6i

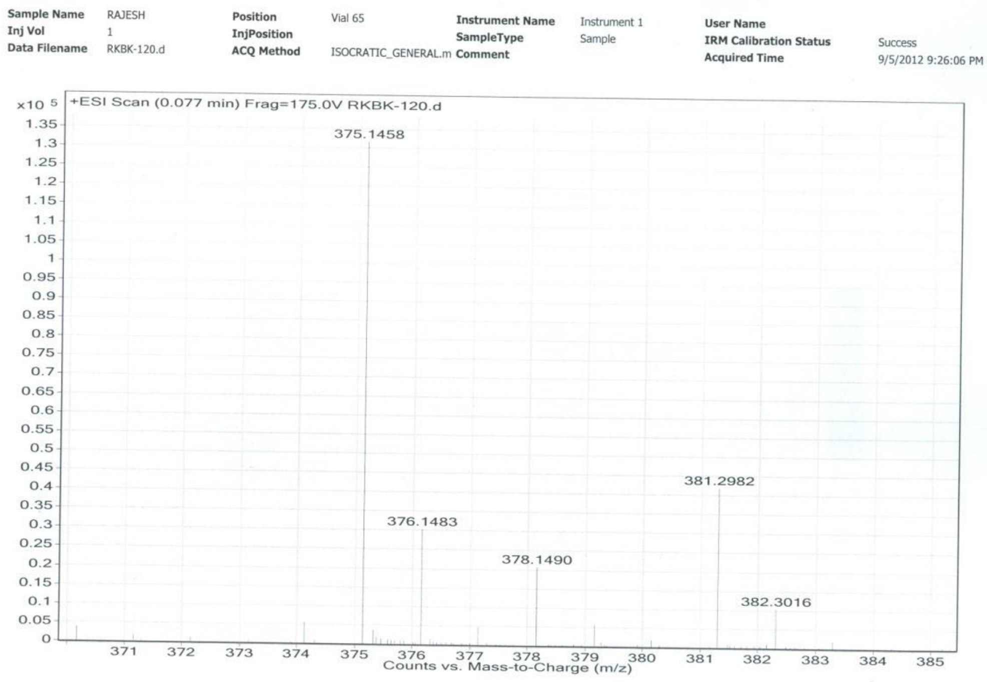

Figure 99: HRMS of 6j

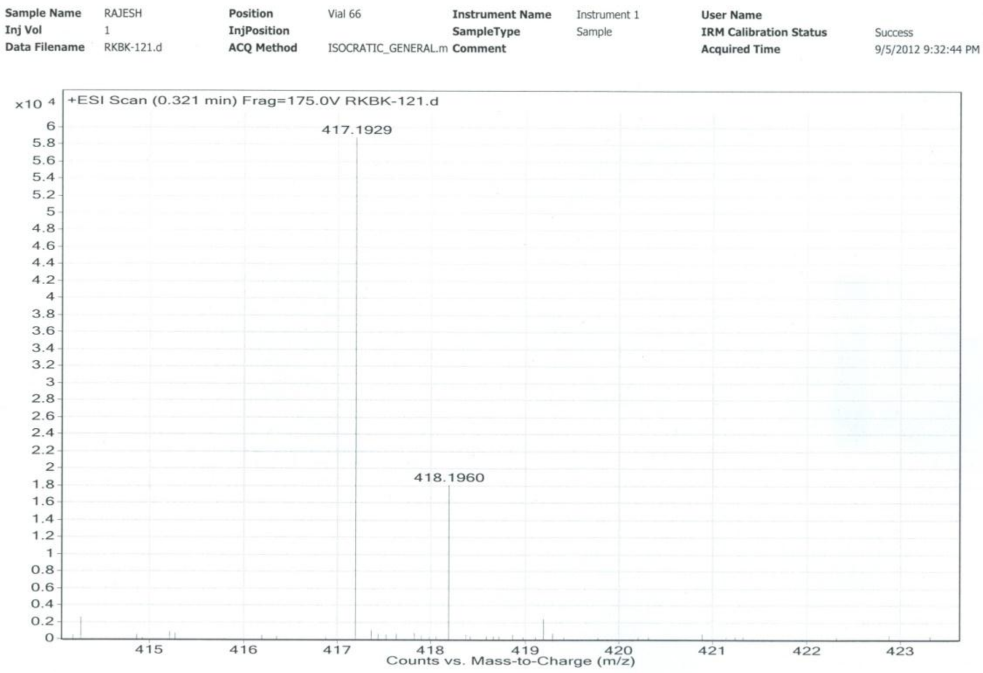

Figure 100: HRMS of 6k

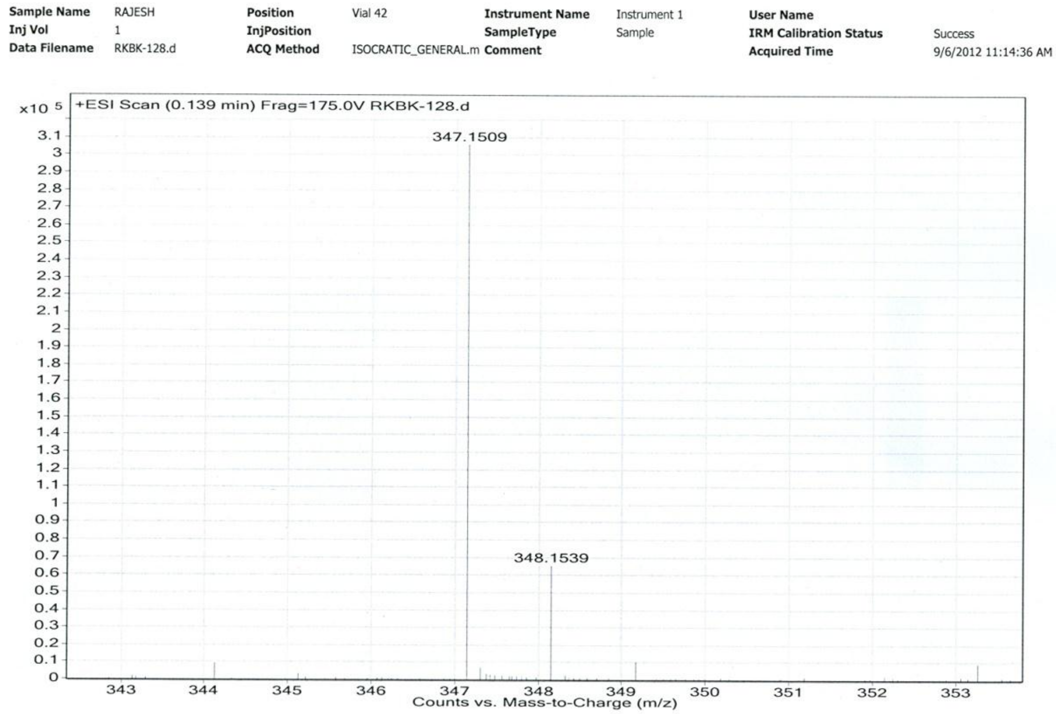

Figure 101: HRMS of 6l

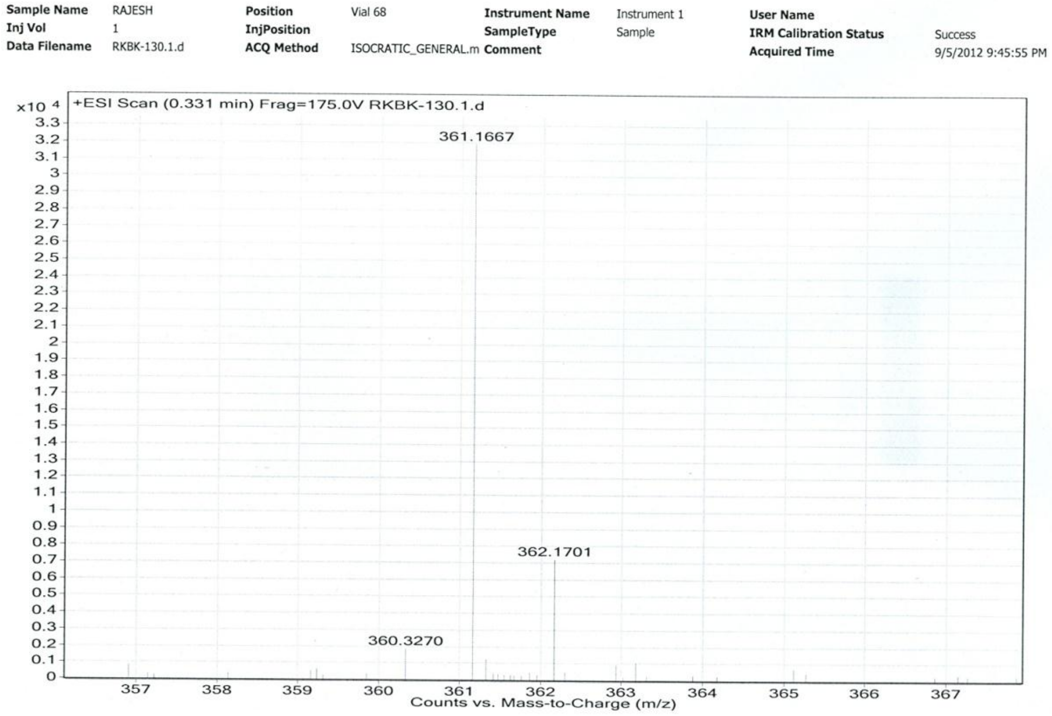

Figure 102: HRMS of 6m

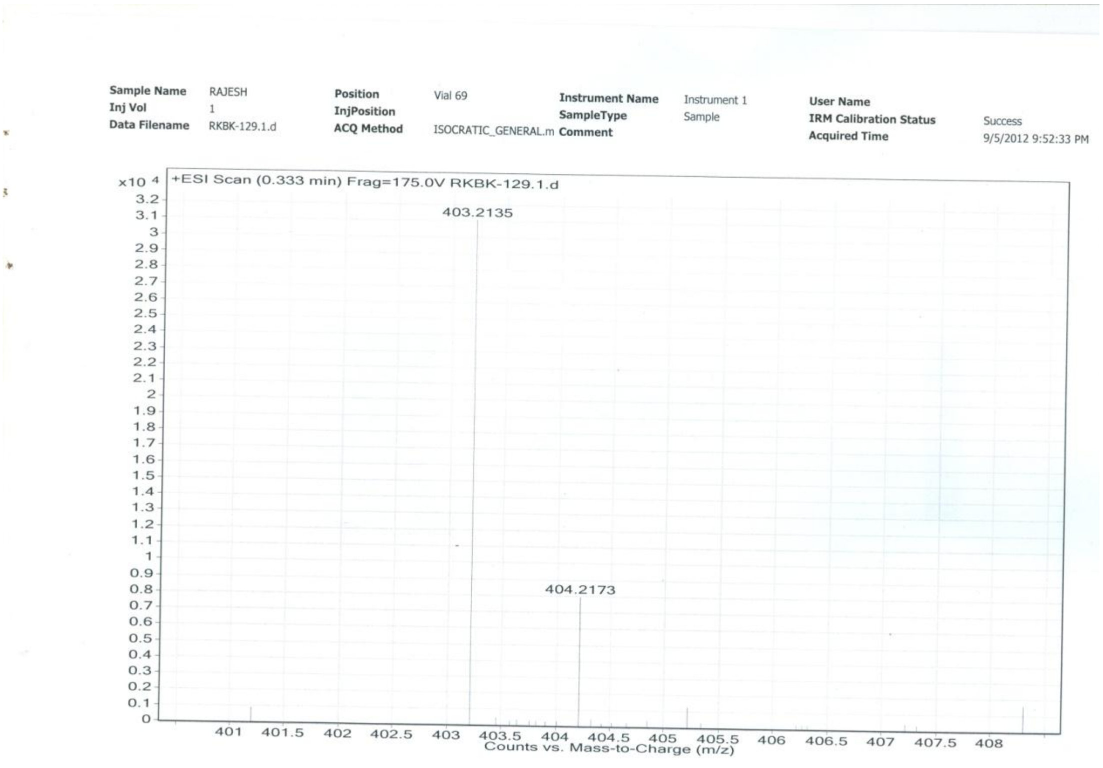

Figure 103: HRMS of 6n

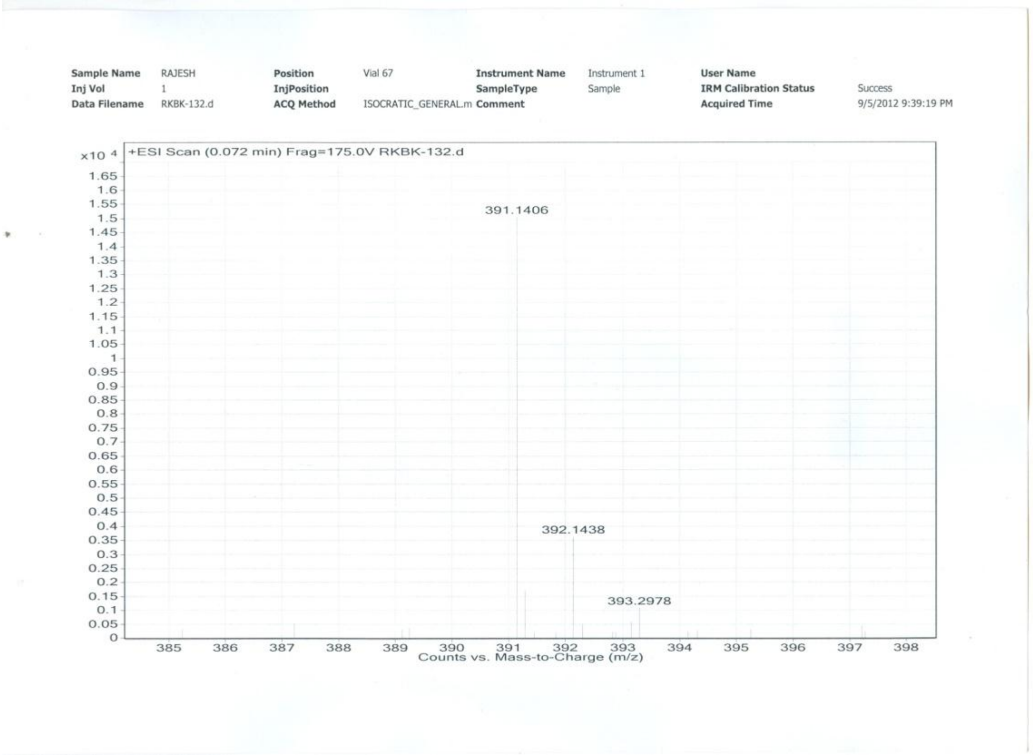

Figure 104: HRMS of 6o

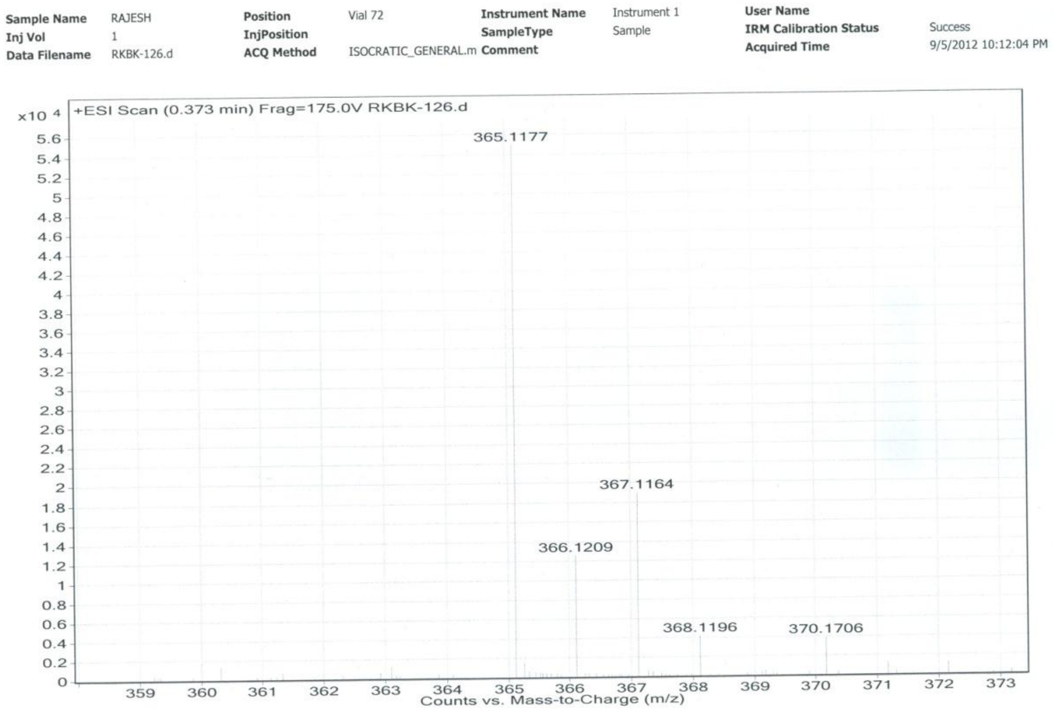

Figure 105: HRMS of 6p

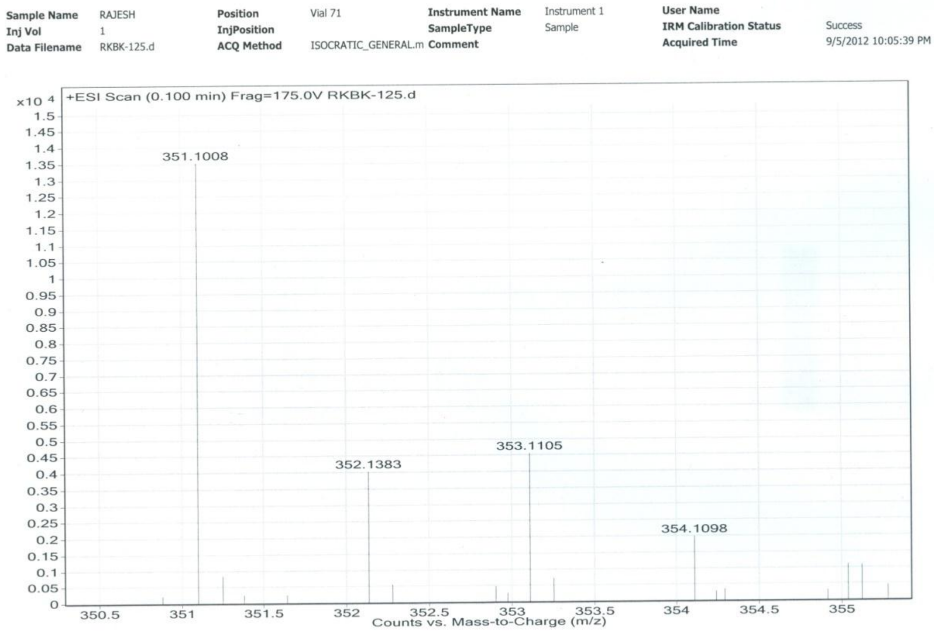

Figure 106: HRMS of 6q

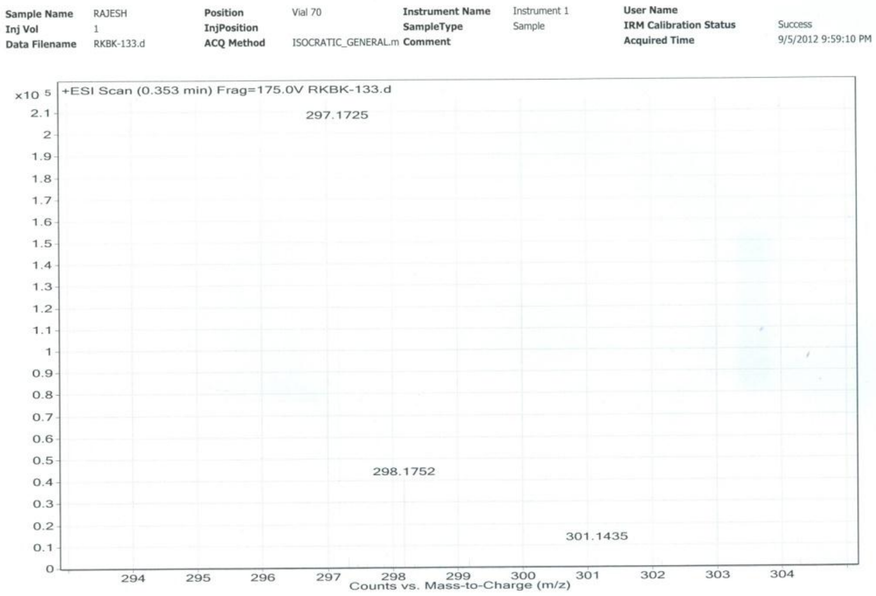

Figure 107: HRMS of 6r

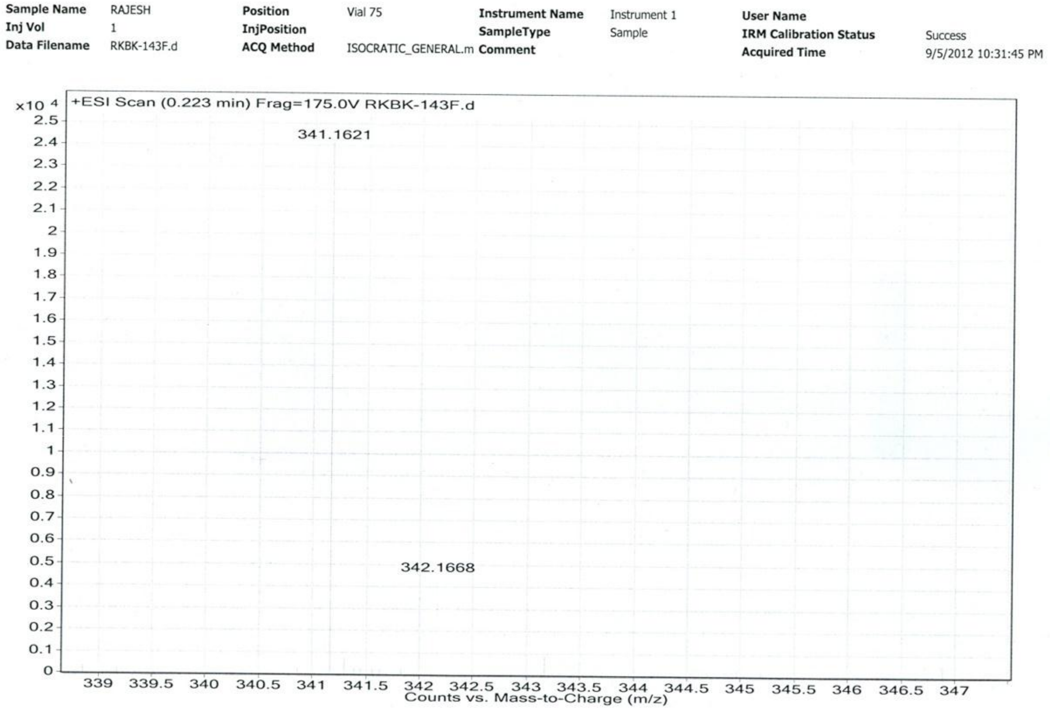

Figure 108: HRMS of 6s

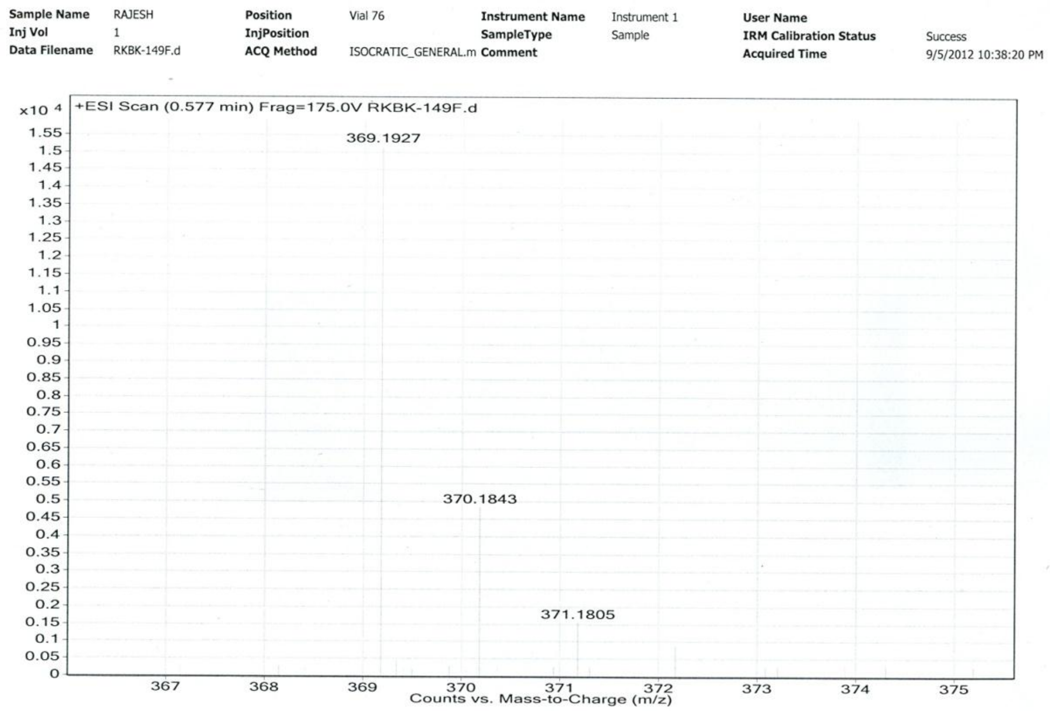

Figure 109: HRMS of 6t

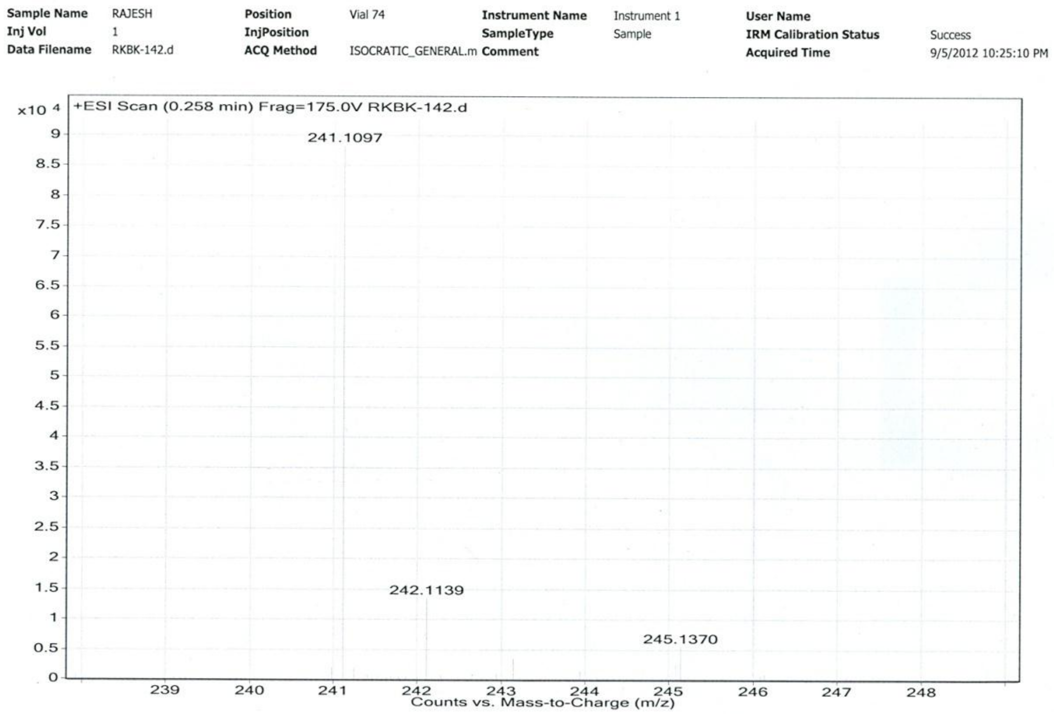

Figure 110: HRMS of 6u

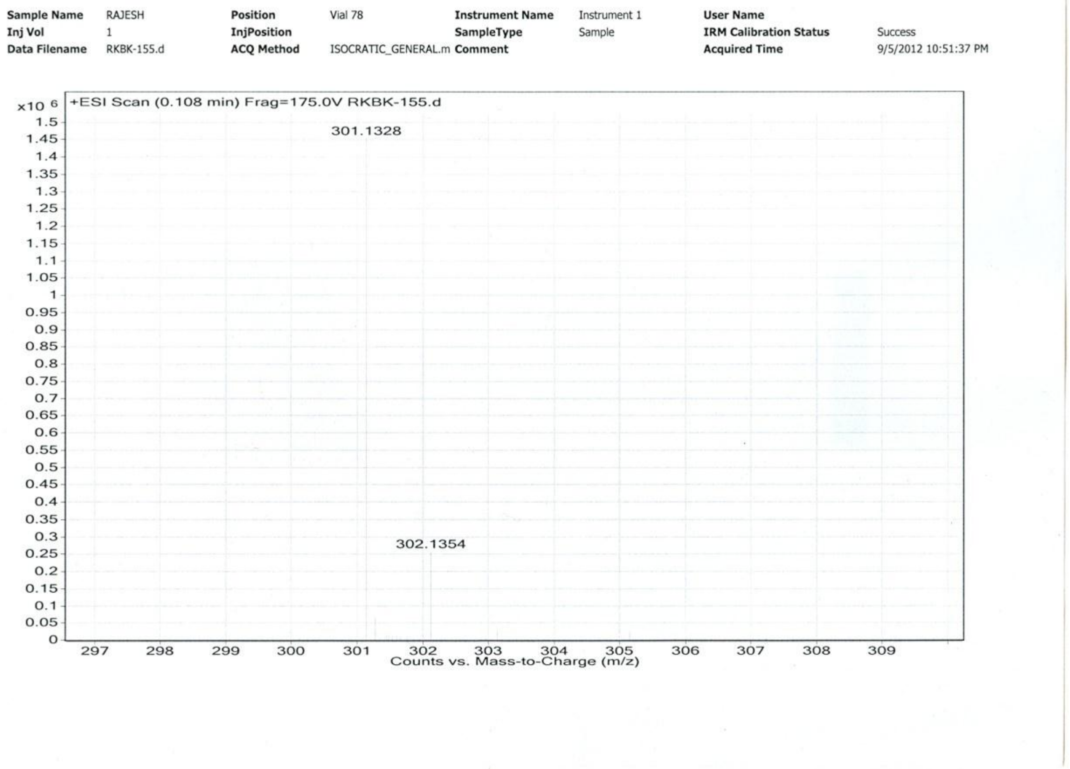

Figure 111: HRMS of 6v

Reference;

1.Arigela, R. K.; Mandadapu, A. K.; Sharma, S. K.; Kumar, B.; Kundu, B. *Org. Lett*. **2012**, *14*, 1804.

2. Nagamochi, M.; Fang, Y. Q.; Lautens, M. *Org. Lett*. **2007**, *9***,** 2955.

3. Fiandanese, V.; Bottalico, D.; Marchese G.; Punzi, A. *Tetrahedron* **2008**, *64*, 7301.

4. Soley, R.; Albericio, F.; Álvarez, M. *Synthesis* **2007**, 1559.
